# Genome-Wide Identification and Characterization of Hexokinase Genes in Moso Bamboo (*Phyllostachys edulis*)

**DOI:** 10.3389/fpls.2020.00600

**Published:** 2020-05-19

**Authors:** Wenqing Zheng, Yuan Zhang, Qian Zhang, Ruihua Wu, Xinwei Wang, Shengnian Feng, Shaoliang Chen, Cunfu Lu, Liang Du

**Affiliations:** ^1^Beijing Advanced Innovation Center of Tree Breeding by Molecular Design, Beijing Forestry University, Beijing, China; ^2^College of Biological Sciences and Technology, Beijing Forestry University, Beijing, China

**Keywords:** moso bamboo, hexokinase, sequence analysis, expression pattern, subcellular localization, HXK activity

## Abstract

Plant hexokinases (HXKs) are a class of multifunctional proteins that not only act as the enzymes required for hexose phosphorylation but also serve as sugar sensors that repress the expression of some photosynthetic genes when internal glucose level increases and regulators of cell metabolism and some sugar-related signaling pathways independent on their catalytic actives. The HXKs have been studied in many plants; however, limited information is available on HXKs of moso bamboo (*Phyllostachys edulis*). In this study, we identified and characterized 12 hexokinase genes in moso bamboo. Phylogenetic analysis revealed that the moso bamboo hexokinases (PeHXKs) were classifiable into five subfamilies which represented the three types of hexokinases in plants. Gene structure and conserved motif analysis showed that the PeHXK genes contained diverse numbers of introns and exons and that the encoded proteins showed similar motif organization within each subfamily. Multiple sequence alignment revealed that the PeHXK proteins contained conserved domains, such as phosphate 1 (P1), phosphate 2 (P2), adenosine, and a sugar-binding domain. Evolutionary divergence analysis indicated that the PeHXK, OsHXK, and BdHXK families underwent negative selection and experienced a large-scale duplication event approximately 19–319 million years ago. Expression analysis of the PeHXK genes in the leaf, stem, root, and rhizome of moso bamboo seedlings indicated that the PeHXKs perform pivotal functions in the development of moso bamboo. A protein subcellular localization assay showed that PeHXK5a, PeHXK8, and PeHXK3b were predominantly localized in mitochondria, and PeHXK8 protein was also detected in the nucleus. The HXK activity of the PeHXK5a, PeHXK8, and PeHXK3b was verified by a functional complementation assay using the HXK-deficient triple-mutant yeast strain YSH7.4-3C (*hxk1*, *hxk2*, and *glk1*), and the results showed that the three PeHXKs had the plant HXK-specific enzyme traits. The present findings would provide a foundation for further functional analysis of the PeHXK gene family.

## Introduction

In plants, sugars are produced predominantly in the leaves during photosynthesis. As one type of sugar, sucrose serves as the core primary metabolite for sink tissues, and as a signaling molecule that regulates plant growth and development (Aguilera-Alvarado and Sanchez-Nieto, 2017). Sucrose is mainly catalyzed by invertase or sucrose synthase into hexoses, including glucose and fructose, both of which must be phosphorylated by hexose-phosphorylating enzymes before further metabolism (Granot, 2008; David-Schwartz et al., 2013). In plants, only two kinds of hexose phosphorylating enzymes, hexokinases (HXKs) and fructokinases (FRKs), have been found (Dai et al., 1999). While HXKs phosphorylate a broad spectrum of hexoses such as glucose and fructose, FRKs specifically phosphorylate fructose (Dai et al., 2002). However, for HXKs in tomato, their affinities to glucose are much higher than that to fructose, and meanwhile, their affinity to fructose is much lower than that of plant FRKs, indicating plant HXKs are actually glucose phosphorylating enzymes (Granot, 2007; Aguilera-Alvarado and Sanchez-Nieto, 2017).

Using biochemical, genetic, or bioinformatics methods, plant HXK gene families have been identified in many species (Karve et al., 2010; Aguilera-Alvarado and Sanchez-Nieto, 2017), including *Arabidopsis thaliana* (Karve et al., 2008), *Physcomitrella patens* (Olsson et al., 2003), *Lycopersicon esculentum* (Damari-Weissler et al., 2006; Granot, 2007), *Solanum lycopersicum* (Kandel-Kfir et al., 2006), *Nicotiana tabacum* (Giese et al., 2005), *Oryza sativa* (Cho et al., 2006), *Solanum tuberosum* (Jon et al., 2002), *Vitis vinifera* (Fei et al., 2013), *Zea mays* (Zhang et al., 2014), *Manihot esculenta* Grantz (Geng et al., 2017) *Camellia sinensis* (Li et al., 2017), *Brassica napus* (Wang et al., 2018), and pear (*Pyrus* × *bretschneideri*) (Zhao et al., 2019). On the basis of N-terminal amino acid sequences, the hexokinases are classified into two main types (type A and type B), and two rare types (type C and type D) (Aguilera-Alvarado and Sanchez-Nieto, 2017). The type A HXKs, such as AtHXK3, OsHXK4, and NtHXK2, contain plastid-signaling peptides that indicate their potential localization to plastids (Giese et al., 2005; Cho et al., 2006; Karve et al., 2008, 2010). Somewhat surprising is that type A HXKs have not been predicted in maize or sorghum (both C4 grasses) (Karve et al., 2010). Unlike type A, type B HXKs generally have a highly hydrophobic transmembrane helix associated with mitochondria (Aguilera-Alvarado and Sanchez-Nieto, 2017). However, some type B HXKs may also be localized in the nucleus because a nuclear localization signal is present close to the transmembrane helices (Cho et al., 2009). Type C HXKs, which lack plastid-signaling peptides and membrane-anchored domains, have presently been identified only in *Physcomitrella patens* and monocotyledonous plants (Cheng et al., 2011; Nilsson et al., 2011).

As ancient and conserved hexokinases, the plant HXKs catalyze hexose phosphorylation, sense glucose level, and correlate with multiple signaling pathways influencing plant growth and development (Rolland et al., 2006; Claeyssen and Rivoal, 2007; Aguilera-Alvarado and Sanchez-Nieto, 2017). In *Arabidopsis*, AtHXK1 is the best-characterized glucose sensor and is involved in plant development and stress response (Moore et al., 2003; Kim et al., 2006; Rolland et al., 2006; Sarowar et al., 2008). Moreover, AtHXK1 forms a glucose signaling complex with VHA-B1 and RPT5B that directly modulates the transcription of specific target genes (Cho et al., 2007). Additional functions of HXK1 have been elucidated in many other species. For example, overexpression of *AtHXK1* in guard cells affected stomatal closure in citrus (Kelly et al., 2013; Lugassi et al., 2015). In tomato, overexpression of *AtHXK1* resulted in reduced photosynthesis, slower growth, and induction of senescence, which is indicative of its function in photosynthetic tissues (Dai et al., 1999). Overexpression of pear *PbHXK1* in tomato modulated the sugar content and affected plant growth (Zhao et al., 2019). In *Nicotiana benthamiana*, virus-induced gene silencing of *NbHXK1* caused cell death, implying that HXK1 plays a role in the process of cell death (Kim et al., 2006). In apple, MdHXK1 may phosphorylate MdbHLH3 to regulate anthocyanin biosynthesis, and MdNHX1 improves salt tolerance (Hu et al., 2016; Sun et al., 2018). In sunflower (*Helianthus annuus L.*), *HaHXK1* transcript levels were higher in developing seeds than in photosynthetic tissues and the highest HXK activity was detected in the early stages of accumulation of reserve compounds, lipids, and proteins in the seed (Troncoso-Ponce et al., 2011). In addition to HXK1, the functions of some of the other HXK members have been revealed. In rice, OsHXK5 and OsHXK6 function as glucose sensors and overexpression of *OsHXK5* and *OsHXK6* caused growth retardation in response to glucose treatment (Cho et al., 2009). Reduced *OsHXK10* expression in rice resulted in abnormal dehiscence of the anthers in some flowers (Xu et al., 2008). Moreover, plant HXKs also play a regulatory role in interactions between sugars (nutrition) and phytohormones (León and Sheen, 2003; Moore et al., 2003).

Moso bamboo (*Phyllostachys edulis*) is a member of the Gramineae family and is widely distributed in China and the world with high economic and ecological value (Peng et al., 2013; Zhang et al., 2018). Similar to many other bamboo species, moso bamboo is a perennial with a large genome and complex phenotypes, which is possibly a cause of the slow progress in research on biological functions in the species. In recent years, the publication of the moso bamboo genome (Peng et al., 2013; Wang et al., 2018) and rapid development of plant omics technology have presented the opportunity to explore genetic functions in bamboo, and some gene families have been uncovered (Liu et al., 2017; Cheng et al., 2018; Gao et al., 2018; Hou et al., 2018; Li et al., 2018). With aspect to all the traits of bamboo, the most attractive one is the growth rate that the shoot can grow as fast as one meter per day at peak growth. In order to understand the fast growth, some previous studies have characterized the shoot growth and anatomy that provide insight into the fast-growth bamboo (Lin et al., 2002; Li et al., 2018; Wei et al., 2018). However, the molecular mechanism underlying the fast-growth of bamboo shoot is still unclear. HXKs, which act as catalysts of the first essential step in glucose metabolism, have emerged as important enzymes that mediate sugar sensing and related signals in plant growth (Claeyssen and Rivoal, 2007; Aguilera-Alvarado and Sanchez-Nieto, 2017). With a view that HXKs -related pathways are important for sugar signal transduction which depends on phosphorylated hexoses and intermediate glycolytic products (Aguilera-Alvarado and Sanchez-Nieto, 2017), the plant HXKs are considered to have the function of regulating plant growth and the potential of promoting biomass. In the present study, we identified and characterized PeHXKs in moso bamboo. First, we searched against the bamboo genome database using HXK protein sequences of *Arabidopsis* and identified hexokinase genes by means of subsequent bioinformatic analysis and experimental verification. We characterized the PeHXKs by investigation of phylogenetic relationships, gene structure, motif analysis, physicochemical properties of the proteins, and subcellular protein localization. Quantitative real-time PCR (qRT-PCR) was used to analyze gene expression patterns and functional complementation in an HXK-deficient yeast strain (YSH7.4-3C) was used to verify the hexokinase activity of PeHXKs. In total, we identified 12 PeHXKs, for which we assessed the conservation in gene and protein structure and the expression profiles in moso bamboo tissues. Also, the HXK activity of the PeHXK5a, PeHXK8, and PeHXK3b was verified. The results presented herein provide a reference for future studies of the PeHXK gene family, especially for the regulation of fast growth and rapid-accumulated biomass in moso bamboo.

## Materials and Methods

### Identification of HXKs in Moso Bamboo

To identify HXK genes of moso bamboo, we used *Arabidopsis* HXK protein sequences (TAIR^1^) as query sequences for a BLAST search of the moso bamboo database (Zhao et al., 2018^2^) using a threshold *P*-value < 10^–5^. Fifteen candidate HXK sequences were obtained. Three sequences were discarded as a result of a search for conserved domains within the amino acid sequences of the National Center for Biotechnology Information (NCBI) Conserved Domains database^3^ and the EMBL-EBI Pfam database^4^. The number of amino acids and length of the coding sequence (CDS) were characterized, and the theoretical isoelectric point (pI) and molecular weight of amino acids were calculated using the ProtParam online tool^5^ (Gasteiger et al., 2003).

Using the genome and CDS sequences of candidate PeHXKs from the moso bamboo genome database, we mapped the genetic structure with the Gene Structure Display Server 2.0 (GSDS tool^6^) (Guo et al., 2007). Motif analysis was carried out using MEME^7^ with a maximum number of 10 motifs and using other default settings (Liu et al., 2018). In the bamboo genome database, we first determined the relative position of each gene on each chromosome, then the location of the PeHXK gene on the chromosome was mapped using MapDraw (Liu and Meng, 2003).

### Sequence Alignment and Phylogenetic Tree Construction

We downloaded protein sequences of *Arabidopsis* HXKs from the TAIR database^1^, rice HXKs from the NCBI database^8^, and *Populus trichocarpa* and *Brachypodium distachyon* HXKs from the Phytozome database^9^. The sequences were aligned using MUSCLE, and evolutionary trees were constructed using the neighbor-joining method with MEGA (Liu et al., 2018). The reliability of the tree topology was assessed by means of a bootstrap analysis with 1000 replications. Multiple amino acid sequences of several conserved binding domains were aligned by using SeaView 4 software (Gouy et al., 2010).

### Identification of Orthologs and Calculation of Ka and Ks

On the basis of previous reports, orthologs were identified by pairwise alignment (Blanc and Wolfe, 2004). The synonymous substitution rate (Ks) and non-synonymous substitution rate (Ka) were calculated using DnaSP 5 software (Rozas, 2009). We calculated the evolutionary divergence time using the formula T = Ks/2λ (λ = 6.5 × 10^–9^) as reported previously (Peng et al., 2013). In general, Ka/Ks > 1 indicates the positive selection, Ka/Ks = 1 indicates the neutral selection, and Ka/Ks < 1 suggests negative or stabilizing selection (Juretic et al., 2005).

### Plant Material, RNA Extraction, and Quantitative Real-Time PCR (qRT-PCR)

Moso bamboo seeds were collected from Gongcheng Yao Autonomous County, Guangxi Zhuang Autonomous Region, China. The seeds were heated for 1 day in a water bath at 42°C in 200 mg/L gibberellic acid (GA) solution before sowing. The seeds were sown by lightly covering with a mixture of vermiculite and vegetative soil (1:1) and then covered with plastic wrap, and were incubated in a greenhouse at 26°C under a 16 h/8 h (light/dark) photoperiod. The seeds germinated after approximately 1 week and grew to about 10 cm in height after 3 months. The uppermost four leaves (from the shoot apex), stems (including the internodal region and nodes of the whole seedling), roots, and rhizomes were used for the extraction of total RNAs. Tissue samples were collected and immediately placed in liquid nitrogen, and then stored at −80°C until use. Total RNAs were extracted from the samples using the RNAprep Pure Plant Kit (TIANGEN – DP419, Beijing, China). The first-strand cDNA was synthesized using the TransScript^®^ One-Step gDNA Removal and cDNA Synthesis SuperMix (TransGen – AT311, Beijing, China) in accordance with the manufacturer’s instructions. The first-strand cDNA was used as the template in qRT-PCR analyses. The primers (Supplementary Table S2) used for qRT-PCR analyses were designed using the NCBI Primer-BLAST tool^10^ with a PCR product size of 150–250 bp. An intrinsic membrane protein-encoding gene, *Tonoplast Intrinsic Protein 41* (*TIP41*), was used as the internal reference gene (Fan et al., 2013). The qRT-PCR protocol was 94°C for 30 s, then 40 cycles of 94°C for 5 s and 60°C for 30 s, and used the TransStart^®^ Top Green qPCR SuperMix (TransGen – AQ131, Beijing, China). The values of expression were calculated from three independent biological repeats, each of which was the average of three technical repeats.

### Subcellular Localization of PeHXK Proteins

The full-length CDS region of individual PeHXK genes was integrated into the pCambia2300 vector [containing the green fluorescent protein (GFP) sequence]. The full-length CDS region of individual PeHXK genes was amplified by PCR using primers LP (*PeHXK5a*: CAGACAGTGATGGGGAAGGC; *PeHXK8*: ATGGCCGCAGCTGCGGTCGCAAT; *PeHXK3b*: ATGGTCGTTGAGATGCACGC) and RP (*PeHXK5a*: GTCGAC CTCGGCATACTGAGAGTGC; *PeHXK8*: CTGTTGCTC AACATACTTGTACTG; *PeHXK3b*: TATGGAACCTCCTTG TTGCTGTCTA). The GFP sequence was attached to the C-terminus of the target PeHXK protein. The pCambia2300-GFP plasmid was then introduced instantaneously into tobacco leaves by transformation mediated by *Agrobacterium tumefaciens* strain GV3101. After 3 days, transformed tobacco leaves were observed with a confocal laser scanning microscope. Before the observation of GFP signals, the mitochondria were stained with 500 nM MitoTracker^®^ Red dye by incubation at 37°C for approximately 50 min.

### Yeast Complementation Experiments

The yeast triple mutant YSH7.4-3C is deficient in *HXK1*, *HXK2*, and *GLK1* (Geng et al., 2017) and was kindly provided by Professor Xinwen Hu. The full-length CDS of individual PeHXK genes was integrated into the pDR195 vector (including URA3 as a selection marker) by homologous recombination (Geng et al., 2017). The full-length CDS region of individual PeHXK genes was amplified by PCR using primers LP (*PeHXK5a*: CAGACAGTGATGGGGAAGGC; *PeHXK8*: ATGGCCGCAGCTGCGGTCGCAAT; *PeHXK3b*: ATGGTCGTTGAGATGCACGC) and RP (*PeHXK5a*: GTCGAC CTCGGCATACTGAGAGTGC; *PeHXK8*: CTGTTGCTCAACA TACTTGTACTG; *PeHXK3b*: TATGGAACCTCCTTGTTGCTG TCTA). Given that the yeast triple mutant was unable to utilize glucose or fructose as carbon sources, the reproducible medium was composed of 2% peptone, 1% yeast extract, 2% galactose, and 1.5% agar, and the screening medium for selection of transformed colonies included 0.67% YNB and 2% of a carbon source (D-glucose or D-fructose), supplemented with the appropriate amino acids lacking uracil. As a negative control, the plasmid without the PeHXK gene was transformed into the yeast mutant cultured on medium containing 0.67% YNB and 2% of a carbon source (D-glucose or D-fructose).

## Results

### Identification and Characterization of Hexokinase Genes in Moso Bamboo

To identify HXK genes in moso bamboo, HXK protein sequences of *Arabidopsis* were used as query sequences to search the moso bamboo genome. A total of 15 candidate sequences were obtained and three sequences were subsequently discarded after analysis of conserved domains (Table 1 and Supplementary File S3). The molecular weight (MW) of PeHXK proteins and CDS length of the PeHXK genes showed substantial variation. The CDS length ranged from 723 bp (PeHXK1) to 1680 bp (PeHXK9). The encoded protein length ranged from 240 amino acids (aa) (PeHXK1) to 559 aa (PeHXK9). The protein molecular weight ranged from 26.18 kDa (PeHXK1) to 60.93 kDa (PeHXK9). More details for the individual PeHXKs, AtHXKs, and OsHXKs, including additional characteristics such as pI, and scaffold location, were given in Table 1.

**TABLE 1:** Detailed information about HXKs in moso bamboo, *Arabidopsis thaliana*, and rice.

			Protein		
Gene name	Chromosome location	CDS length (bp)	Length (aa)	PI	MW (Da)
PeHXK1	hic_scaffold_16:111085203:111086886	723	240	5.21	26180.9
PeHXK2	hic_scaffold_8:40366952:40370618	960	319	7.79	34859.2
PeHXK3a	hic_scaffold_16:4759058:4766546	1503	500	5.94	53983.6
PeHXK3b	hic_scaffold_14:83792767:83799122	1320	439	5.63	47533.2
PeHXK4	hic_scaffold_23:81412193:81415229	1476	491	5.25	52916.0
PeHXK5a	hic_scaffold_9:56624640:56628941	1524	507	5.75	54835.9
PeHXK5b	hic_scaffold_7:41944655:41952156	1524	507	6.05	55003.2
PeHXK6	hic_scaffold_16:36093974:36111483	1317	438	5.29	47853.6
PeHXK7	hic_scaffold_9:11910451:11913117	1395	464	5.00	49662.6
PeHXK8	hic_scaffold_14:96231164:96234062	1407	468	5.23	50994.0
PeHXK9	hic_scaffold_14:56197681:56209895	1680	559	5.43	60927.2
PeHXK10	hic_scaffold_7:17572419:17578952	1299	432	5.25	47044.9
AtHXK1	Chr4:14352037.14355201	1491	496	5.76	53706.9
AtHXK2	Chr2:8570607.8574067	1509	502	5.73	54489.9
AtHXK3	Chr1:17616051.17619011	1482	493	6.35	53879.9
AtHKL1	Chr1:18693644.18697706	1497	498	5.55	54590.7
AtHKL2	Chr3:6994770.6998185	1509	502	8.12	54955.4
AtHKL3	Chr4:17790080.17792198	1482	493	5.72	54241.2
OsHXK1	Chr7:15293156.15294805	1497	498	4.89	51772.0
OsHXK2	Chr5:26418621.26422556	1485	494	5.77	53625.6
OsHXK3	Chr1:41305317.41314527	1503	500	6.07	53794.4
OsHXK4	Chr7:5256320.5259795	1530	509	6.49	54758.7
OsHXK5	Chr5:26017295.26022937	1524	507	5.75	54658.6
OsHXK6	Chr1:31009006.31014001	1521	506	5.92	55120.9
OsHXK7	Chr5:5337195.5341210	1392	463	5.22	49760.9
OsHXK8	Chr1:4820104.4823129	1419	472	5.71	50920.2
OsHXK9	Chr1:30131348.30135287	1509	502	6.38	54496.4
OsHXK10	Chr5:18075301.18081280	1515	504	5.35	54507.2

*CDS, coding sequence; PI, protein isoelectric point; MW, molecular weight of protein.*

### Phylogenetic Relationships and Multiple Alignments

**FIGURE 1:**
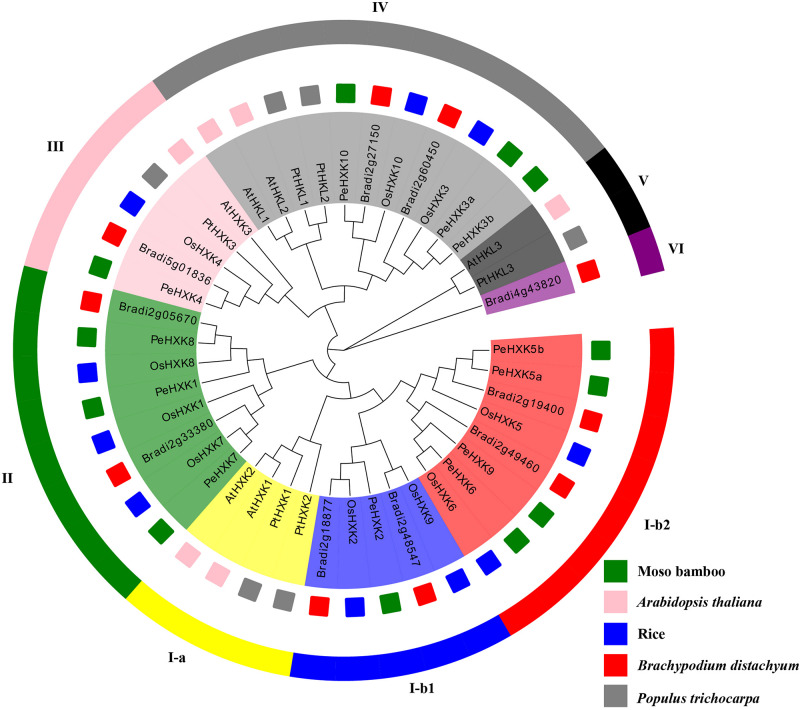
Phylogenetic tree of PeHXK proteins from moso bamboo, *Arabidopsis thaliana*, rice, *Brachypodium distachyon*, and *Populus trichocarpa*. The tree was generated using the Neighbor-Joining (N-J) method with 1000 replicates. The fragments with different colors in the outer ring represent different subfamilies. The small squares with colors inside indicate different species [pink, *A. thaliana*; blue, *O. sativa* (rice); red, *B. distachyon*; green, moso bamboo].

Previous studies have identified HXK genes in many plant species. To explore the evolutionary relationships among PeHXKs and HXKs in other plant species, 44 full-length HXK protein sequences, comprising six protein sequences from *Arabidopsis*, 10 protein sequences from rice, six protein sequences from poplar (*Populus trichocarpa*), 10 protein sequences from *Brachypodium distachyon*, and the 12 protein sequences from moso bamboo were used to construct a phylogenetic tree. The accession numbers of the sequences are listed in Supplementary Table S1. The evolutionary tree was approximately divisible into eight subfamilies, of which PeHXK members were distributed among five subfamilies (Figure 1). On the basis of the phylogenetic tree, no homologous sequences of AtHXK1, AtHXK2, and AtHKL3 were present in monocotyledonous plants. Among the PeHXK proteins, PeHXK8, PeHXK1, and PeHXK7 were represented as most similar to three type C OsHXKs (OsHXK1, OsHXK7, and OsHXK8), which contain neither chloroplast transport peptides nor membrane-anchored domains, and PeHXK4 was clustered with OsHXK4, which contains the type A-specific chloroplast transit peptide (Karve et al., 2010). The remaining PeHXK proteins were grouped with type B OsHXKs.

**FIGURE 2:**
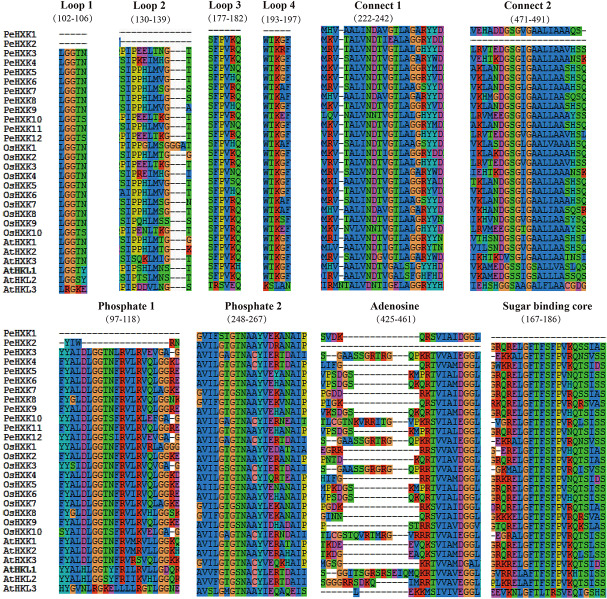
Gene structure of HXKs in moso bamboo, *Arabidopsis thaliana*, and rice. The HXKs were divided into five distinct sub-groups marked as left. It was specially noted that PeHXK6 and PeHXK9 genomic sequences contain much longer introns, while only OsHXK1 has no intron in the genome at all. The exons, introns, and UTR sections are indicated by yellow rectangles, black lines, and blue rectangles, respectively.

For further characterization of the PeHXK proteins, the protein sequences were aligned using SeaView 4 software. Prediction of conserved sequences was based on previous reports of HXK2 in *Saccharomyces cerevisiae* (Bork et al., 1992; Katz et al., 2000; Aguilera-Alvarado et al., 2019). The majority of PeHXK proteins contained two conserved domains: an ATP-binding domain with phosphate 1 (P1), phosphate 2 (P2), and adenosine, and a sugar-binding domain. A number of loops and connects were detected in the protein structure. Exceptionally, both PeHXK1 and PeHXK2 had no complete loops. Moreover, PeHXK1 lacked the P1 (in the ATP-binding domain) and sugar-binding core while PeHXK2 had no connect 2, adenosine, and truncated P1 (Figure 2). A more detailed protein sequence comparison between *Arabidopsis thaliana*, rice, and moso bamboo was shown in Supplementary Figure S1.

### Gene Structure, Chromosomal Location, and Motif Distribution

**FIGURE 3:**
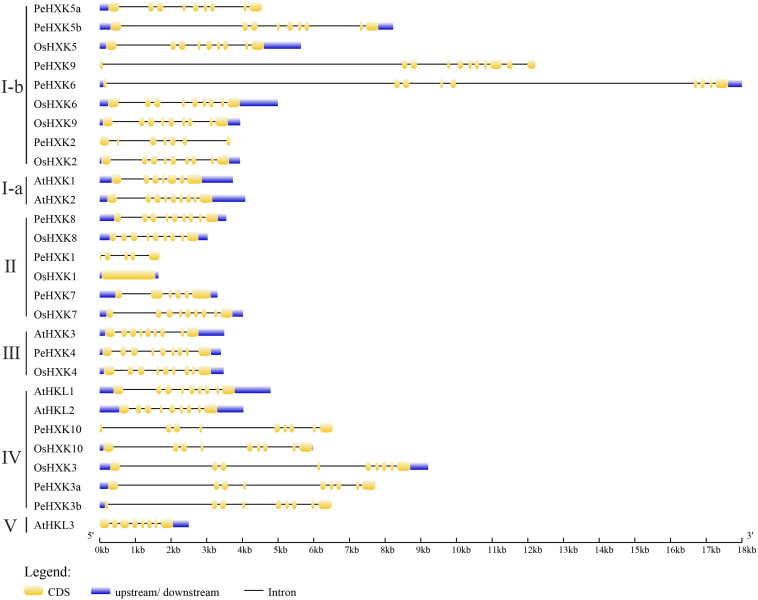
Conserved domain alignment for predicted moso bamboo family proteins. The sequences from moso bamboo, *Arabidopsis thaliana*, and rice were aligned using MUSCLE. Annotations are based on regions homologous to yeast HXK2 (Kuser et al., 2000). P1, phosphate 1; P2, phosphate 2. The alignment was performed using SeaView4 software (Gouy et al., 2010).

On the basis of the genome and CDS sequences of the candidate PeHXKs (Supplementary Files S1, S2), the exon–intron structure was predicted using the GSDS tool. The PeHXKs from different subfamilies showed differences in gene structure. The majority of PeHXK genes contained at least nine exons and eight introns, whereas PeHXK1 contained only five exons and four introns. Five genes (PeHXK5b, PeHXK6, PeHXK7, PeHXK8, and PeHXK4) contained both 5′–and 3′- untranslated regions (UTRs), three genes (PeHXK5a, PeHXK3a, and PeHXK3b) contained a 5′-UTR only, and the remaining four genes (PeHXK9, PeHXK2, PeHXK1, and PeHXK10) contained no UTR region (Figure 3).

To determine the distribution of PeHXK genes in the moso bamboo genome, we mapped the PeHXK genes to individual chromosomes. The PeHXK genes were located on six chromosomes, of which chromosomes 14 and 16 each carried three PeHXK genes, chromosomes 7 and 9 each carried two PeHXK genes, and one PeHXK was located on each of chromosomes 23 and 8 (Table 1 and Supplementary Figure S2).

**FIGURE 4:**
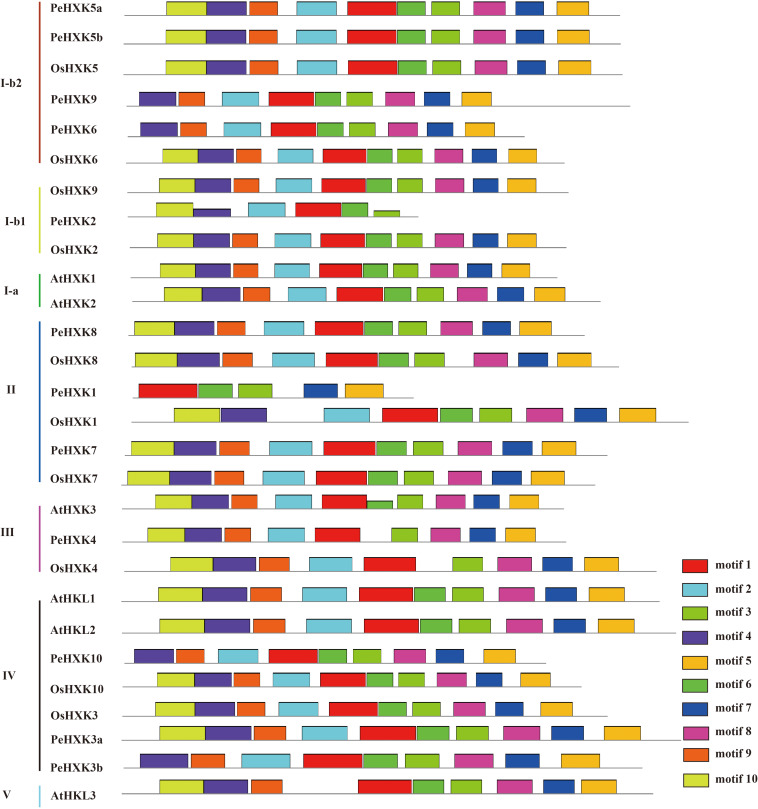
Motif distribution in HXKs from moso bamboo, *Arabidopsis thaliana*, and rice. Motifs were analyzed using the MEME web server (https://www.swissmodel.expasy.org/). The motifs are represented by different colors. The length of each box in the figure does not represent the actual motif size.

To analyze PeHXK protein motif characteristics, we used the MEME tool to predict conserved motifs (Figure 4). In total, 10 conserved motifs in the PeHXK protein sequences were predicted. The majority of PeHXK proteins contained at least nine conserved domains, whereas PeHXK2 and PeHXK1 contained five and four motifs, respectively. It was noteworthy that 10 of the 12 HXK proteins contained motif 1, 3, and 8, which were all components of the ATP-binding domain.

### Evolutionary Patterns Among Moso Bamboo, Rice, and *Brachypodium distachyon*

Selection pressure refers to the evolutionary pressure that nature places on organisms to enable those who adapt to the natural environment to survive and reproduce. To investigate the selection pressure on HXK genes in moso bamboo, rice, and *Brachypodium distachyon*, we identified eight orthologs (Pe-Os) between moso bamboo and rice, and nine orthologs (Pe-Br) between moso bamboo and *Brachypodium distachyon* using bidirectional best-hit methods (Wu et al., 2016). All gene pairs are listed in Table 2. A previous study (Juretic et al., 2005) showed that the Ka/Ks ratio can be applied as a measure of selection pressure. Accordingly, we calculated the values of Ka, Ks, and Ka/Ks. The value of Ka and Ks respectively represent the non-synonymous substitution rate and synonymous substitution rate. The Ka/Ks ratio was widely used to calculate selection pressure. Generally, the value of Ka/Ks greater than 1, equal to 1, and less than 1 respectively represents positive selection, neutral selection, and negative selection (Juretic et al., 2005). The Ka/Ks values for the Pe-Os and Pe-Br orthologous pairs ranged from 0.039 to 0.266 and 0.100 to 0.22, respectively. The Ks values of Pe-Os pairs ranged from 0.26 to 4.1, suggesting that the orthologs diverged 20 million years ago (MYA). The Ks values of the nine Pe-Br pairs ranged from 0.24 and 1.59, indicating that these orthologs diverged prior to 19 MYA.

**TABLE 2:** Divergence between orthologous gene pairs.

Orthologous gene pairs		Ka	Ks	Ka/Ks	MYA
PH02Gene06290.t1 (PeHXK8)	Bradi2g05670	0.0649	0.2962	0.219134	22.78
PH02Gene24831.t1 (PeHXK5a)	Bradi2g18877	0.2229	1.5228	0.146369	117.14
PH02Gene24831.t1 (PeHXK5a)	Bradi2g19400	0.0405	0.2752	0.147312	22.17
PH02Gene40020.t1 (PeHXK10)	Bradi2g27150	0.0935	0.4700	0.198977	36.16
PH02Gene45438.t1 (PeHXK7)	Bradi2g33380	0.0679	0.6795	0.100050	52.27
PH02Gene08025.t1 (PeHXK5b)	Bradi2g48547	0.2667	1.5856	0.168200	121.97
PH02Gene08025.t1 (PeHXK5b)	Bradi2g49460	0.1037	0.6801	0.152489	52.31
PH02Gene41019.t1 (PeHXK3a)	Bradi2g60450	0.0551	0.2491	0.221482	19.16
PH02Gene31153.t1 (PeHXK4)	Bradi5g01836	0.0912	0.4516	0.202087	34.74
PH02Gene40020.t1 (PeHXK10)	OsHXK10	0.0938	0.4359	0.215263	33.53
PH02Gene45438.t1 (PeHXK7)	OsHXK1	0.1626	4.1383	0.039314	318.33
PH02Gene41019.t1 (PeHXK3a)	OsHXK3	0.0400	0.2658	0.150463	20.45
PH02Gene31153.t1 (PeHXK4)	OsHXK4	0.0993	0.4527	0.219425	34.82
PH02Gene24831.t1 (PeHXK5a)	OsHXK5	0.0607	0.2864	0.211980	22.03
PH02Gene24831.t1 (PeHXK5a)	OsHXK6	0.0808	0.7090	0.114013	54.54
PH02Gene45438.t1 (PeHXK7)	OsHXK7	0.0584	0.6593	0.088723	50.71
PH02Gene08025.t1 (PeHXK5b)	OsHXK9	0.2961	1.1117	0.266386	85.52

*Ka, non-synonymous substitution rate; Ks, synonymous substitution rate; MYA, million years ago.*

### Expression Profiles of Moso Bamboo HXK Family Genes

**FIGURE 5:**
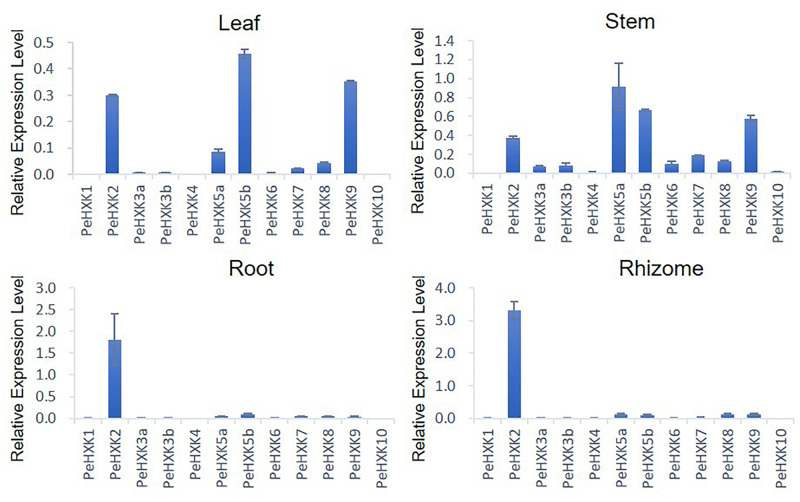
Expression patterns of 12 PeHXK genes in four different tissues. Samples were collected from the leaf, stem, root, and rhizome. The Y-axis indicates relative expression levels of PeHXK genes to that of *TIP41*, which is used as an inner control (Fan et al., 2013).

To explore the tissue-specific expression patterns of PeHXK genes, their transcript levels were measured by qRT-PCR in four tissues of moso bamboo seedlings, namely the leaf, stem, root, and rhizome. Five genes (PeHXK5a, PeHXK8, PeHXK5b, PeHXK9, and PeHXK2) were extensively expressed in the tissues, whereas three genes (PeHXK1, PeHXK4, and PeHXK10) were detected at low levels in all four tissues. PeHXK6, PeHXK3a, and PeHXK3b showed a relatively higher transcript level in the stem than in leaf, rhizome, and root. Except for PeHXK1, PeHXK4, and PeHXK10, the remaining PeHXK genes were actively expressed in the stem. Notably, the level of PeHXK2 transcripts was considerably higher in the rhizome (Figure 5).

### Subcellular Localization of Bamboo HXK Family Genes

**FIGURE 6:**
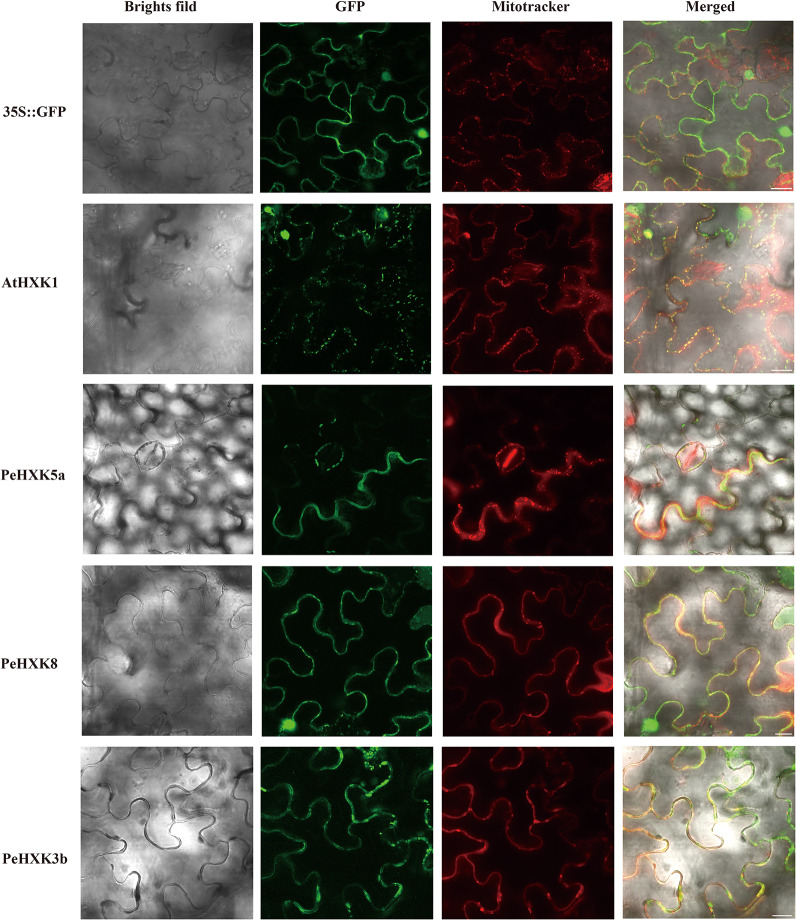
Subcellular location of the PeHXK5a-GFP, PeHXK8-GFP, and PeHXK3b-GFP fusion proteins in tobacco leaves. AtHKX1-GFP and empty vector (pCambia2300GFP) are used as the positive and negative control, respectively. The fluorescence signals were collected by confocal scanning microscopy before pretreatment of dyeing by Mitotracker (red). The merged image was a superposition signals of bright field, GFP and mitotracker signal. Bar = 20 μm.

To determine the subcellular localization of PeHXK proteins, the CDS of three PeHXKs (PeHXK5a, PeHXK8, and PeHXK3b) belonging to different subgroups were fused to the GFP sequence and was transiently expressed in tobacco leaf epidermal cells. The subcellular localization of AtHXK1 and empty plasmid were separately used as a positive and negative control. The fluorescence signals for PeHXK5a-GFP, PeHXK8-GFP, and PeHXK3b-GFP were co-localized with the MitoTracker^®^ Red signal. In addition, PeHXK8-GFP signal was also detected in the nucleus (Figure 6).

### Complementation of Yeast Mutant

**FIGURE 7:**
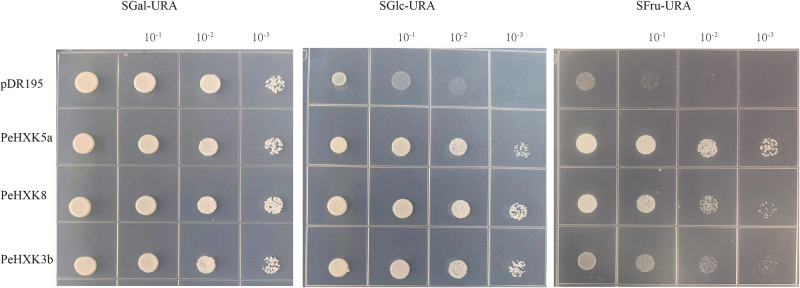
Complementation of the yeast mutant YSH7.4-3C with PeHXK genes. pDR195: the empty pDR195 vector was transformed into YSH7.4-3C as negative control; PeHXK3b, PeHXK5a, and PeHXK8: the recombinant vectors (including the three PeHXK CDS) were transformed into YSH7.4-3C, respectively. SGal-URA, the medium that contained galactose as the sole carbon source and lacked uracil; SGlc-URA, the medium that contained glucose as the sole carbon source and lacked uracil; SFru-URA, the medium that contained fructose as the sole carbon source and lacked uracil.

To evaluate the HXK activity of the PeHXKs, we cloned the full-length CDS of PeHXK5a, PeHXK8, and PeHXK3b into the pDR195 yeast expression vector. The yeast triple-mutant strain YSH7.4-3C was transformed with the recombinant vectors and cultured on screening media. Medium in which galactose was the sole carbon source (SGal-URA) was used as a control. The yeast transformed with the empty plasmid, or with plasmids harboring the PeHXK genes (PeHXK5a, PeHXK8, or PeHXK3b) grew normally on Sgal-URA medium. However, on medium in which glucose was the sole source of carbon (SGlu-URA), only yeasts transformed with PeHXK5a, PeHXK8, and PeHXK3b could grow. On medium that contained fructose as the sole carbon source (SFru-URA), yeast transformed with the three PeHXK genes grew better than the control (Figure 7).

## Discussion

### Identification and Characterization of PeHXK Genes

In addition to acting as signaling molecules, hexose sugars are the main carbon source required for the energy supply and storage to fuel and maintain cell life. Moreover, they are an important source for the synthesis of polysaccharide in the plant cell wall. Before being utilized, hexoses need to be phosphorylated by hexose phosphorylating enzymes, including HXKs (Granot et al., 2013). In many plant species, HXKs have been identified as hexokinases phosphorylating glucose and fructose with diversified functions-sensing sugar, controlling gene expression, hormonal interactions and finally regulating plant growth (Granot et al., 2013; Aguilera-Alvarado and Sanchez-Nieto, 2017). However, no information was previously available on members of the HXK gene family in the Bambusoideae, a distinctive subfamily of the Gramineae. As a representative bamboo species, fast-growth moso bamboo, which is distributed widely in China and the world, is an important source of non-timber forest products in human traditional life and thus is of substantial economic and environmental value (Peng et al., 2013; Zhao et al., 2018). Furthermore, it may be considered as a good source of carbohydrate for biofuel production for its environmental benefits and higher annual biomass yield (He et al., 2014). To accelerate the research of bamboo HXKs function, especially regard to the potential of growth-regulation and biomass-increase, here we carried out a genome-wide analysis of HXK genes in moso bamboo. In the present work, 12 HXKs were identified in the moso bamboo genome and characterized with regard to gene and protein structure, conserved motifs, gene evolution, expression pattern, protein subcellular localization, and HXK enzyme activity. A comparison of HXK gene families among multiple plant species revealed an inconsistency in the numbers of PeHXK genes and the genome size of moso bamboo (Figures 3, 4). For example, *Arabidopsis*, which has an extremely small genome size (125 Mb), contains three HXK genes and three HXK-Like genes, the gramineous plant rice with a genome size of 466 Mb has 10 HXK genes, whereas moso bamboo with a huge genome size of 2.075 Gb contains 12 HXK genes. Apparently, gene duplication events in moso bamboo did not promote an increase in the number of PeHXKs, which indicates that their functions are highly conserved in the species.

High similarity in the protein sequence is commonly associated with function conservation. According to the well-studied tertiary structure of HXKs from yeast and mammals, HXK proteins contain a large and a small domain which is resided largely by the sugar-binding site/core with four additional peptide segments (Loops 1–4) (Bork et al., 1992; Kuser et al., 2000). It also indicates that most of the conserved amino acid residues exist at the fissure of the two domains and generates the glucose and ATP binding sites (Kuser et al., 2000). In our present work, multiple alignments of amino acid sequences revealed that the majority of HXK homologous proteins of bamboo contained complete conserved regions similar to the reported HXKs, including motifs designated phosphate 1, connect 1, phosphate 2, adenosine, and connect 2 (Karve et al., 2010). Exceptions were observed in PeHXK1 and PeHXK2. PeHXK1 lacks L1-L4 loops, and phosphate 1 (P1), and PeHXK2 has no L1-L2 loops, connect 2, and adenosine. Otherwise, PeHXK2 also possesses a big-scale deletion in the P1 motif (Figure 2 and Supplementary Figure S1). For the reasons that the P1 domain is high conserved and required for HXK activity and the loops are the essential component of one of the HXK major domains responsible for glucose binding (Kuser et al., 2000; Karve et al., 2010), the HXK activity consequently would be dismissed in the two HXK proteins in all probability. From a functional point of view, plant HXK family members are classifiable into HXK and HXK-like (HKL) proteins based on whether the protein having the activity to phosphorylate glucose (Moore et al., 2003; Karve et al., 2008; Karve and Moore, 2009). Factually, the functional diversification is considerable in plants. Taking *Arabidopsis* HXK proteins as an example, both AtHXK1 and AtHXK2 can phosphorylate glucose, sense sugar level, and interact with signaling pathways; AtHKL1, AtHKL2, and AtHKL3 are thought to have just regulatory activity like antagonizing AtHXK1/2; and AtHXK3, as a plastid enzyme, is likely only a catalytic protein (Jang et al., 1997; Granot, 2008; Karve et al., 2010). Similar functional diversification also seems to occur in the rice HXK family (Yu and Chiang, 2008; Cho et al., 2009), thus it is possible that HXK genes in rice and other monocot plants are classified into the two groups. By inspection, we classified the 12 PeHXK genes into four groups (Figure 1). In the Group I-b2, PeHXK5a, PeHXK5b, PeHXK6, and PeHXK9 are located together with OsHXK5 and OsHXK6, while in the Group I-b1, only PeHXK2 shares the same branch with OsHXK2 and OsHXK9; In Group II, the branch possesses OsHXK7, OsHXK8 and their homologous genes PeHXK7, PeHXK8, and PeHXK1; In Group III, PeHXK4 is together with OsHXK4 and AtHXK3; In Group IV, PeHXK10, PeHXK3a, and PeHXK3b are clustered with OsHXK10 and OsHXK3. According to the previous study (Karve et al., 2010; Aguilera-Alvarado and Sanchez-Nieto, 2017), we speculated that PeHXK1, PeHXK2, PeHXK5a, PeHXK5b, PeHXK6, PeHXK9, PeHXK7, and PeHXK8 belong to HXK-protein clade, and PeHXK3a, PeHXK3b, PeHXK10 are HKL proteins. PeHXK4 in Group IV is putative plastid-localization like AtHXK3 and OsHXK4. It is noted that the loss of key motifs in PeHXK1 and PeHXK2 might lead to lost of either catalytic or regulatory function as HXK genes. Furthermore, in regard to the homologous relationships with OsHXK5, OsHXK6, and AtHXK1, we also infer that PeHXK5a, PeHXK5b, and PeHXK6 are candidate glucose sensor in moso bamboo.

### Evolutionary Patterns Among HXK Genes of Moso Bamboo, Rice, and *Brachypodium distachyon*

Duplication events are an important source of novelty in genome evolution. The new gene copies that result from replication may lead to families of genes that evolve novel functions (Moore and Purugganan, 2003). Taking HXK genes as a reference, we estimated that a large-scale duplication event occurred approximately 22–121 and 20–319 MYA on the basis of the estimated divergence times for orthologous gene pairs (Pe-Os and Pe-Br) (Table 2). It was previously estimated that PHD-finger gene families diverged approximately 19–55 and 22–60 MYA between moso bamboo and rice, and between moso bamboo and *Brachypodium distachyon*, respectively (Liu et al., 2018). These results indicate that different gene families may have been duplicated at different evolutionary time points.

### Expression Patterns, Subcellular Localization, and HXK Activity

The expression level of proteins in different tissues and information on subcellular localization of proteins provides a foundation to understand their function in plant growth and development. In *Arabidopsis*, most HXK genes are expressed extensively in all tissues, while *AtHKL3* is only detected in flower (Karve et al., 2008). In rice, the OsHXK family shows the similarly wide range of expression profiles with the exception that *OsHXK10* is only actively transcribed in flower and *OsHXK1* is not detected in any tissue (Cho et al., 2006). With the exception of *AtHKL3*, *OsHXK1*, and *OsHXK10*, transcripts of all HXK genes in *Arabidopsis* and rice were detectable in main tissues, suggesting that each plant HXK has either a unique or redundant function in various tissues or organs. In the present study, the expression patterns of PeHXK genes were examined in the leaf, stem, rhizome, and root of moso bamboo seedlings and the results showed that PeHXK family genes have some different profiles (Figure 5). For example, not like *AtHXK* or *OsHXK* genes, most PeHXK family members showed a very low expression level in root and rhizome. However, some similar patterns to rice are also observed in PeHXK gene expression. As an example, *PeHXK1* and *PeHXK10* were almost not detectable in all four tissues tested, like *OsHXK1* and *OsHXK10*, respectively. Moreover, *PeHXK5a, PeHXK5b, PeHXK9* showed relatively higher expression levels in leaf and stem, just like their homologous ones in rice, *OsHXK5*, *OsHXK6*, and *OsHXK9*, respectively. Additionally, what is interesting is that the expression levels of *PeHXK2*, which is mentioned above as inactive with incomplete HXK motifs, showed high transcriptional activity in all four tissues detected, especially in root and rhizome, in both which *PeHXK2* is the only one showing high expression, indicating that the *PeHXK2* might perform a unique function like as a negative regulator for the other HXKs (Karve and Moore, 2009; Aguilera-Alvarado and Sanchez-Nieto, 2017).

Plants have four HXK types, types A–D, based on their subcellular localization, and differences in subcellular localization result in functional divergence (Karve et al., 2010; Aguilera-Alvarado and Sanchez-Nieto, 2017). To date, except for type D HXKs, which are mitochondrial proteins, have only been identified in the moss *Physcomitrella patens* (Nilsson et al., 2011), the other three types of HXKs are widely distributed in higher plants. Type A HXKs harboring a chloroplast signal at N-terminus commonly localized at chloroplasts and have been found in moss *Physcomitrella patens* and higher plants such as *Arabidopsis thaliana*, *Nicotina tabacum*, *Oryza sativa*, *Solanum lycopersicum, Vitis vinifera*, *Camellia sinensis*, *Brassica napus*, pear (*Pyrus* × *bretschneideri*), and *Spinacia oleracea* (Olsson et al., 2003; Cho et al., 2006; Kandel-Kfir et al., 2006; Karve et al., 2008, 2010; Nilsson et al., 2011; Wang et al., 2018; Zhao et al., 2019). Type B HXKs, accounting for most members of HXK family in plants, have a highly hydrophobic helix and associates with mitochondria. Also, some type B HXKs with a nuclear translocation sequence adjacent to the membrane anchor domain can be translocated to the nucleus (Heazlewood et al., 2004; Cho et al., 2006, 2009; Camacho-Pereira et al., 2009; Cheng et al., 2011). Type C HXKs have no signal peptides or membrane anchors and are thought to be cytosolic proteins. They seem to be only present in moss and monocotyledonous plants such as rice and maize (Karve et al., 2010; Cheng et al., 2011; Nilsson et al., 2011). In the present work, we identified one type A PeHXK (PeHXK4), eight type B PeHXKs (PeHXK2, PeHXK3a, PeHXK3b, PeHXK5a, PeHXK5b, PeHXK6, PeHXK9, PeHXK10), and three type C HXKs (PeHXK1, PeHXK7, PeHXK8) based on phylogenetic analysis. To confirm previous reports about the localization of different types of PeHXK proteins, we originally planned to choose three PeHXKs from corresponding three types of PeHXKs. But we failed because the PeHXK4 was not cloned successfully. Thus, finally, we chose PeHXK3b, PeHXK5a, and PeHXK8 as targets to analyze PeHXK protein localization (Figure 6). Our results showed that PeHXK3b and PeHXK5a localized at mitochondria of tobacco leaf epidermal cells, in consistence with that of OsHXK5 and OsHXK6 (Cho et al., 2009). Unlike cytoplasmic protein OsHXK7 which shares the same branch in the phylogenetic tree (Cho et al., 2006), PeHXK8 was detected in both mitochondria and nucleus of leaf epidermal cells. These results suggest that PeHXK3b and PeHXK5a are relatively conserved in their subcellular localization; However, differences in the subcellular localization of PeHXK8 is suggestive of functional variation.

The activity of HXK can be readily determined by means of a yeast complementation assay *in vitro* (Geng et al., 2017). In the current research, we cloned three PeHXK genes and verified that the three PeHXKs showed HXK activity (Figure 7). It indicated that PeHXK5a and PeHXK8 had an equal ability for phosphorylating glucose and fructose, while PeHXK3b showed favor for glucose as the carbon source to phosphorylate. These results confirmed the two traits of plant HXKs: their low selectivity for substrates, and their preference for glucose over fructose (Karve et al., 2010; Aguilera-Alvarado and Sanchez-Nieto, 2017).

## Conclusion

In this study, 12 HXK family genes in moso bamboo were identified and characterized by analysis of phylogenetic relationships, protein and gene structure, structural domains, and estimation of divergence times in evolutionary history. Expression profile analysis implied that these genes were expressed extensively in moso bamboo tissues and may play pivotal roles in plant growth and development. The localization analysis showed that PeHXK3b and PeHX5a were associated with mitochondria while PeHXK8 was localized to both mitochondria and nucleus. An HXK activity assay using the yeast triple-mutant strain YSH7.4-3C verified that the three PeHXKs showed HXK activity with the plant HXK-specific enzyme traits. The present work lays a foundation for further investigation of HXKs in moso bamboo and would accelerate the future cloning and functional analyses of PeHXK genes in moso bamboo.

## Data Availability Statement

The datasets generated for this study are available on request to the corresponding author.

## Author Contributions

LD, SC, and CL conceived and designed the experiments. WZ, YZ, QZ, RW, XW, and SF performed the experiments and interpreted the data. WZ and LD drafted and revised the manuscript.

## Conflict of Interest

The authors declare that the research was conducted in the absence of any commercial or financial relationships that could be construed as a potential conflict of interest.

## Footnotes

1 https://www.arabidopsis.org/

2 ftp://parrot.genomics.cn/gigadb/pub/10.5524/100001_101000/100498/

3 https://www.ncbi.nlm.nih.gov/Structure/cdd/wrpsb.cgi

4 http://pfam.xfam.org/search#tabview=tab1

5 https://web.expasy.org/protparam/

6 http://gsds.cbi.pku.edu.cn/

7 http://meme-suite.org/tools/meme

8 https://www.ncbi.nlm.nih.gov/

9 https://phytozome.jgi.doe.gov/pz/portal.html

10 https://www.ncbi.nlm.nih.gov/tools/primer-blast/index.cgi?LINK_LOC=BlastHome

## Supplementary Material

FIGURE S1 Amino acids alignment of proteins form moso bamboo, *Arabidopsis thaliana*, and rice.

FIGURE S2 The chromosomal distribution of PeHXK genes.

TABLE S1 List of genes used in this study.

TABLE S2 Primers used in this study.

FILE S1 Genomic sequences of PeHXK genes.

FILE S2 CDS of PeHXK genes.

FILE S3 Protein sequences of PeHXKs.
