# Supplementary figures and images for: Genome-Wide Identification and Characterization of Hexokinase Genes in Moso Bamboo (Phyllostachys edulis)
Source: Front Plant Sci. 2020 May 19;11:600. doi: 10.3389/fpls.2020.00600 (PMC7248402; doi:10.3389/fpls.2020.00600)

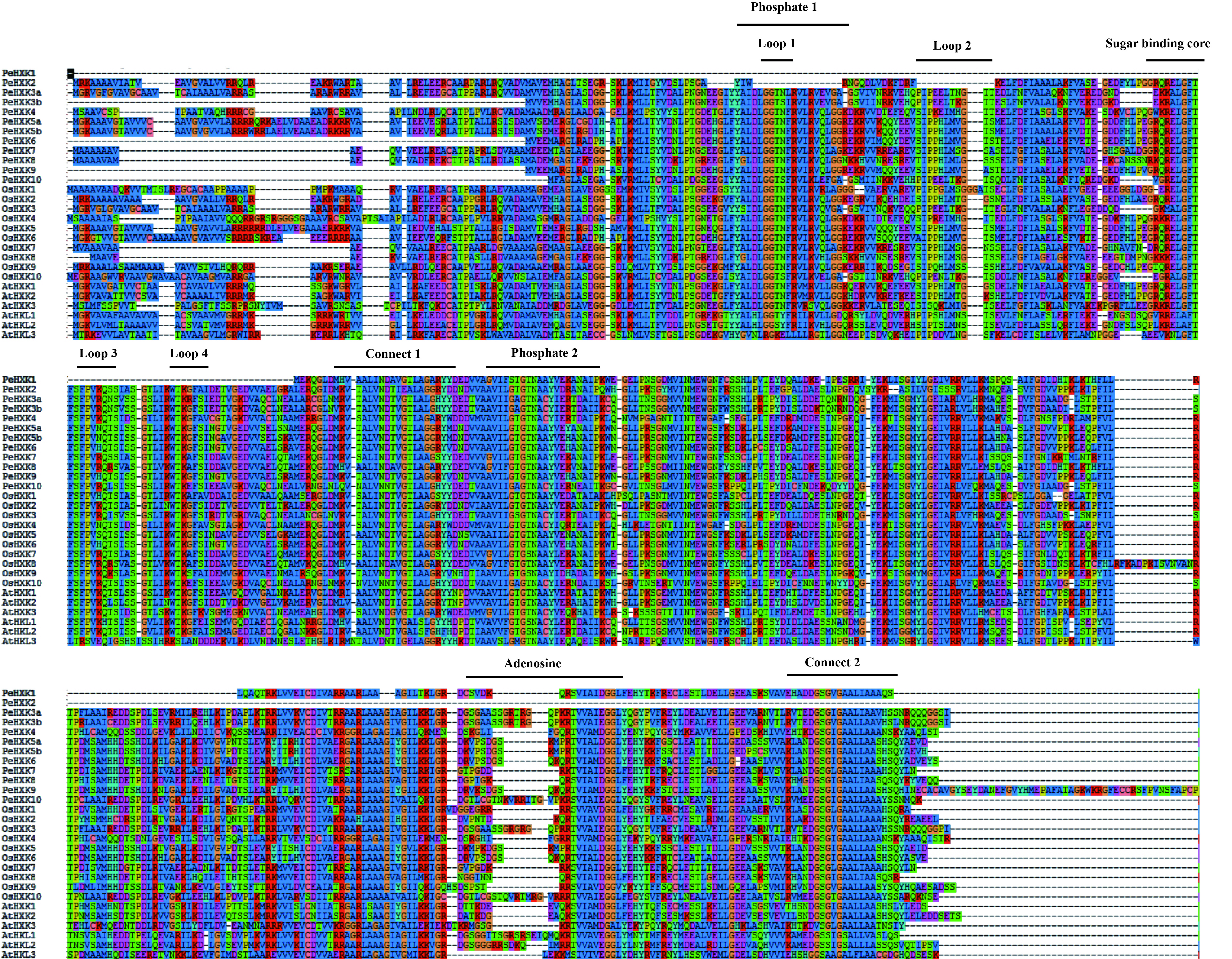

Supplement: FIGURE S1 — Amino acids alignment of proteins form moso bamboo, Arabidopsis thaliana, and rice. [file Image_1.JPEG]

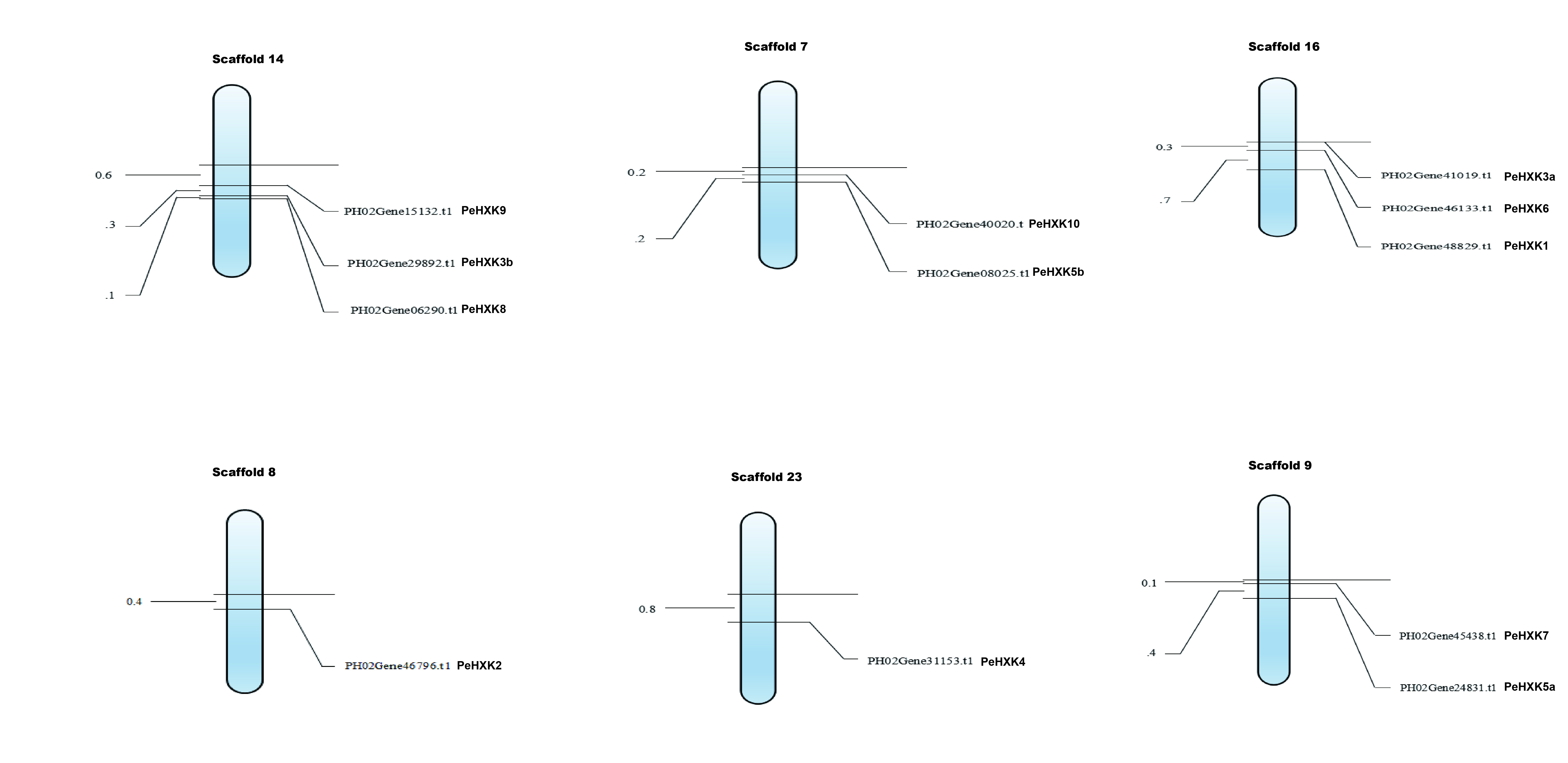

Supplement: FIGURE S2 — The chromosomal distribution of PeHXK genes. [file Image_2.JPEG]
