# Supplementary material for: Genome-Wide Identification and Characterization of Hexokinase Genes in Moso Bamboo (Phyllostachys edulis)
Source: Front Plant Sci. 2020 May 19;11:600. doi: 10.3389/fpls.2020.00600 (PMC7248402; doi:10.3389/fpls.2020.00600)
Supplement: TABLE S1 — List of genes used in this study. [file Table_1.docx]

**Table S1.** List of genes used in this study*.*

| Gene Name | Gene ID | Gene Name | Gene ID |
| --- | --- | --- | --- |
| *AtHXK1* | At4g29130 | *OsHXK1* | LOC_Os07g26540 |
| *AtHXK2* | At2g19860 | *OsHXK2* | LOC_Os05g45590 |
| *AtHXK3* | At1g47840 | *OsHXK3* | LOC_Os01g71320 |
| *AtHKL1* | At1g50460 | *OsHXK4* | LOC_Os07g09890 |
| *AtHKL2* | At3g20040 | *OsHXK5* | LOC_Os05g44760 |
| *AtHKL3* | At4g37840 | *OsHXK6* | LOC_Os01g53930 |
| *OsHXK7* | LOC_Os05g09500 | Bradi2g18877 | Bradi2g18877.1 |
| *OsHXK8* | LOC_Os01g09460 | Bradi2g19400 | Bradi2g19400.4 |
| *OsHXK9* | LOC_Os01g52450 | Bradi2g49460 | Bradi2g49460.1 |
| *OsHXK10* | LOC_Os05g31110 | Bradi2g48547 | Bradi2g48547.1 |
| Bradi2g33380 | Bradi2g33380.1 | *PtHXK1* | Potri.018G088300.1 |
| Bradi2g05670 | Bradi2g05670.2 | *PtHXK2* | Potri.001G190400.1 |
| Bradi5g01836 | Bradi5g01836.1 | *PtHXK3* | Potri.005G238600.1 |
| Bradi2g60450 | Bradi2g60450.1 | *PtHKL1* | Potri.009G050000.1 |
| Bradi2g27150 | Bradi2g27150.1 | *PtHKL2* | Potri.001G254800.1 |
| Bradi4g43820 | Bradi4g43820.1 | *PtHKL3* | Potri.007G009300.1 |
| *PeHXK1* | PH02Gene48829.t1 | *PeHXK2* | PH02Gene46796.t1 |
| *PeHXK3a* | PH02Gene41019.t1 | *PeHXK3b* | PH02Gene29892.t1 |
| *PeHXK4* | PH02Gene31153.t1 | *PeHXK5a* | PH02Gene24831.t1 |
| *PeHXK5b* | PH02Gene08025.t1 | *PeHXK6* | PH02Gene46133.t1 |
| *PeHXK7* | PH02Gene45438.t1 | *PeHXK8* | PH02Gene06290.t1 |
| *PeHXK9* | PH02Gene15132.t1 | *PeHXK10* | PH02Gene40020.t1 |
