# Supplementary material for: Genome-Wide Identification and Characterization of Hexokinase Genes in Moso Bamboo (Phyllostachys edulis)
Source: Front Plant Sci. 2020 May 19;11:600. doi: 10.3389/fpls.2020.00600 (PMC7248402; doi:10.3389/fpls.2020.00600)
Supplement: TABLE S2 — Primers used in this study. [file Table_2.docx]

Table S2. Primers used in this study.

| *PeHXK5a* (PCR) | Forward primers | CAGACAGTGATGGGGAAGGC |
| --- | --- | --- |
|  | Reverse primers | GTCGACCTCGGCATACTGAGAGTGC |
| *PeHXK8* (PCR) | Forward primers | ATGGCCGCAGCTGCGGTCGCAAT |
|  | Reverse primers | CTGTTGCTCAACATACTTGTACTG |
| *PeHXK3b* (PCR) | Forward primers | ATGGTCGTTGAGATGCACGC |
|  | Reverse primers | TATGGAACCTCCTTGTTGCTGTCTA |
| *PeHXK1* (qRT) | Forward primers | ACACCAAATTCCGCGAATGC |
|  | Reverse primers | AACTCCTGAACCGTCGTCTG |
| *PeHXK2* (qRT) | Forward primers | ATTGGCTGGTGGGAGATACG |
|  | Reverse primers | CCTGAAGTTCCCCCATTCCA |
| *PeHXK3a* (qRT) | Forward primers | GCAATTCGCGAGGACGATTC |
|  | Reverse primers | CAGTATCCCCGCTATGCCAG |
| *PeHXK3b* (qRT) | Forward primers | GCGAGACTGGTGCTCCATAG |
|  | Reverse primers | TGGTGAATCGTCCTCGCAAA |
| *PeHXK4* (qRT) | Forward primers | TCCTTTCCGGTGAAGCAGAC |
|  | Reverse primers | ACGTGCTCCAGCTAAGGTTC |
| *PeHXK5a* (qRT) | Forward primers | CTTTTCCATCAACGGCACGG |
|  | Reverse primers | ATCTCCCACCAGCCAATGTG |
| *PeHXK6* (qRT) | Forward primers | AAGCTACGTCGACAACCTCC |
|  | Reverse primers | CCACCAAGCTGAACCCGTAT |
| *PeHXK7* (qRT) | Forward primers | TGATCAATGACACCGTCGGG |
|  | Reverse primers | GGTAGCTCGCCTTCCAACTT |
| *PeHXK8* (qRT) | Forward primers | ACACCAAATTCCGCGAATGC |
|  | Reverse primers | AGCCCCTATCCCTGAACCAT |
| *PeHXK9* (qRT) | Forward primers | AGAAGCTGGGCAGGGATAGA |
|  | Reverse primers | GCAGGTCTGCAAGAGTCGAT |
| *PeHXK10* (qRT) | Forward primers | AAGAATAACAGGCGTGCCGA |
|  | Reverse primers | AAGACTAACAGTGGCCGCAA |
| *TIP41* | Forward primers | AAAATCATTGTAGGCCATTGTCG |
|  | Reverse primers | ACTAAATTAAGCCAGCGGGAGTG |
