# Supplementary material for: Genome-Wide Identification and Characterization of Hexokinase Genes in Moso Bamboo (Phyllostachys edulis)
Source: Front Plant Sci. 2020 May 19;11:600. doi: 10.3389/fpls.2020.00600 (PMC7248402; doi:10.3389/fpls.2020.00600)
Supplement: FILE S1 — Genomic sequences of PeHXK genes. [file Data_Sheet_1.docx]

PeHXK genomic sequences

>PeHXK1

ATGGAGAAGCAAGGTCTGGACATGCATGTGGCTGCATTGGTGAGTGTTGCCAGGTCTGCATACCGTCATTGTTCCTAAATAAAGACAACCTATTCGTAATCTTAGAAGAAGAATCTCACTCTGAGAGATCTTTTAATGAAATAGATTAATGATGCTGTTGGGACGTTGGCTGGAGCCAGATACTATGATGAAGATGTTGTCGCAGGCGTGATATTTAGTACTGGCACAAATGCCGCATATGTTGAGAAGGCAAATGCTATACCGAAATGGGAAGGAGAGTTGCCCAATTCAGGGGATATGGTAAGGTTTAAAGGCCATACTTCTATTATACTACCAAAGTAGAGGAAAAATAAGCTGCCATGTTCGCTCTCAAGGCCTATAAATTACGGAGAGAAAAAAAGATCCACACCGTCAACTGTAGTAGGTTCATATTTTAACGGTGCAAATTAATCGGGTGTATATGGGAGCTGTTAAAGTAGAATGAAAAATAAAATTGTATTTTCATTCAATATATAAGTATTGGAGTTGGTTAACAAATACAATGCTATTTTTAGAGGCATTGTAATAGCTAGCAAGAATTCGCTAGTTTCACTTAAAACACAGTGGTACTCAAGCAGCACCTATGGTTTGGATTCGGATGAAGTATTTTATTGTATTTCTAAATAATTTCTTTAATGCTTTTCCTGTATTTCAGGTCATCAATATGGAATGGGGTAACTTCTGCTCATCCCATCTTCCAGTCACTGAATACGATCAAGCATTAGATAAGGAAATCCCGGAGAGCAGGCGAGTTTCTCCACAGAAGACATCATAGTCTTGACTTCCACACTGCTGGCTCACTGATGTACTTTTGTCTTCTTTCAGATCTACGAGAAGTTAATATCGGGAATTTATTTAGGTGAAATAGTCAGGAGGGTGCTGCTTAAAATGTCCCCGCAATCTGCAATTTTCGGCGATATTGATCACACTAAGCTCAAAACTCATTTCCTTCTGCGGTATGTGGTTTGCTTACCTTTTTGCAGTAGGCGTTCTAATGTGACGGTATAATGTTAATCATCAGCAATTCAGGATTTGAAAGGAAAGGTTGCTTTCAAAATTCAAATCCTATCGTGTTATGAAAATAAAATGCATGCTTCTATTCTATTGACGTTCAACAGATTGTTTCGTTAAGTGCGCTGATAAATCTCGGTCTATGTGGTATCCAAGACTCTGACACATTTCTGCAATGCACCATGATGAAACACCTGATCTGAAGATTGTGGCTGAAAAACTGGAAGAAAACCTAGAGGTAGGCTACCGCTTTGACCATTGCTCAGCAGTATTTTATCCATGCGCATCCTTGTGTATGTATGCACTGTAGTGACATGATATGCGTGCTTGCAGATTACAGGCACAGACAAGAAAATTGGTTGTGGAAATCTGTGACATTGTAGCAAGAAGGGCAGCGCGGCTGGCCGCTGCAGGAATCCTCACGAAGCTTGGGAGAGATTGCTCCGTCGACAAGCAGCGGTCAGTCATCGCCATCGATGGCGGACTGTTCGAACACTACACCAAATTCCGCGAATGCTTGGAAAGCACACTGGATGAGCTGCTGGGAGAGGAGGCGTCGAAGTCAGTAGCCGTCGAGCACGCAGACGACGGTTCAGGAGTTGGGGCAGCTCTGATTGCTGCTGCTCAATCTTAA

>PeHXK2

ATGAGGAAGGCAGCGGCGGCGGTTATCGCGACAGTGGAGGCGGTCGGCGTGGCGCTAGTGGTACGGCGGCAGCTGCGGGAGGCGAAGAGGTGGGCGCGCACCGCGGCGGTGCTGCGGGAGCTGGAGGAGCGGTGCGCGGCACGGCCAGCGCGGCTGCGGCAGGTGGCGGATGTGATGGCCGTCGAGATGCATGCGGGGCTCACGTCAGAGGGCAGGAGTAAACTCAAGATGATCATCGGCTACGTCGACTCCCTCCCGTCCGGGTAGTAAGCTCAAGATGATCATCAGCTACGTCGACTCCCTCCCGTCCGGGTAAAACAATCGCCTCTTCTCGCGCAATGTTCTGATTTGGATGGGAGGTGTAGGGGTGCTTTGCTTACGTGATTCGTTGTTCGAGAATGGCTGCTTGGAGGGAGCAATGTGTGGTCCAACAGATCCGTGTTTGAGTTAAAAGTGGGATGTTTGTTTGGTGCTAGTGTCGCTGTCTAGTGCCTACATTTGGAGAAATGGGCAAGATCTTGTTGATAAATTTGACCGTTTTAAGGTGCCGTCCATACTGCTATGATGGTTTGGTCTAAAAGATGTGAACTTGATGCAACAAATCACTATCAATATTATGCCATTTCGGTGAGAGATAAAGATCGGCACTCGCCAATTCGCCATGATTTCACTATGAATTGACTGGTTGAACCAACATTGAGCATATGGTTGTTGCAAGATGAAGATTATTGTTTTTTAGTTGAAAGGATTTAAATGTAGAGGAGCTAGTCGTTATGTGCCTTACATAGAAAACCAACCTTACTAACCATATCTAAAACCCTTTAAGTCCATTGGTGCGAAAGCCAACTGTTATGCACTTTTATATTGTCTCTTCTGTGGTTACCTGTAACCATGTAATTATTGTGAGTCACTGGAACACATATATACTAGGTATGCTTTTGATATTTGTTAGACATTTTAGATGATACAAAGGGCGTCAAAGTAATAATTGAACTAACAAGTAAAATAATTTAACTATTGTGATGGGTATATTGTGCTAACAAGTACCACAGATTGAATTTGAACTTATTGCCGAGGTGCGGCCTCCAGCAAATAATTGAACTATTGCATGCAGTTGCTCTTTGGATTGATAAATCATAAATTTGATATGATGAAGGCTGCATTCACAGGGAAGAGAAAGGGCTATTTTATGCACTTGACCTTGGAGGAACAAATTTCCGTGTTTTACGTGTTCAATTAGGAGGCAATGAAGGGTGAGTTGTCAAGCAAGAATATGAAGGGATTTCAATTCCGCCACATTTAATGACTGGGAGTTCACATGTAAAAATTGCCCCCTTTGTCTATTTTGCATTCATTCCAATCAAATGATAATTGATAAGTATGTCACATGTCATGTAATTTTGTTTCAGGAACTATTTGATTTTATTGCTGCTGCTTTGGCAAAATTTGTTGCCTCGGAGGGTGAAGACTTTTATCTTCCTGGGGGGAGGCAGAGAGAACTCGGTTTTACGTTCTCTTTCCCAGTAAAGCAATCTTCAATTGCATCAGGCACTCTTATCAAGTGGACAAAGGGTTTTGCAATTGATGAAACGGTTAGCTGTTAGCTCTTGTACTATGTTATTAACAAGTACGCATCACACACAAGAACATACTCTTACAATGGGTAGACAAGACTTGCTTTTTTCCATTGAAAAATTTAAGTTCTTTACTCAAAACCACACCCTTCTCACTCATGGTTGTTAGAAATTCCTCTGGACCATAATAGTTATTGCATGTTGCTGTAAAGTCAGGTCGGTGAGGATGTGGTGGCTGAATTAGGCAGGGCTCTAGAACGTCAAGGGATCGATATGAAAGTCACAGCATTGGTTAGTTTCTTTTGATTTGAAAGTTATAAGATAACTTTTGTTAAATATCCTATGTCAAGAATATGTTTCTGTAACCTGAAGTGCATATATCCCTTCTTATGTAGGTAAATGATACTATAGAGGCATTGGCTGGTGGGAGATACGATGATAATGATGTCGTTGCTGCTGTTATACTGGGTACAGGTACTAATGCGGCATATGTGGATCGTGCTAATGCAATTCCTCAATGGCATGGCCTCCTACCCAAGTCAGGATATATGGTAAGTGCTTTTTTACATCTACCTAGCTCACAAGGCCAAAGCTATACAATTGTGAAATCAGTATATAATACCTGTGGGTTGTGGCCATGTCAAGAAAACATGAAAGTGAAAAACTGGAAAAAGGAGCAACACATCGTCTGTACGGTTATCATGAGGTCATTGATATGGTTGTCATGGTTTTTATCAGTTTTTATGCTTTGTCACAGGTAATAAATATGGAATGGGGGAACTTCAGGTCATCCCATCTTCCTTTGACTGAATTTGGTCCAGCATTAGATGCTGAAAGTCTGAACCCTGGTGAACAGGTATCATACAAAAGAGGTATATCTTCTTTCATTGCCAATTACAACTAATCATAGCATTCTTCCTTTGTAGATTAATGAAAAACTGATCTCTGGTTTGTATTTGGGGGAAATTGTTTGGCGGGTTAATGTAGGGGAGGCCCGATCTTCTCAGTGAGGATCCGTGGATAGTTGATCTGAAATAGACGATGTCGCTAGCTGAACACTCGCACCAATGCACGAACCCTCAACTCTTCGGAATACACCAAAAACACACCGGATAGTTGATCGTCGCGAGACCACAAATGACGATCGTCAAGTCTCCAATTCCCAATGGAACGACGGCTTGACCTTGAATTACAGAACGAATCAATAGTTCGCCTAGTCCTCTCTCAAAGTCCTCGCATGAACTCTGGTTTAGAAAGAACAAGCAATAGCTATGTCTCAATTCTTATGCATAAACTGGTCTGAGATTACAATGGGGGTCAACCCTCAATATATAGGCTCAGCCTAGGGACTATTTGGTACTGTTCATGGTGGTACTATTCATGTCACGCATTGTTCATGACTTGGACTCAAACTCACACAAAAGAAAAACCTAAATTTAAACTGTTACAATGTTTCGGCCATAGCGGCGCGACCGTAGGTCTTCTAAAGCTAGGGCTAGATCCCTTGTGTGGGCCTTGGAATAAGCTTTCCAACAAGCACAAGAACACCCCAATCGGACACTGTATGTAAAAGTTATGACCTTTTTATTACGGGCCTGTTGGGCTGTCCCGAACTGAATCCGAATTGAATTATGAGTTGATTCTGAGTTTGATTTCGGTTGGACTTGTGGGAGGTCCGAATTAGGCTTAGAAATATTATCCAAAACTTGGGCTTCGAATTCTTGAGCTTCTCCCCTTTTCCTCTCAGGGCCGCATGGGCTTCTTCTCCTTTGTTGGCCGTATGTGTTGCCCTCCTTCATGACTTTGTCCCTGGCCTCCCTCTCCTCCCTCTCGGTTCCAAAGTCTTTAAGTTTGTCAACACTGAGAATGCACCAAGTATTGCTTTTAGGTAGTAACCAATTCTCATTGACAACACAAGGAAATTCATTAAGAAACAAATACACCTGTTATTTGAGAAGTTTAGCTCTTGCTTTATGAACAGCCTCAATATTAGTAGGGATATCCTCATCACGAGTCCTGTTAAAGATGAATCAAGAAGCTTCTGTTTTTGGTGATGTTGTACCACCAAAACTCAAGATTCCGTTTATTCTTAG

>PeHXK3a

TTCTATTTCTCCCCCTCCCTTCTCGCCCTTTCCCTTCTCCGCCTTTCCCCTTCGCTCCCAGCCCCACCCCACTACTAGTCTCCGCCGCTCTGTTCCATCCCATTCGCCATTCCGTCGAGCACCCTGTGCGCGCCGCCGGCCGCCGCGTCCTGGGCGCGCGGGGTGAGGGGTGGATTTGTGGTTTGAGAGAGAGAAGAAGGCGCCTGGGATGGCGTGATCGGCGCGCGCCGGAGGGGCGGGTATGGGGAGGGTGGGGTTCGGGGTGGCGGTGGGGTGCGCGGCGGTCACGTGCGCGATCGCCGCGGCGCTGGTGGCGCGCCGGGCGTCGGCGCGGGCGCGGTGGCGGCGGGCGGTCGCGCTGCTGCGCGAGTTCGAGGAGGGCTGCGCCACGCCGCCCGCGCGCCTGCGGCAGGTCGTCGACGCCATGGTCGTCGAGATGCACGCCGGCCTCGCGTCCGACGGCGGGAGCAAGCTCAAGATGCTGCTCACCTTTGTCGACGCGCTCCCCAACGGGTACGGCCCTGCGGTTCCTTACTACTCCCTTTGCCCCCCTGGTTCGTGGCCTCGCGATTCCTTGTTGATTTGTAGAGCTTCTTCCTTTCGGTTCCTCTTTTTTTTGTGTTTCTTAATTGGAACAAAAAACAGCTTTATTATTTTTTTTGCGTTGTTTAATTGTGACGTTGGTAGGTGGATCATACCCTTGCATCTCGGTTGCCATGTTCATCTGGTGTGCGTATGCAAATGTGGCTTCTTTATTGGGTTCTTGCTGCCTTGCCTTCGGAATGATGTCTAGCGATGCGCATTTCACGCTTTTCTTTTGGGTGTTCGTGTGGAATGCTACGAGCACAGATTTGCCTTGTGGTTTTTAAGCTCTCTCTAGCTGAGAGTTTCTTACTTGACGCTTTCTTTTGATCGGTTTGTTTTGCATCGGCGCACAACGACGCGCATTCTCTTGCTACTCCCCCCGCCCCAACGCGGCGTGCATTGGGCTTATCATTCTTTGGACTAGTCGTCTGAATGCTACTAAGTTGCATCGTTTCACATGTTTTGTTTATCCCTTTTGTTGTTCTTGCTTCGTGCTGGGGGTCGCTGCTTTTCTGATGTGTTCATCCAACAGTAAACAAATTTGGGCCGTTACTTTTCTGATATGTTCATAAAGTCGCTATCTTTATTCGATTCCGTATAATGGCGCGCACATCTCGCTGATTTTCTTTCTGAAATGTTCTCAAATACTACGTCATTAGTTCATAATGTTGTCTTTCTTGGAACGGCAAAGAACGACACCCATTTCTCGCTTCGTCCCCGCGAAAGATTACAAGCTCTAGCCTTGTTTAATCGCCATTCTTCCAAGGATTTGTATAGTTCTGATGTCTGATGCCGCCTGCAGTGCTATACCTCTGTGCCGGTGCTGCCAGATACCAAGAATTTGTCATGTGACTCTCCGCCTGAGGCAGGCACTGTTGGTTTGACGCTTGCATTCATGGCGGGGTTGTTGTCCGGACCGCCATAAAGCGAGTAGAGTGTAGCATTAGCATCTTGGTACATGAATCTGCCTACGCTTTTATGTGGCCGGCATTGGGTTCACAGGCCAGGTTGTATTCCACAATGGCAATGTGGCCTGGGACGTTCTCTGTCAGATGGGAATCATTGCTTGGGGGGTCGCACTTCTTAGTCGGAAGAATATCTGGTGTTGATTGTGTGTACTATGCAGAAAATTTTGTGTGTTTTTGTGGACGAGGATCCTCAGCTATGAAGTTAAAACGGTGGAGGATTGTGCTGTTCTGAATTTATCTGATTTTAAATCCAACAATTGTGATGAAGACCAAACATAGACTGACTTGCATGGTTCATTGAGTTGCACATTCAATTTTTTTTTTTTTTTGCTGAATTGCGGCCATCGGTTTCTTGTATGGTTGCACTTGCAACAGCTTGGCGTGCTGTTAGAACCTAGAACTGCCTGCCAGACGGATGCACATTGGTTTTGGTTGGCCTTTTGTATTTTTGTTTCTGTCACATCCCACATGTTTGGATTCTAGTTCTTCGATGTTTCTTGGTCATTGCCAGTATGGCTTCTTAGTAGGTATTCGTTCACTTTGTTCCGATTTAGTAACGTATTGTAGATGGAAAGGATTCAAACTATATTTCTTCTGGTAGCATAGCTTTGTGCAAGATGTAGTGTGCTATGAAATGTGAAGCAATATCTTAATTAATCTTGAGCAAAACCATATTTGCAAACAAATATGAAACCCCCCTTAGTCAGGGCACCGATCTAAAGCATGAAAGCAGCGCTACATATTGTTTGCTTTCTTTTACAATTCTTTACCGATCATGCATCAATTTGTTGTCCTGTTTAAGCCTGTCCAATTCCATAATTTTGATCAGATTTACGCTTGTGCCATGTTGGAGTTCTATTACTAGTATTTTCGTTAATATTTTGCATTTTGGGTTAAAATTGGACCCCTGAAAAATACCACATCTTTGTATTTTGGGGAGGTGTGCATTCTCATAGCAGCCTAGGAACACAATTGTGCTGTCTTCCCAAGACATTTATAATTATTTTAAAACCAAATGCAGCTATAAGCTATGTCCTCTTAGACCATCTCCAACGTTATGTTTTAGCTGGTTCAAACGAGGAGAGAAAAGATATTGAATCGATTCAATACATTGGAGGGGTCGATTCAAACAAAAAAAAAATGCATGTAACCGGACTCTACTTAGGAGTCGATTCAAAGCACTAAACAACTCATTTGAGCATTAAACAATTACATAGGAGAGAGTGCGGTTGAGAGATGATATTATTATTTTGTGGTTTTTTAATGTAAAGTGGGTTCCAGTTATTAGTAGCAATCGGTTCAATACGTTGGAGAAAAACAAGCTCAGTTCCTAGAATGCTTACGTGGCATGATTTTGAATCGATATCGGTTCAATACACTAAAGATGCTCTTATGTTTGTTTTTTTAAGTACAACAATCAAAGCTTTTTTTAGGGGAAGTACAACAATTAAAGCTTATCACTTCAAATACACCTTTTCTTTACTGGCATTTTGTCGTATGATGTTTGATGCCATGGTTCATTAATTTTACACATACCATGATCAATGGAGAAGTTATTGACAATGCCAATGCATTTTGTCCCTTTTTTTTGCATTGTGTTTATGGAACAATCATACTAAATTGGCTCTGGTCATGCACTTCAACAGAAATGAAGAAGGTATTTATTATGCCATTGATCTTGGAGGAACAAACTTGAGAGTCTTGAGAGTAGAAGTTGGTGCGGGGTCTGTAATCGTCAATCGGAAGGTTGAACATCAACCCATCCCTGAGGAATTGACCAATGGTACAACTGAGGTACTTGTTTAATCATATTATCTCCATTTTGTGACATATGTTTTGAGTACATGTCGCTTAGTTACATGCCAATTACAGGATTTATTCAACTTTGTTGCGTTGGCACAAAAGAATTTTGTTGAAAGAGAAGATGGAAATGATGAAAAAAAGGCACTTGGTTTTACATTTTCTTTCCCTGTTAGACAAAATTCAGTGTCTTCAGGGTCATTAATTAGGTGGACTAAAAGATTTTCAATTGAAGACACGGTAAGCCAATATTATTTTCTTGCACAGTTATACCTTCCGCGAATCTGATTTCCATGTTATTCAATTAGTAAATATAACTGAAAAAGTTGTAATATTGGTAAATATCGTAAATTTTCAAAATTGCTAACACTGTCTTGATGATCCTGAGTAGATTGATAAATTATTTTGATAAAGAGGTAAATTATTACATGTATAGAAAGTGAGCATGATACTTAGACATGTAAACTTGCCGTGTTTTTCTAATATGCAAGATATTTGTTATGGGCGTAAGAATGTGCCTGTAATTAGCCCTTCATTTCTCAACTTTTTAACCCAACTATACTTCTTTTGAAAGGAAGCTCATATTACTTTGTTTTGGTGTAGAAAACTTTTCTGCTTCAATTGACATGCAAATTTTTAAACACTTGGTAATGTGAATGATGGAACCTCAGGTTGGGAAAGATGTTGCTCAGTGCTTAAATGAAGCGCTTGCTAGGTGTGGATTAAATATGCGAGTCACTGCACTGGTAGGTCAAATAATTGCCTTTATCCTTCATTTCATTGAGAACTACATTTGGCTGCTTGAAACACTATTATTTAGTGCTTGTCCACTATTAATGTGGACACCTTACTTTCTGTGTAAATACATTGTCAATAAATAATGGATTCAGAAGATGATGCGTAAAGATTAGGAAGTTATAAGGGATAACTGAGAGATTATCTCCTAGCTGCAGCTTATGTATCCAAAAATATTTAGTCAATAGTTGTGAGATTGCTTGTAAGCCCTGGTCTTTGTGTGTTGTAATATGCCGCAGGAAGAGAGGAGAGATAAAATTAATAAAGTATGGTAAAACGAAGAAAAAGTGGCTAAGTGTTGCTTTTTGCTTATGATTTAGATGGCCATGCACAGCCATTCCTTTTTGGGGTTAGGGTGATGAAACTTAAAGGAAAAGATCCGTTTTACACCCCTGAACTCTTTCGAAAGTTCGGAAATCAACACTGAAGTACAAAACCGGACACCCAACCCCCCTCAACTCGCAAAACCGTGCACTTTACCCTCCTCAACCCAAACTGGGGTGGTTTTGTCTGACGTGGCGCTGACGTGGCACTGGAATTGGCGGTTTTGGGCTGACTTGACGCCATGTTGGCAAGGGATTGCCGATGTGTCGTCTATGTGGATCCTAGTTGGAATCACATGTGGAATTTTTTTCCTTCTTTCTTTTTCTTCTTCCTCCCCGAGCTCCTCCTTCCTCATCCCGAGCATCGACAGCGAGCTCTTCATGGCACTCTCCTTCTCTTCGTGGCTAGTCGGTGAGGAAAGAAGAGAAGGCTGAAGACGACGCCAGGAGCTGCACCATCTCTGCGACGGTTGTCGAGATCCGCGCCTCCTCCTCCACCGTCCAAACAGCCATGCCATCGCCTCCACCGGTCGTGAGCCGTGTCGCCCGCCTCCCTCGATCGCGCGCTGCCTCCGACTGGGATCTATGTCACTGCCTCCTTGTCGCAGGCGGCCCTCGGTCGCGCGCCGCCTCTGACTAGGATCTATGTAGCTGCCTCCCCCGGCCGCGACCGCGAGCCACACCACCTTCTCCATCGGCCGGCAGCTGCATCGTCGCCTCGGTCGGCCGTGAGCTGTCTTCGTGGAGAGCCACGCTGCCACCTCTACTGGTCGCACCGCGCCGCTGCCTCCTTCGGTCTCGTGCCACCTTCAGTAGGGAGCTGTGCCGCTACCTCCTTCAGTCGTGAGCCGAGCCACCTCCTCCACTGGGATCCAGGGGACGGTCGTCGGGAGCTGTGTCATCTTTGCAATGGTCGTCGGGAGCGGCAGTCGGATTTGGGGGAGGCCCCGAGGGGGGGGGGGAGGGACAAGAGAGAGAGAGGAGCACGGTGGCGCCAGATCTCCGAGGTGATTCATAAGACGGAGGGAGAAGATTGTTTTAGTGGATGACATGTGGGCCTTATCAACCTTTTTTTTTCCTGATTCATATGGATGACTAGGAGGTGATTCATATGCCATGTCATACTGAAACATGTAGATGTCACCTTGGTCAAAACCGGGTGGTTTAGAGCAGAAGGGGTAAAGTAAACGGTTTTGAGAGTTCGAGGGTGTCGAGTGTCCGATTTTGTAGTTTATAGTTAATTTCCGAACTTTCGTAAGAGTTTAGGGGTGTAAAATGGACTTTTTTCCAAACACGAAACTTAAAAGGCTTTAGGTTGGTTTTACAGCATGTTTAAATGTGAAACTGAAACATTAAGTTGAATAATCCTTCATGCTAGTGTTCTCTTGGTATCATGTAACTGTGTAATATCCTTTTGCTGAATTCAACATGATTCAAAATATGAAAAATGCCAAGTGTCTTGGCCTGATGCTATGGCTTCCTCCTTTTGTTTTGTACTTTAGGATTGCTGAGTATTGATTTCGTTTACCAAATTATGCAAATTTGCTTTTCATACCAGATATTTCATTCATTTACAGAGCTACCCTAAAAAAAACTCTCCTTAATATATCCTAACAAGCATGTTCTGCTGGGCTACTTGTCTATTTGTTAATATGTATATTAACATCAGCGCCTTTTAAACGATTATCTTTTATGTTATTTTTGTACTTAGGTGAATGATACTGTGGGGACATTAGCTCTAGGGCATTATTATGATGAGGATACAGTGGCTGCTGTGATCATTGGCGCTGGCACCAATGCTTGCTATATTGAACGCACTGATGCAATTATTAAGTGTCAGGGTCTTCTTACGAACTCTGGAGGCATGGTATCTTATTTTCTGCTTTTTCTGTAGCTGGTTTGATTTATCTGATTAGATGTTCCTGTGGAAAAAAGGAACTATGATGCTCCTGTCCTACCATAAAACTTCCATTCATCAGGTAGTAAACATGGAATGGGGGAATTTCTGGTCATCACATTTGCCAAGAACTCCTTATGACATCTCTTTGGACGATGAGACACAAAATCGCAATGATCAGGTAGTAAGCTGTGTAAAAATTTCTTATGCATCTTTGTATGTTGGTTAATAGGCTGAAAATTATTTGTCACCATTGTCAATATACAGGGTTTTGAGAAAATGATCTCAGGGATGTATCTTGGGGAAATTGCAAGACTGGTTCTCCATAGAATGGCTCAAGAATCAGATGTTTTTGGTGATGCCGCTGATGGTCTATCAACTCCTTTCATTTTGAGGTATATTCATCCTCGTGATGCTTGTTTCTTCCAAATTATTATTATTTTTTTGTGGAAAAGAGAATAGAATTCATGGTAGTTTATTAAGTATCATAGGAATGACTGGTTTCTAGTTAGCACTTCGGACCTGATTCTTTGTTCTTGAATTTGCTTGATTCTTTTGGATATTTCTTGTAAGATTTCTGCAGCTCCTATGTTATGTACTGGTGCTTATCTGCTTAACCGAGTCCTCGGAAACCATGCAGCTCAGCTGCATATTTTACTGCAATATTTCGAAATCTTGGATCTCCTGGCATTTCCAACCCCATCCTTCTGAGGGAAATGCCGATTGTCTTGATCTACACGAGTTCCTAATCAAAGCTTACCCGCCAAAAACTAGTAATAATTGTAATATGTGTTTTCTGATTCCCTTTTGCAATCCAGCACACCATTTCTGGCTGCAATTCGCGAGGACGATTCACCAGATCTGAGCGAAGTCAGGATGATACTGCGAGAACATCTGAAGGTTTGCTTTCTCAGACCACCTTTATACTTGCAGAGTTTCTCCTACAACAATCCTTCCAAGGCATGCATTTGATTGTTTCCATTCCAGATCCCCGACGCCCCTCTGAAAACTCGAAGGCTTGTCGTGAAAGTTTGCGACATCGTCACCCGCAGAGCTGCCCGTCTAGCCGCAGCTGGCATAGCGGGGATACTGAAAAAGCTCGGGCGGGACGGGAGCGGCGCGGCCTCGAGCGGGAGAACGAGAGGGCAGCCGAAGAGGACGGTGGTGGCGATCGAGGGCGGGCTGTACCAGGGGTACCCAGTGTTCAGGGAGTACCTGGACGAAGCCCTGGTGGAGATCCTGGGGGAGGAGGTGGCGCGGAACGTGACGCTGAGGGTGACGGAGGATGGGTCGGGGATCGGAGCTGCTCTCATCGCCGCCGTACATTCGTCGAATAGACAGCAACAAGGAGGTTCCATATAG

>PeHXK3b

CGGCGGCCACGTGCGCGATCGCCGCGACGCTGGTGGCGCGCCGGGCGTCGGCGCGGGCGCGGTGGCGGCGGGCGGTCGCGCTGCTGCGCGAGTTCGAGGAGGGCTGCTCCACGCCGCCTGCGCGCCTGCGGCAGGTCGTCGACGCCATGGTCGTTGAGATGCACGCCGGCCTCGCGTCCGACGGCGGGAGCAAGCTCAAGATGCTGCTCACCTTCGTCGACGCGCTCCCCAACGGGTACGGCCCTGCGGTTCCTTACTGCTGCCTTTGCATCACTGGTTCGTGGCCTCGCGATTCCTTGATGATTTGTAGCGCTTCTTTCTTTCGGTTCCTCTTTTCTGTGATTCTTAATTAGAACAAAAAATAGATTTTTTTTTCTGTTCTTTAATTGACATGTTGGTAGTTGGATCATACCCTTGCATCTCGCCATCTTGCTTGGTTGCCATGTTCATCTGGTGTGCATATGCAAATGTGGTTTCTTTATTGGATTCCTGCTTCCTTGCCTTCCGAATGATGCCCAGTGATGCGCATTTCACGCTTTTCTTTTGGGTGTCCGTGTGAAATGCTACGAGCACATATTTGCCTGGTAGTTTTTAAGCGCTCTACTTGAGAGAGTTTCTTACTTGACGCCTTCTTTTGATCGGTTTGTTTTACACCGGCGCACAACGACGCGCATTTTCTTGCTACTAAGTTGGATCGTTTCACATGTTTTGTTTCTCCCTTTTGTGGTTCTTGCTTCGTGCTGTTGTCTGGCCCTATTGTGGAACGATAAACAAATTTGGTTCGCTGCTTTTCTGATGTGTTCATCCAACAGTAAACAAATTTGGGCCGTTACTTTTTCTGATATGTTCGTAAAGTCGCTATCTTTATTCGATTCCGTATAATGCCGCGCACATCTCGCCGATTTTCTTTCTGAAATTTTCGCAAATATACGTCATTAGTTCATAATGTTGCCATCTTTCTTGGAAAGGCAAAAGAACGACGCCCATTTATTGCTTCGTCTCCGCGAAAGATTACAAGCTCTAGCCTTTTTTAATCGCCATTCTTCCAAGGATTTGTATAGTTCTGATGTCTGATGCCGCCTGCCGTGCTATACCTTTGTGCGGGTGCTGCTCCGGTGCAAAGACAATTAATCTGCCAGATAACAAGAATTTGTCATGTGGCTCTTATGCCGCCTGAGGCAGGCACTGTTGGTTTGACGCTTGCATTCATGGCGGGGTAGTTGTCCGGACCGCCACAACGGGGGTAGAGTGTGGCATCTTGGTACATGAATCTGCCTACACTTCTATGTGGCCGGCATTGGGTTCACAGGCCAGGTTGTATTCCACAATGCAATGTGCCCTGGGACGTTCTCTGTTAGATGGGAATCATTGCTTGGGAGGTCGCACTTCTTAGTTGGAAGAATATCTGGTGTAGATTGGGTCTTGTAATGCAGAAAATTTTGTGTGTTTTAGTGGACAAGGATCCTCAGCTATAAAGTTAATATTGTGGAGGATTGTGCTGTTCTGAGTTCATCTGATTTTAAATCGAACAATTGTGATGAAGGCCAACCGGAAACTGAGTTGCATGGTTCATCGAGTTGCACATCCAATTTGAACTTTTTGCTGAATTGCGACCATCGTTTCTTGGATGGTTGCACTTGCAACTGCTTGGCATGCTGTTAGAACCTAGAACTGCATGACAGACGGATGCACATTGGTTTTGGTTGGCCTTTTGTATTCTTGTTTCTGTCACATGTTTGGATTTTAGTTCTTGGATGTTTCTTGGTCATTGCCTGTGTGACTTCTTAGTAGGTATTGATTCACTTTATTTCCTTTTAGTAATGTATTGTAGATGGAAAGGATTCAAAGTATATTTCTTCTGTGTAGCATAGCTTTGTGCAAGATGTAGTGTGCTATGAAGTGCGGAGTATATCTTAATTAATCTTGATCAAAACCATATTTGCAAACATATATAAAACCCCCCTTTGTCTGGGCAACAATCTAAAACATGAAAGCAACGCTACATATTGTTTACTTTCTTTTGCGATTCCTTACTGATCATGCATCAATTTCTTGTCCTGTTTAAGTCTGTCCAAGTCCATAATTTTGACCAGATTTCAAGTACACGACAATGTTTATTGCCTTATTCCATGCTGGAGTTCTATTAAAAGTATTTTTATCAATATTTTGCATTTTGGTTTGTAGTGGTTAGCGAGAAAAACAGAAATGACACTCGTATTTGCTGTAATTGCACATTTGCCCGCACCACATTGGACCCCTGTACCACTTCTTTTTATTAGTTCTTGCCTTAATTACGGTGTATATCACCCCTGGAAACAGAACAATTGCGCTACAATAATCGATTTGCATATCCGGCCTGCAAAATTGAGGAGCCATCTGTGGTGTCCTGGCTGCTGGTGTACAGGATAGTGCATAGACAATGAGTCAGGGATAGCGACAAAGTGTGGGCAGGCGTGTTGACAGCTAGCACAGACGGCAACAGAGATGCGTGCACGGAGGTGGTTTTCAGTTGCCATGAAGGTGGCTCTGCATGGCCAAGCCTGGTGGTAATAACGGACACGATGAGGGTTTGGTGAGGACTAGTGCACATGAAGATGGTGAGGCACAGCTGGGTACATTGGCGGCTCTCGCTCCCCTCTGTGTCTTGTCATTGCATGCATCAGCAGCTACTGCTCTCTTGCGTGACTTGTGTAACTATTGTTTGGCATCATCTCTATTTTAAAATTGGAGTTTGATTTTGCTGGGCCTTGGAGCAAATCTGTTACAACTTTTAGGTCGTCCAAATGTCTAATACAGGCCACCATAGCCCAAAAACTTATTGAAAACAAAAGAGTATTTTCTTGACGATTCTATTTGGGGAGGTGTGTACATTCTTGTAGGAGTTAAAGAGCATAATTGTGCTGTTTTCCCAAGGCATTCATAATTCTTTAAAACCAAATGCGTACAACAATTGAAGATTATCACTTCAAATATACCTTTTCGTTACTGGCATTCTGTTGTATGATGCCATGGTTCATTAATTTTACATGTACCATGCTCAATGGAGAAATTATTGACAACACCAACGGTTTTTTTGTCCCCCTTTTTTTGGCATTTTGTCTATGGAACAACCATGCTAAATTGGCTCTGGTCATGCACTTCATCAGGAACGAAGAAGGTATATATTATGCCATTGATCTTGGAGGAACAAGCATTAGAGTCTTGAGAGTAGAAGTTGGTGCGGGGTCGGTAATCATCAATCGGAAGGTTGAACATCAACCCATACCTGAGGAATTGACCAAGGGTACAACTGAGGTACTTGTTTCATCATATTATTTCCATTTTGTAATATATGCTTTGAGTACATGTCTCTGAGTTCCATGTCAATTGCAGAGTTTATTCAACTTTGTTGCCTTGGCACTGAAGAATTTTGTTGAAAAAGAAGATGGAAAAGATGAAAAAAGGGCACTTGGTTTTACATTTTCTTTCCCTGTTAGACAAAATTCAGTGTCTTCAGGGTCATTAATTAGGTGGACTAAAGGATTTTCAATTGAAGACACAGTAAGCCAAAATTATTTCTTGCACAGTTATACCTTTCACAAATCTGATTTCCATGTTATTCAATTAGTATGTAGAACTTATAATGTTGTAATATTGGGAAATATCTCAACTTTTCTTATGTCATAAGGTGAACCCAAAATTTGCTAACATTGTCTTGATGATCCTGAGTAGATAAACTATTTTGATAGAGGTATATTCTTACATGGATAGAAAATGGGCATGCTACTTAGATTTGCAAACTTGCCATGTCTTTCTAGTATGCAACATATTTGTTATGGGCATAAGAACATGCCTGTGATTAGCCCATCATTTGTCAGCTTTCTAACTCAATTATATTTCTTTTCAAAGGAAGCTCATATTACTTTGTTTTGGACTTTTGGTGTATGATTGCCTTTCTGCTTCTGTTTGACATGCTAATTCTTAAACACTTGCTCATGTGAACCATGTAACCTCAGGTCGGGAAAGATGTTGCTCAGTGCTTAAATGAAGCACTTGCTAGGTGTGGATTAAATGTGCGAGTCACTGCACTGGTAGGTCTTTCCCAACTTTTTGTGGCTTAACACCCAAAATAGTTACTTTTATCCTTTCATTCCATTGAGAACCACATTTAGCTGACTTGAAACACTATTATCTAGTGCTTGTCCGCTACTAATGTGGACACCTTTTCTTTCTGTGTAAATACATTGTCAACAAATAATGGATTCAGAAGATGATGCATAACGTTTAGCAAATAACAAGGGAGAACTGAAGGAGGATCTCCTAGCTGTAGCAGCTTATGTATCCAAAATATTTAGTTCATAGTTGTGAGCTTGCTTGTAAGCCCTGGTCTTTGTGGGCTGTAATATGACGCAGGAAGAGAGGAGAGATAATTAATAAAGTTGGGTAAAACTCGAAGTGTTGCTTTTTTCCTTATGGTTTAGATGGCCATGCATGTGTCATTTCTTTTTGGGGGTAGGGTGATGAAACTTAAAAGACAAGTAATCAGCTTGTAAGATGCTTGGTTTTACAGCGTGTTATGTTTAAATGTGGAACACTAAGTTGAATAATCCTTCATGCTGCTGTCACCACCAATGGTAGTTAATGTATGTATGGTCATAATAGCTTTCTTAAATGATGAAATGTCTTGGCCTGATGCTATGCCGTCCTCCTTTTGTTTTGTACTTTATGATGGCTGAGTATTCATTTGGTTAACCAAACCATGCAATGCAAATTTGCTCTTCATACAAGATATTTCATTCGGTTACAGAATTACCCTAAAAAACTTTTCTTCATATATCCTGAGTCCTGACAAACATGTTCTGTTGTGCTTCTTGTCTATTTATTAGTACATATATTAACACGTATTGACACCAGCGCCTTTTAAATGATTATCTTTCATGTTATTTTTGTACTTAGGTGAATGATACTGTGGGGACATTAGCTCTAGGGCATTATTATGATGAGGATACAGTGGCTGCTGTGATCATAGGAGCTGGCACGAATGCTTGCTATATTGAACGCACTGATGCAATTATTAAGTGTCAGGGTCTTCTTACAAACTCTGGAGGCATGGTATCTTATTTTTCTGCTTTATCTGTAGTGGGCTTGATTTATCTGATTAAATGTTCCTGTGGAGAAAGGAACTATGACGCTAGTGTCTTGCCCTAAAATGTTGCATTCATCAGGTAGTAAACATGGAATGGGGGAATTTCTGGTCATCACATTTACCAAGAACTCCTTATGACATCTCTTTGGATGACAAAACACAAAATCGCAATGATCAGGTACTAAGCTGCGTAAAATTTCAATTTCTTGTGGCATCTTTGTATTGTTGTTAATAGGCTGAACATTATGTGTCACCATTGATACAGGGCTTTGAGAAAATGATCTCAGGGATGTATCTTGGGGAAATTGCGAGACTGGTGCTCCATAGAATGGCTCACGAATCAGATGTTTTTGGTGATGCTGCTGATATTCTATCAACCCCTTTCATTTTGAGGTATCTTCATATTTGAAATGTTTGTTGCTTCTAAATTACTATGTGTTGTGGAGAAGAGAATAGAATTTATGATTCCTGCTAGTTTATTTCTGCATATTATTGGGGTAGCAATGACTGCATAGTACTTCGGAGCTGACTCTTTGTTCTTGAATTAGCTGGATTCTTTTGAATATTTCTTGTAAGATTTCTGCAGCTCCCATGTTATGTACTGGTGCTTGTCTGCTTAACCATGCAGCTCAGCTGACCTGATATTTCAAATTATTGGATCTCTTGGCGTTCCAAACCCATCCTTTTAAGGGAAATTTCAGATGACTGATTGTCTTGATCTACACATGAGTTCCTAACAAATAGCTTAATCACCAAAAACCAGTAATAACTGTAATATGTTTTTTCTGATTCCCATTTGCAACCCAGCACACCACGTCTGGCTGCAATTTGCGAGGACGATTCACCAGATCTAAGCGAAGTCAGAAGGATACTGCAAGAACATCTAAAGGTTAGCTTTTCCCTGGTCTCCTTATACTTGTAGGGTCCCCTTGTACAACAATCTTGTGCTTTTGCTCCGACTCTAGTTCTTCCAAGGCATGCATCTGATTATTTCCATTCCAGATCCCCGACGCCCCTCTGAAAACTCGAAGGCTTGTCGTGAAAGTTTGCGACATCGTCACCCGCAGAGCTGCTCGTCTAGCTGCAGCTGGCATCGTCGGGATACTGAAAAAGCTCGGGCGGGACGGCAGTGGTGCGGCCTCGAGCGGGAGGACGAGAGGGCAGCCGAAGAGGACGGTGGTGGCGATCGAGGGCGGGCTGTACCAGGGGTACCCAGTGTTCAGGGAGTACCTGGACGAAGCCCTGGAGGAGATCCTGGGGGAGGAGGTGGCGCGGAACGTGACGCTGAGGGTGACGGAGGATGGGTCGGGGGTCGGAGCTGCGCTCCTCGCCGCCGTACATTCGTTGAATAGACAGCAACAAGGAGGTTCCATATAG

>PeHXK4

ATCATTTCTTGCCACCTTCCTCCATCCCATCACATTACTTACATCGACCACTCCTAGCAGCTAGCTGCTCCTCCGCCTCGGCATGTCCGCCGCCGTATGCTCGCCGATCCCGGCCGCCACCGTCGCGCAGCACCGGCGGAGGTGCGGCGCCGCTGTCCGGTGCTCCGCGGTGGCCGCGCCCATCCTGAACGACCTGAGGCTGCAGTGCGCGACGCCGCTCCCTGTGCTGCGGTGCGTGGCGGACGCCATGGCTGCCGACATGCGCGCCGGGCTCGCCGCGGACGGCGCCGGCGAGCTCAAGATGATCCCCAGCTACGTCTACTCGCTCCCCACGGGGTAACTAACTTAGCCTCGTTGTTAGTGATACTGTGAAGTGATTAGTAAAGCATATGTATGAGCTTTTCTTTTAGCTTGAATTTATTATATCTCTGTATACAATTGGAATTTCAATTGAAAAATTGGACTAGTGAGAGTTAGACTTTGTTTTCCTGTTAAGATCGTCACTTAAATCAATTCATTTCATAAATCAAAATCTTCTCTCTTGTCGCTGATTATTTTTTGCATCACAATTGTTATGCTGAAGGGATGAAACAGGGCTGTTCTATGCTCTGGACCTTGGAGGCACAAACTTTCGGGTGCTGAGGGTACAATTGGGAGGAAAAGATAAGCGTGTTGTCGACACCGAGTTCGAGCAGGTCTCAATCCCAAAAGAAATCATGCATGGTACAACAGAGGTAATTGGTTTTATCAAAAAAATTATTACCTTAATCTTATTCATTCTCTTTCTACTTCTAGAGAGGAAATGTTGTGCACGCATTGACACGTGGATGCTGTATATAGTTGGGAAATGTGTATCTGACAGAATAGTTATCTCAAACAGGAGTTGTTTGATTTTATCGCGTCTGGCCTGTCGAAATTTGTAGCAAAGGAGAGTGATAAGGTTTGTCTTCCGCAAGGATGGAAGAGGGAGATAGGCTTTACATTCTCCTTTCCGGTGAAGCAGACTTCTATTGATTCTGGCATTTTGATCAAGTGGACAAAAGGTTTTGCTGTCTGTGGGACTGTAAGATTTTTCTTTTTTCCTTATACATTGTTTCTGTACCAGTTTGGACTCTTAGGCATGGACGTTCATATATATATAAACCATTCTCTCCTTGATACGATCCAACGTGGCTTTTTATTTATGCTTTTCACTTCTTCAGTGATTCTCCTTGACTCCTAGTACGTATGAACTGTTGACAATTGTTCCATCACGAGAGATATTTATCCAAACTAATGTGATAAATTCATCTAACGCTTCCTTTATATATCATTGTTGAAACAAAGAATTGTAAGACTCAAATACCTGCTAGAACCTTTTAATACCGCCAGGATTACAGTTTAAGAACCAGCAAATCACGTTTCTTTTTCTGTTAAATGTATGCTAATAACAAGCAATATGTTGCTCTGGACAGGCTGGGAAAGATGTGGTTGCTTGTTTAAATGCTGCAATGGAGAGACGGGGGCTTGACATGCGTGTATCTGCCTTGGTAAGAAAGCCGCAAGCCTCCTTATTCGAAAAGCGTATGATATCTCCAAATACAGATACAGAATTCTGAAAAGGAAACGTTGTGCTCTGACATTTATGGGCTAGAACTTGTTTAAAGAGACATCCTTTCCATAGGAAATTTAATTTGAGTAAAATTCCACGTAACAGGTAAATGATACTGTCGGAACCTTAGCTGGAGCACGTTATTGGGATGATGACGTGATGGTTGCGGTGATTTTGGGTACTGGCACAAATGCATGCTACATTGAGCGAACTGATGCTATCCCAAAGCTGCAAAACGTTATGCCTGGAGCAGGAAACACGGTTTGCTACTTTGTTATAACTGGACACCTTCTTTTATTTACTTCTTATTAAAACAAAAATGCATGATGCAAATCATTTGAATAATGTTTGTAAAAAAACAAGTATTCTGAAGTTGTGTAATTTTCTTGATTGATGTCTCTGTGCTGCTTCTGCAGATTATCAACACCGAGTGGGGAGCTTTCTCAGAAGGTCTTCCATTGACTGAATTTGACAGAGACATGGACGATGAGAGCATCAATCCTGGTGAGCAGGTAAAAACATGTTAATTTGTTATACTTGCATTTGAGTTCGTTTGTTTGTTTTGATGTCATTTTTTGCCTGCAATATCTCCAGATATTCGAGAAGACAATTTCTGGGATGTACCTGGGTGAAATTGTTCGGAGAGTGCTGGTCAAGATGGCTAAAGTATCTGATCTGTTTGGTAATTCTTTCCCTGACAGGCTTGCCATGCCATTTGTTCTAAGGCAAGTTCTTTTATTCCGACTTCCAATTTTACACTGTTACCACATCACATGTCACATGATGTTCTATTCTATTTATAACACGTACAAATCAAATTGTAGGACACCACATCTGTGTGCTATGCAGCAAGACAGCTCCGATGATCTTGGGGAGGTCAAGTTAATCTTGAATGACATCATTTGTGTAAGTTACAAGCTTCTAGTTTTCAGACATAAGGACATATGTATGCTTCATTGGTGCCAATATTTGGCCATGAAATTGGTTGCCCACAAATTAGGAAAGTTTCGGTAATGAAATAAACTTTTCGGTAATGAAATAAACTAAGATGCTAATGGAAACGGAATATTGCACCAAACCAATGCTATCAAGATTTGGCAACTACTGGCTTCAAACCAAATAAACCCAGTTGGTAACCAAATAACCAAAGATATAATTTCCCCTTACACCTCATTATCAGGTGAAGCAATCTTCTATGGAGGCGAGGAGGATCATTGTAGAAGCCTGTGACTGTATTGTAAAGAGAGGCGGCAGGCTGGCTGGGGCTGGCATTGCAGGAATTCTTCAGAAGATGGAGAATGATTCCAAAGGACTGATCTTTGGACAAAGAACGGTGGTTGCGATGGATGGCGGCCTTTATGAGAACTACCCGCAGTACGGGGAGTACATGAAGGAGGCTGTGGTAGAGCTGCTCGGCCCTGAGGACTCAAAGCACATCGTCGTTGAGCACACCAAAGACGGCTCAGGGATTGGTGCGGCACTGTTGGCCGCTGCCAACTCGAAATATGCAGCTCAGTTGTCGACGTGAAAGATGAGAACCTTGAAGTAGGTCTTCTGTTCACTTCAGATTTTGAAGGATGGCGGCTTCATGTATCTGTCCTACTGTGCTTTCTGTTGAGAGAGGAGAAATCAGTTCAGATTTATCAGGTTATGTGTAGAGGTTATACGCGCCTAGCCGCCTAATAGCTAGCTCCCAGGAGGTGTAAAATAGGTGATCAACTTACAAGTATGAAGTTGGTTCAGTTTGTGCTTTTGTATCCAGATTGGAATAAATGGTTCTATGCCACTGGGAATTCATCAGTTTCGG

>PeHXK5a

GGCCGCCCATTTGGCCGCCCTAGATCCAGGCGTGCAAGGGCGGATCCGTAAATGCCGCAAAGCGGAGGCGGAGTTGAGTTTACCGGTTAAAACGCCCGGGTCAGTGGCTCTCGTTTTCCTTCTCCTACGCGGCCGGGGAAGGATTCCACTGGTATTGGCTCGGCTCGGATCCAGAACGCAATTCGTGTGGGGATCGGGCGCTCGGATCGATCGGAGGGGCAGGGGGTGAGAGGGCGCAGACAGTGATGGGGAAGGCGGCGGCGGTGGGGACGGCGGTGGTGGTGTGCGCGGCGGTGGGGGTGGCGGTGGTGCTGGCGCGGCGGCGGCGGCAGCGGAAGGCGGAGCTGGTGGATGCCGCGGAGGCGGATAGGAAGAGGAGGGTGGCGGCGGTGATAGAGGAGGTGGAGAGCAGGTTGGCGACGCCGACGGCGCTGCTGCGGAGCATCTCGGACGCCATGGTGTCCGAGATGGAGCGCGGGCTTTGCGGGGACATCCACGCCACGCTCAAGATGCTCATCACCTACGTCGACAACCTCCCCACCGGGTAAAGTCTTCCCTCCCGATCTGATCTCTGTTTCCTTTCCTTTCATTGGGCTGTGATTTGTGAACTTAGTGATCCGGAAGATGTGCTTCTGGGTGGGAAAGGTCGCGTACGGAGGTGAATTGGTCGCGAAAGTGCTTTGTTGATTGCGTAAGCGTGGGGATTTTTTTTATTAGGCCTTTTAGTGAATTGAGTGGGAATGTGCGAATCATCCTGTGAATTCTTGTTGGAAGCTCGCGCCTTGTGGTGCTTCGAAGCGAAATCGGGGGAGCAATCTCTTATTTGATCTTCGTTTGGTTCTCGATGACTCATGGGCATGGTACCGGTCGTGGTACTTTGAGTAATGGGATTTTGACGAGTTCGTTGTGGTGCTTGGTTTGCAATTCCGGGCGTTGATTTTTCAAGTCTCACGTGGTAGGCCATTAAGTCGGATGGATTAACCAATTTATGGCATGAATATCTCTTGCAGGTTTACTCTCATATATGCTCCATTGCTGTCAATTCTGTAGAAATGTTTATAGGTGTTTTTGGTTTCTTTTAGCAGTTTGGTCAATTATATCCATCACCATCAATTGAACTCATCTCAAGGCATAATTGGTTTAGTACTTAGTTTCTCAAGGACCTCTCTCCGTATCGTATAGTTAAAATGGTCAGACACATTCAGAGAGAAAAATCTTAAATCTAATGATTATTTTGCGGCCAGTCTGTTAAGTGCAAATCACTCATAAGCATGAAGTATAGGTTCTATCAACATTGAGTATTGCCCACTTCAAAGACTCGTGCTTCACTTCATTCTTTAATCATCTGGGTGCTGCTTCTGCAGAGACGAACATGGGTTGTTCTATGCACTGGATCTTGGAGGGACCAACTTCCGTGTTCTGCGAGTCCAACTTGGAGGAAGGGAGAAACGTGTCGTCAAGCAACAGTATGAAGAGGTCTCCATTCCACCACATCTGATGGTTGGGACTTCCATGGTGAGTACTTGCTGTTCGATTTTCATGTTTCTTGGAGTTGTACATTTCATCCACGCTTGCCTTACGTATCTTGATTTTTTGATACAGGAACTATTCGATTTCATTGCTGCTGCATTGGCAAAATTTGTGGATACTGAAGGTGACGATTTCCACCTCCCAGAGGGGAGACAGAGAGAGCTGGGCTTCACTTTTTCCTTCCCAGTGAACCAAACATCAATATCTTCAGGAACACTCATCAAGTGGACAAAGGGCTTTTCCATCAACGGCACGGTAAAAGTCAAAACACTTTGTTCATGCCCCACCCACTGAACACCATCTTAGTACTTTTGTAATTATGGCTTGAACCGGAATTTGTTATAAACACATGAATTCTCCCAGCAAGTTTCCCTGAGAAAGCTTTAGTTAATATGGCAATAGTGCCTCTAGCATTTGTTCCTTTTTCTCAAAGGACTAGCTTTCAGTGAAAAAATTTCTACATTACTCTTTTAGAATGTTAGAGCAGAGCTTTTAGCGTTGAAGTTTAATAGGGGCGTTGCTGTTAAGTATGATTTCATGTTCATTCACTGATTGCTGTATTCACCTATAAACAATGTAATACTTCATTATTTTAATTTTCAATTGTTACAATATGATCAAACTGATTCATTTATCTCAATGAAATATTGAGCTTGGTTAGGAAGCATATTCTTGAGGTGCAGAAAGAATATTATGTTTCTCCGGTTGATGGTATATGTGTCTATTGTCAAGCATTCTATAATTGTCAGAAACTCAATTACTTGATTTGGATATTTATTTGATTGCTCCTGAATCAGGTTGGCGAGGATGTTGTCTCTGAGTTAAGCAACGCCATGGAGAGGCAGGGGCTGGATATGAAAGTTACAGCATTGGTAAGTTAATATCTGTCAATTTATTCAATTTGTACACCATCTATGCTCAGTTTTCGTATCTATAGCATTGCTAGTTTCTTTGTATACTGATGCCGTTTTTGGGTTTTTGATCCTTTTATCTGTTGATTGGATCCAAACATGGTTAATATCTTGCAAATTTGTTTCTTTCCGAGACATTCTCTGCTAGACTGAGTATTGATGTCTTGTAGGTTAATGACACAGTCGGCACATTGGCTGGTGGGAGATATATGGACAATGATGTAGTTGCTGCCGTAATATTGGGCACTGGTACAAATGCAGCATATGTTGAGTATGCTAATGCTATTCCTAAATGGAATGGTCTACTGCCTAGATCCGGAAATATGGTTAGTGTTGGACTTCCTTATGCTTGGAATTATAAAGTTTGTTGAACCATTGTATGGTCTTACACATGCACTGTACCATCACCGTTGTCTGGTATTCACCCCACTTGAAAAATTATATAACCATGGAATTATTTGGTATTCTCTAGGTAATCAACACGGAATGGGGGAGCTTTAAATCAGACAAGCTTCCTCTTTCAGAATTCGATAAAGCCATGGATTTTGAAAGTTTGAATCCTGGAGAGCAGGTATTGTCTCCCTGGTCTTTCCCTTCCCTTTTTGTTTTTTAGGTGAACTGCATTAATATATGTTAAAAACTTTTGTATGCCAGATATATGAAAAGATGATTTCTGGAATGTATCTTGGAGAGATTGTGCGAAGAATCTTACTGAAACTGGCTCACGATGCTTCTTTGTTTGGGGATGTTGTTCCTACTAAGCTGGAGCAGCCATTTGTACTGAGGTATACTTTCTTGATCCATTGGTTGTCAAGCTTTTCATGGCTGCTTACTGACATGGCAAAAGCTGCAATACTTTTCCGTTAGCTAACAAGATGATAAAGATTTTTTTTCACAATTTCTACGTAATCATACTTGGATAAGAATGTGCATGACATAAAGAGTTTGGCTACTTTTCCACGATGTATTTAAGTTGGTGACAATAATAGTCGTACCTGAATAAATTGCAGACACAGAAGTTGCGTGTCTTCTGAAGTTATCATATTACATCAGTTGACTAATTTGTGCAACTCTGTGCTACTCGATGCTTGGTCTATTTTCTTCTCTATGTATTAGCATAACTCCCTGGATGGCGTGTCAATTGTGTGGCTAGATAAATTTTGTTTCATGCACAACTTAATATTATAACCGAGTACTAAAGGTAAACTTAAGTTTTTTTAGTTGAATGGCTCATAAAATTTGAGCAGTGTCCTAATGAGAATAATGTTCCTTTACCTCTCCTTTAGTGGGAATTAGTCGGTTACTTATTTTTGGCCGGTAAGTAATAAATAGGTTATTAATAGATGGATGCATATGGATGCATTGGGTGGTATAGCATACTTGAGACCTGATTAACCCATGCCATTAGTAACAACAAATCTGTGAGGTGTACTTGTTGACAAATGGTTTCTCACACTAGCTCACTTGCAAGAAAAGGATTTTTTTTGTAGAATCTCTAGTACTTTTTTTATTCTTATTCTGCGTAAATCCAGCTTCTGAATTTTATGTAGCTACATAAGCATTTTTATGAATATCTTGATTGTGGCATCACAGGACGCCAGATATGTCAGCCATGCATCATGACTCGTCACATGACCTTAAAATTCTGGGAGCTAAGCTAAAGGATGTCGTGGGGGTATGACTTGTTTGTGCCAAAGTTAATACCACAATTGTTCAATGACCATTGATTGCTTATGTAATATTTGATCGTATTTCCAGGTCCCAAATACTTCCCTGGAAGTAAGATACATTACTCGTCACATCTGCGACATTGTTGCAGAGCGTGGTGCACGCTTGGCTGCTGCTGGTATATATGGCATCTTGAAGAAGCTAGGCCGGGACAAAGTGCCAAGTGATGGCAGTAAAATGCCGAGGACTGTCATTGCCTTGGATGGTGGGCTCTATGAACATTACAAGAAGTTCGGCAGTTGCTTAGAAGCAACTCTCACAGACCTCCTCGGTGAGGATGCCTCGTCTTCGGTAGTTGCCAAGCTGGCCAACGATGGCTCTGGCATTGGAGCTGCTCTCCTTGCAGCCTCGCACTCTCAGTATGCCGAGGTCGACTAG

>PeHXK5b

AGTCAACCCCCCAGATCCACTTACCACTCGCTTCGATTCTTTTTATCCCACCAGAAACGAATCACACCCTGACACCCGTGCGATTCCAAGGGCGGATACGTAAATGCCGCAAAGCGGAGGGGGAGTTGAGTTTACCGGTTAAAACGCCCGAGTCAGTGGCTTTGGTCTTCCTTTCTCCTACGCGGCCGGGGAAGGATTCTACGGGTATAGCCTCGGCTCGGATCCAGAACGCGATTCGTGTGGGGATCGGGCGCTCGGATCGATCGGAGGGTCAGGGGTGAGAGTGTGAGAGGGCACGTGCAGTGATGGGGAAGGCGGCGGCGGTGGGGACGGCGGTGGTGGTGTGCGCGGCTGTGGGGGTGGGGGTGGTGCTGGCGCGGCGGCGGTGGCGGAGGTTGGCGGAGCTGGTGGAGGCCGCGGAGGCGGATAGGAAGAGGAGGGTGGCAGCGGTGATAGAGGAGGTGGAGCAGAGGCTGGCGACGCCGACGGCGCTGCTGCGGAGCATCTCGGACGCCATGGTGTCCGAGATGGAGCGCGGGCTGCGCGGTGACATCCACGCCACGCTCAAGATGCTCATCACCTACGTCGACAACCTCCCCACCGGGTAAAGTCTTCCGTCCCGATCTGATCTCTGTTTCCTTTCCTCCCCTTTCATTGGCTTGTGATCTATGAACTTTGTGATCCAAAAGATGCGGGCTTCTGGGTACGAAAGATCGCGTATGGAGGTGAATTGGTCGCAAAAGTTCTTTCTTGATTGCGTAATCGTGGGATTTTTTCTCCTTGTTCTGATTAGGAATGACTGGCCTTTTGGTGAATTGATTGGAAATGTGCGAATCTTCTTGTGAACTTTTGTGGAAAGATCGCGCCTTGTGGTGATTCGAGGCGAAAACGGGAATTATCGTCTCTAATTTTATCTTCGTTTGGTTCTCGATGACTCATGGGCAAGGATGGGATCTTTTTATCTATTGTGAATTGTGGTTGTTAGATGAGCTTCCGATGCGTGTGATCTCTAGCAGATCGCACGGCAATTCCAACTTTGGTGTTCTAGGATTTCTGGATAAATGACCATCGTACCGGTCGCGGTGTTTTGAGTAATGTGGGATTTTGAGGAGTTGGTTGTGGTTGCGATTCTGCGCGTTGATTTTTCAAGTCTTACGTGGTAGGCCATTAAGTCCGATGGATTAACCAACCAATTTATGGCATACATATTTCTTGCAGGTTTTCACCTATATATGCTCCATTATTGTCAATTCTGTAGAACCTAGAAGTGCAGCTAGCGCATTTGCCTGGCTAGATTTGTTTCGGAGTTTGTATAAATGCTTGAGAATTATTTAGCTATCTTGTAGGCTATTTAGACTTTTAAGATATCCTAAACATTAGAAACAACAATAGAAGTTTGAAGGAAATTACAATAATTGAAGTATGTGCTTTGATTGAAGAAAATTATTAAACATAATAAGTTGTTCGGATTGTTAGAAACTAACCACTTTTCATCGTGCATAAATCATGTAGTTATATATCCCTGTTCTTACTCTATTAGTTATTTTGTTTCAAATCGTATGCATGCACATGTACCTCTATTTAATCTCAAGCAACACATTGATTATGCGTGCGACCAATTATCTTATTAGTTTACATGATATAATGTATGAGAGTACCTTGCTCACGCATGCACTGATGCACCATGAGCGACCTACCTTGAACTTATCTTCCCTCACACGACGGCTGCCCTACTAAACCGTATGACTTCTATCTATTCTCGCCGTCACGTTTTTTTTTTTGCTTCAATCTCCAATAGGAAGCCCAAATCTTGATCGATCCCTCTCAACTGCTACCCCTTCAAATGCACCACATAATTATCTTTTACTTCTTCCCAAATATTGCTTAATGCTAACCATTGCGACATCGACGCGCATAAACATGCAACCGTCTCGATCCCAGCTGGGAACACGTGTCACGCTCTCTCCGGTGACCCCGAGCATATTCCTTACGACGCCATCTCTACTCCAAGCTTTTTGCCCCTTTGCCTCTGTACACCTGCGCTTCTATTCGTACGGATGCATGTATGTACATACGCATGTATGATGTTATACATGCTTGATGTCCTCTTCTCTCGTGTAAGTCGCTCCTCTTCTTCGACATTGGCCAACTTAACGTTGGAGCTTCCTCACTAACCGTGGCGACCTCCCGTCTTCACTAATATCAATATGTCTGTTTTAATCGTTGTCTAATAATATGGAGAATATTTTTGCCATCGCTGGCGACAACGTGCCCTCACCAGTGATGACACGCTCCTAGCTACCCGATGGCCTCATATCATCCATAGTTTGTCTTCAATGCAAAGTTTCATCTCCTCTCTGTCAGGACTTCCCCATCATCGAGCTTATTTGGCCACTAATTTCTTCAACAGCACCGACACTCATCACTTGTAATCGTGGCCCTTGGCTCCTTCAACACTGGACTTTCTTTTCAATGGCTCCAGCTAGCAGCACGATATTGTCCTCATCTCCGTCGCCGACGCCTCGACTCTTCACTTCTCCGTGGGTCAACATCTTCTTGCAGTACAGATCAATACCATTGCACTCTTTCGTGGTCTTCGCAAACACCATTCGTGCTATCCTCGTTATAACTTGTATTTTTTAATAAAAATTTTAGGTATTTTTTATTTCCTATTTCATCAGAAGCAACAATAGAAACACGTGACCTTGAGAGCGAACGTAGTGGCTTCTTTTGTCTCCTCCGACACCATTCATGTCATCATCGTTATAACTTTTGTTTTTATTCAACATTTCAGTTATTTTTATTGGAGTTCGTATACAAATCAAATTGCTAAATTTATTAATCTACTATGAATTTTAGTTATTTTGGGATTTTATTTTGGAATACGTGTGAAATCAATTTGCAATTTTTCTTTTATTTTTTTTCCAAGTTTTAGTTCTTTTTTCTTGGAATTCGTATCCGGATCAATTGCTAATTTTTATTTAATTTTATTCCGAATTACAATTAATTAGGTATTTTACTTTGGAATTCGTATTGAAATCAAATTGCTAATTTTATTTTATTTTTATTCCAGATTTCAGTTATTTTTTTGTTGGATTTCGTATTCAAATCTAATTGCCAATTATTGTTTAATTTTTATTCCGAATTATAATTATTAGAATTTGTATCGAAATCAAATTGCTAATTTTATTTTATTTTTATTCCAATTTTCTTTTTTTTTTTGTTGGATTTCGTATACAAATCTAATTTCCAATTTGTGTTTATTTTTATTCTGAATTATAATTATTTACGGATTTTATTTAGAAATTCGTATAGAAAGCAAATTGTTTATTTTATTTTATTTTTATTCCGAATTTCAGTTTTAATGTACTTGACATGGATTTACGGATTTTATTTAGAAATTCGTATGGAAAGCAAATTGTTTATTTTATTTTATTTTTATTCCGAATTTCAGTTTTAATGTACTTGACATGGACTCTTTTACTTAGGAATTCGTATGAAAATTAAATTGCTGATTTTATTTTATTTTTTGTTTCGAATTTCAGTTTTATTGTAGTTGACATTGACTCTTTTATTTTAATCTAGACTTATAATTTGGATGTACTAAGATCGGCGGTGTAAATCTTTCTCTTTTTTCACCGATTAATGTGGGAATTTCTAGCCCTTGAGAATTATACCAAGATAATAAATGTTTATCTGTGTTTGTGGTTTATTTTAGCAATTTGGTCGATCATATCCATCACCATCAATTGAACTCATCTCAAGGTATAATTGGTTTTAGTCCTTAGCTTATCCAGGATGACCCTCCGTATTGTATAGTTAAAATGTCACCACATTCAGAAAAAGGAAAGTTCTTAAATCTAATGATTATTTTGCCGTCAGCCTGTAAAGTGCAATTCACTCATAAGCAAGAAGTATTGGTTCTAGCAACATTGAGTATTGCCCACTTCCAAGACTCGTGCTTTCACTTCATTCTTTAATCATCTGGGTGCTGCTTCTGCAGAGATGAACATGGATTGTTTTATGCACTAGATCTTGGAGGGACCAACTTCCGTGTTCTGCGAGTCCAACTTGGAGGAAGAGAGAAGCGTGTTGTCAAGCAACAGTACGAGGAAGTCTCCATTCCACCACATCTGATGGTAGGGACTTCCATGGTGAGTACTTGCTGTTCGATTTTCATGTTTCTTAGAGTTTTACATTTTCATCCATGCTTGCCTAATGTATCTTGAATTATTCATACAGGAACTATTTGATTTCATTGCTGCTGCATTGGCAAAATTTGTGGATACTGAAGGTGATGATTTCCACCTTCCAGAGGGGAGACAGAGAGAGCTGGGTTTCACCTTTTCCTTCCCAGTGAACCAAACATCAATATCATCGGGAACACTCATCAAGTGGACAAAGGGCTTTTCCATCAACGGCGCGGTAAAAGTCAAAAACACTTTGGTTCATGCCCCTCTAACCGAACACCTTCCTAGTACTTTTGTAAATGTGGCTTGAACCGGAATTTATTATAAACACATGAATTCACCCGACAAGTTTCTGTGTGAGTGTGATTTAATATGGCAATAGAGCCTCTGGTATTTGTTCCTTTTACTCGAAGGACCAGCTTTTCTGTGGCAAAATTCTGCATTGCTCTTTTAGAATGCTAGAGCAGAGCTTTTAGTGTTGAAGTTTAATAGTGGTGTGTATTAACTTTGATATCATGTTGATTCACTAATTTCTGTATTCACCTACAAACAGTCTAATACCTACCCCATTCTTATAATTTTCAATTGTTACAATACGATCAAACTGATTCGTTTATCTCAATGAAATAATTATTGGTTAGGAAGCATATTCTTGAGGTGCAGTAAGAATATTATGTTTCTCTGGTTGATGGTATCTGTGTCAATTGTTAAGCATCCTATAATAGTCACACTCACAATTACTTGATTTGGATATTTATTTGATTGCTTCTGAATCAGGTGGGCGAGGATGTTGTCTCCGAGTTGAGCAAGGCCGTGGAGAGGCAGGGGCTGGATATGAAAGTTACAGCATTGGTAAGTTAATATCTGTTAATTTATTCAATTTATACACCATCTATGCTTGGCTTTCGTATCTAAAGCTAGCTATGTTAATTTCTTTGTATCCTGATGCCGCTTTTGGTTTTTTGACCCTTTTATCAGTTGACGGGATCCAAACATGGTTAACATCATGCAAAACGTTTCTATCCGATACATTGTTTGCTTTGTAGACTGAGTACTGATGTTTTGTAGGTTAATGACACAGTCGGCACATTGGCTGGTGGGAGATATATGGATAACGATGTAGTTGCTGCCGTAATATTGGGCACTGGTACAAATGCAGCATATGTTGAGCATGCCAATGCTATTCCTAAATGGAATGGTCTACTGCCTAGATCTGGAAATATGGTTAGTGTTTGATTTCCTTCTTATGCTTGGAATAACAATGTTTGTTGAACCATTGTATGGTCTTACACATGCACTGTACCTTCACCGTTGTATGGTATTCACCCCACTTAAAATATTAACCTTCGAATTATTTGGTATTCTCTAGGTAATCAACACGGAATGGGGGAGCTTTAAATCAGACAAACTTCCTCTTTCAGAATTTGATAAAGCCATGGATTTTGAAAGTTTGAATCCTGGAGAGCAGGTATTGTCTCCCTGGTCTTTTCCTTCCCCTTTTGTTTTTAGGTGAACTGCATTAATATATGTTAAAAACTTATGTATGCCAGATATATGAAAAGATGATTTCTGGAATGTATCTGGGAGAGATTGTGCGAAGAATCTTACTGAAACTGGCTCATAATGCTTCTTTGTTTGGGGATGTCGTTCCTCCTAAGCTGGAGCAGCCATTTGTACTGAGGTATTCTTTCTTGATCCATTGGTCATCGAGCTTTTCATGGCTGCTTTCTGACATGGCAAAAGCTGTTCTACTTTTTCGTTAACTAACAAGCTGATCAAGAATTTTCACAAATTTCTATGATAATCTGCCTTGGATAAGAATGTGCATGACATAAAGAGTTTGGCTTCTTTTACCTGATAGTATTTAAGTTGGCGACATTAATAGTCGTAGCTGAATAAATTGCAGACAGGGAAGTTGTGTGTCTTCTGAAGTTATTATCATGTTACATCAGTTGACTAATCCATGGAAAGTGACAGGTAAAAAAAAAAAAACGGATTTGTCCTCTTTATTGACAAAATATTTTCTTCTGGACAACTCTCAGTTTTGTCACAACTCGGTGCAACCCGATGCTTGGCCCCTTTTCTTCTCTGATGTATTAGCATAACTTCCTGGAAAGTGACAGATAATTTTTTTATGCACAACTTAATATTATAATGATGTGGCATGGCCTTCGGTGGATTGCAAGCGGAGGAAGAAGATGATTACAAGATCGCGGATTAGAACGTAAAGGGTAACGATGGATAGGGCAATCGCCTTCCTGGTCACCAGACGCCGTGACAGGAAGCTCTGAAAGCTATGTACTAAATTTTACTTGTATTGATTCTCAATGATCCTCAAACGATTACAAAGCATCCTCATATATAGAGAAGGCTGAACTCTCCTTAAGGGATTCGAAGTCTACTCACTCCTTTCCTAATATCAATAACTTACCTAATTACGCAACGTCCTGAAGGGGCAATCAATCTCCGAACCTACCATAACAAAAGGGGTCTCCGAACTGATCTTGTATAGCATCAAACGTCCTGCTGTTCGGCGTCAGGAGAACCTGCCGAAAGAGCTTGACGTGTTTGTTTGGACCTCCTCTGATATGTCTTGCCATAAAAAGAGTCCACAACATATAACTGAGTACTAAAGGTAAAGTTAAGGTTGATTTTTTTCCTTTAGTCAAATGGCTCATAAAATCTGAGCAGTGTCCTAATGAGAAAAATAAAGGTCAACTTAAGGGTTTTTTTTTTCCTTTTAGCAAAATGGCTCATAAAATCTGAGCAGTGTCCTAATGAGAAAAATAAAGGTCAACTTAAGGGTTTTTTTTTTCCTTTTAGCAAAATGGCTCATAAAATCTGAGCAGTGTCCTAATAAAAATAATGTTCCTTTCTTCTCCTTTAGTGGGAATTAAGTAATAAATAGGTTATTAATAGATAGATGCATTTGGTAGTATAGCATACTTGAGACCTGGTGAAGAACCAGTGCCATTAGTAACAACGAATCTGTGAGTTGTACAAATGGTCTCTCACACCAGCTCACAAGAAAGTATTTATTTGTAGAAACTCTGGTACTTTTTTATTCTTATTCTGAGTCAATCCAACTTCTGAATTTTATATAGGACGCCAGATATGTCAGCCATGCATCATGACTCGTCACATGACCTTAAAATTCTGGGAGCTAAGCTGAAGGATATCGTGGGGGTATGACTTGCTTGAGCCAAAGTGGATCCCAAAAATGTTCAATAACCATTGATTGCTTATGTACTATTTGATCTTATGTTCAGGTCCCAGATACTTCCTTGGAAGTAAGATACATTACTCGTCACATCTGCGACATTGTTGCAGAGCGCGGTGCACGCTTGGCTGCTGCTGGTATATATGGCATCCTGAAGAAGCTAGGCCGGGACAAAGTGCCAAGTGATGGCAGTAAAATGCCGAGGACAGTCATTGCCTTGGATGGTGGACTCTATGAACATTACAAGAAGTTCAGCAGTTGCTTAGAAGCAACTCTTACGGACCTCCTGGGGGAGGATCCCTCGTGTTCGGTGGTTGCCAAGCTGGCCAACGATGGCTCTGGCATTGGAGCTGCTCTCCTTGCCGCCTCGCACTCACAGTATGCCGAGGTCCACTAGTCTTGAGAATGAGCCCCATGGATCAAATTCTGAGTGTAGCTTCTCATTTTTCCCTTCAAAACTTACTAATATTCTGGGCTCCCCTCGTGGAATTCTATCTTCTTTGGCTATTCTGCAGACACATTTCAGTGACTGCAGCGGGATATAGCTAGTATAGCGCCAATGAGTTCGGAGGTTATCTAATTGCATAAACGTTTGGATGGCAGGAACGTGGAAATGGATGTTTGAACGAAGCCATTCCTGTTCCGCCCATTCTTTTTCCAGTTGAATGGTGTTGGAAACGAAGCAATTTTTTTTATATACTTTTGTATTTTCTCGTCGCAATAAAGAGGGGGACGTGATGAATTTAACTATCTGTTCTCTCAGCTTGATTTCTTCAAAATATGACTGAATGCACGTTTATGGCTTCAAAATGATATAC

>PeHXK6

GCGGGAGGCGGAGGAGGAGAGGCGGAGGAAGGCGGCCCCAGTGATTGAGGAGGTGGAGCAGAGGTTCTCCACGCCGACGGCGCTGCTGCGCGGCATCGCGGACGCCATGGTGGAGGAGATGGCGCGCGGACTGCGAGCCGACCCCCACGCCCCGCTCAAGATGCTCATAAGCTACGTCGACAACCTCCCCACTGGGTAAGGCCTCCACTGCTTCTGCGATTTTTTTTCGTCTTTTGGGGCGTGCATGTGTGTTACCCGAGTGACACGCTTGTTCTTGTGCGCGGGCGCGTGGGTCTTGGGGGTGAATTGGTTGCAAATTTTGGCGTGGCTTGTTGATGTGTGGATTCGGTAGAAATGTTGCATTGTTCCACTGCTGAGGTGGAGGTGCCGTCGTGAATCGTGGTATTGGGAACACCGTGCCTTCAGGTGGTTTGAGCGAAATTTGGTTTGGGGGGGGGGGTGTTATTTGATCTTGCTATGGTCCTTAACTCCTTATTGATTCTGGAAGAAGGATGATAACTTTTGTTTTCCCTGTGTGGCCTTTTGGTTTTGTGGGCACCTTCGCATTTTGTGTGATATCTTTGACTTGACGCCTCAATTCCAACTTACACGATATAGGATGTCTGCACAAACAAACACGGTGTGGGTCAGGACATCAGGAGTAAAATAAGAATTTTTACAAGCTTTTCAGTATAGCGTTTGGTCTGCAATTTTTGGTTAAACAGTACGGTTTGTATGTTTGTCATAGATGATAGATCAACCTATATCTTCCCTAATTTGTGACGACTGCAGAGAGATGTGTGTCCACATGACCTCTCTAGTTATCTTAATAAATGGACCATTTGTATTTTATACCTATCTACATGTCGGGCTTGTTAGAATACGAGAACCCTTTTGTATTTCCTTATCTGTATATGGTATACCCCCTTGTAATCCCTATATATATTGCCCATTGGGTCTCATGAATATACATGCAATATTCCTAACATGGTGTCAGAGCTAGGGCTCCTTCCACCGCCACCCGACGCCGCCGCAGCCTGCACGGTCGCTGCTTGTGCCGCCTGCACTCGCCGCAGCACGCGCTTTGGCCGGCCGAAGACGCCGCCGCCGCGAACCCGACGCACCCCCTGTGTCGTCTGCGCCCGCCGCGACGACACCGCCGCTTGGGCCGCCACCGCCGCTGACGCTAGCTTCGGCCGGCCGAAGACGCCGCCGCTGTTAGAGCCGACCGGGAGTCCGACGCGCCCCCTGTGCCGTCTGTGCCCGCCGTGTGTGCCACTTGCACTTGCTGAAGCACTGCTGTTGCCACCGCCGCTCGAGCCGTCGTTGCCGCCGCCGACGCCGACACTCCGGCCGCCGCCGACACTCGGACCCGCCACTGACGCCACAAGCAAGGGGAACCCTAGTTCCCCCCGCACGCCCCTTCGTGCTTCGCAAGCCTTATTAGCGTGAGCCGGTTCGGCCCGCAAGAAAAGTAAAAAAAAATCAAAAAGAAAAAGAAAGAAAAAAAAAATAGAAACGTTGATCTAACCGCCGCCTATTAGACTTCGTTGCCTTGGTCGCCACGCCGTCCAGCGCCGCCTACCGATGGCTTCCGTTCCATCAGCTGCGATCTCATTCTCGGGAACCAAGCTTAATGGCACGAACTACAGGTTGTGGTCTTCACTCATGGAGATCACGCTCGATGGTCTCCGCCTCTGGGGACATGTTATTGGTGCCTAACCTTGTCCGTTGTTGCCGGTTCAGCTTTCTCCCAGACATCCTACTTCTGCACCTCCTGCAGAAGATGAGGCCGCCAAGAAGGAGGCAGAGGCCGCTATTGCCGCTGCTGATGCTGCTGATGAGGCCTTTCAGTATGCCATAGACGCTTTTGAGCGCTGGTGTGCTGATGAGGCCCGTGTTCGAGCCAGCCTGGTGGTCAGCGTTGACGACTCCATCCAGCTAGACCTTGTTGGTCTGCGTGCCACTTGTCATATCTGGGAGTACTTGCGCAAGCGATACCAGCAGAGCGGCGATGCTCAGTTCTTTTCGCTTCAGCGTGAGTTGGAGGCGCTTTGCCACGAGGACTCCTCCGTCGACGACTTCTTCCAAGCCCGGGCCAGTCTCTGGCGCTAGCTGGACGAGCTGGCTTCTCCGCACTGCCAGGAGCAGGGATCAAAAAACCGCCGGTAATCGGGCCGGTTACCGGTACCGGAGGGTGGCCGGGAGGAGGAATCCCGGCGTTTTTTTCAATTTTCGAATGAGTTTTAATTGAATTTAAATGAATTTCACCGAATTTGACCGGTAACCGGGCCGGTTATCGGTACCGGAGGGGGGTCGGGATTCAAACCCGGTCCGGTTAGCTGACCCTTGGCCAAGAGTGTCATAAAGTGCAGAAGGCGGATCGTGATCGCCTTCGCCTTTATGACTTCGTCTGCAGTCTTCGCCCAGAGTTTGAGCCTGTCAGGTCTTAACTACTGAGCTGCTCACCATGACCGACAGTCACTGAGGCTCTTTCGACGCTTCGTGTTGAGGAGCTTTGCCATGGGTTAGTAGCACAGTTTGTTGCCAGCTATGCTTGCTACCCCCCCTTCGACTACACTGACGGTTTCTGCTTCGACTACAGTCTCTCCTCAGGTGTCTGTTTTTGCTCCTGTTTTTGCACCAACTGGGGTTCGGTGCAATTATTGCAAAAACTTCGGGCACGACTACCGGCGTTGTGCGGTGCGAAAAAAGCAGAACAAGGCGCGTGGACGATCCAAGGGGGGCAAGGAGGCCCTAACTCCTCGATGGGAGGACAAGCCAGCACGTCAGGACCTTCTCAGCGAGGTGACTTTGTTCAGCAGTTCTGCCAAGCTCTACTTTAGCTGCTCTTTCACAGCTCAGTAGGTTCTGCCAGTCTAGCTCAGACGTCTCCTCCAGGTACTTCTGACACCGGACTCCACATCTCTCACTCCAGTAGGACCTCCTATGTTTCCTATCTCTGTTCAATCAGCTGACGGTACTCCTTTGTCTGTCAGCTGTTATGACATTCTTTTGTCCACTGACTTTTCTGTTCCAGCTGTTTCCCATGTTCCCCAGTGTCGTTGCAAAAAGACTAGACAGTGGGTATAGGGTACCCTTTGTCTTGGAAGAGGGCCGAAGGGAGGAGCCAAAGACATAACTATATGATTGTATTTCCTTGAGTGTGAATAAGAGGAAGCCCCCCTTGTACATGCCCTTGGGGCCTTATTTATAGGAGGGCAAGAAGGGACATTTACCTTCTTGCCCCTCGCAATGTATTGAGAGAGAGGGTTTTTAGGTACATTTGTCTACAGTACACCATATGGACTTGGTCTACACATGTGCAGTATCCCTGATGGTACACTGGTATTCCTGATGACCCTAGGGTGCGAGGCACCTCTTGGTGCCTAGCTACGGCAACTCCCTCGTCTGAGTGGTCCGAGGTCGCGAGGACTGGTCCCGCGATCCCAAGGGGCGAGTGGACGCAATCTCGTCGCCCTGGCCCCTCGGGAGTCGGGATACCCCGGAAGAAATCGATACGAACCCTCGAGGCAGGCGGCGTGTCGGGGCTGCCCCGCCGAGGCGATATCCTTGAGGCGGGCGGTGAGCAGCGGTAGAGCCCGTGGGGGCGAAGTCGATGTGGAGCCGCCACGCCGAGGCATGGACCTCGAGGCGGGCGGTGAGCAACGGCGGAGCCTGTGGGGGCGAAGTCGACGCGGAGCCGCCACGTCGAGGCATGGACCTCGAGGCAGGCGGCGCGTCGGGGCCGCCCCCCCGAGGCGATAGCCTCGAGGTGGGCAGTGAGCAGCGGCGGAGCCCGTGGGGGCGAAGTCGACGCGGAGCCACCACGCTGAGGCATGGACCTCGAGGCGGGAGGCGCGTCGGGGCCGCCCCGCCGAGGCGATAGCCTCGAGGCGGGCGATGAGTAGCGGCGGAGCCCGTGGGGGCGAAGTCGATGCGGAGCCGCCACGCCGAGGCATGGACCTTCTTGTTCGGTCCCCACGAACAGCGCCAACTGTTGTAGCAAAACGACTAGACAGTGGGTACATGGTACCCTTTGTCTTGGAAGAGGACCGAAGGGAGGAGCCAAAGACATAACTGTATGATTGTATTTCCGTGAGTGTGAATAAGAGGAAGCCCTCCTTGTACATGCCCTTGGGGCCTTATTTATAGGAGGGCAAGAAGGGACATTTACCTTTATGCCCTCACAATGTATTAAGCGAGAGAGGGTTTTTAGGTACATTTGTCTACAGTACACCATATGGACTTGGTCTACATGTGCAGTATCCATGATGGTACACTGGTATTCCTGACGACCCTAGGGTGCGGGACACCCCTTGGTGCCCGGCTACGACACCCAGCTTACCATGCAGCTTCTCTTGGTGGGTCAGCTCACCGATCTTGGCTGTAGGGTTGGTTTTGACTCTCACATCTGTTTTATCCAGAATCGACAGACAGAGACGGTACTTGGGACTGGTCGGCGCCGTAGGGATCATCAGGGTCTCTACATCTTTGATCGCCTCCACCTTCCGTCCATTTCTGTTGCTCCTGTCACCTCTACGTCTACCTCCACCGCCACTGCTGCCACTACCTCTGGAGTTTTTGCGCAGTGGCATTACCGTTTAGGTCACTTGTGTGGGTCCCGTCTGTCTTCATTAGTTCGTCAGGGTGTTCTTGGACGTGTTACTGTTAACACTAGGGATCAAAAAACCGCCGGTAACCGGCCGGTAACCGGGCCGGTTACTGGTAGCGGAGGGTGGCCGGGAGGAGGAATCCCGGCGGTTTTTTCAATTTTCGAATGAATTTGAATTGAATTTAAATGAATTTCACCGAATTTGACCGGTAACCGGCCGGTAACCGGGTTGGTTACCAGTACCGGAGGGGGGCCGGGATTCAAACCCGGTCCAGTTAGCTGACCCTTGGTTAACACGTCCATGTTTTGTTAGGGTTGTAAGCTGGGCAAGTAGCTACAACTTCCTTAACATTCTAGTGAGTTCTTGTCTTCTCGTCTTTTGACTTAGTTCATTCAGATGTATGGGGACATGTTCCTATTATCTCTAAAGGCGGGCGTAAGTTTTATGTGATCTTCATGGATGATTATTCCCGCTTTACATGGCTTTTTCCTATGTCTAGTCGTGGTCAGTTTCTTGCGATCTATCGTAGCTTTGCCACGATGGTTCGTACTCAGTTTGATTCTTCCATTCGTACTTTTCGCTGTGATTCTGCTGGCGAGTACTGTTCTGACACTTTTAAACAGTTTCTTTCTGAGCATGGCACTCTTTACCAGTCTTCTTGTCCTTGTGTTCATACTCAGAATGGGGTTGCTGAGTGCAAACATCGTCATCTGATTGAGACTGCCCGCACTCTTCTCCTTGCCTCTCACGTTCCACCACAGTTTTGGACTGAAGCTGTTTCTATGGCTGCATATCTCATCAATATGCAGCCTTCCACTGCTCTTAAGGATGTTAGTCCTATAGAGCGTCTCTACTCTCGAGCCCCCCAGTACACTCACCTTCATGTTTTGGCTGCACCTGTTTTGTTTTGCTGCAGCCTCATGAGCGCATCAAACTATCGGCTCAGTCAGTTGAGTGTGTGTTTCTTGGTTATAGTTCTGCGCATAAGGGTTATCGCTGCTATGATCTTTCTGCTCGACGGATGAGGATCTCACGAGACGTTGTTTTTGACGAATCTTGACATTTCTTTTATCGAGATTCGGCTCCTTGTCTTGTTTCCACCGTTGAGACGATTTCCTGGTTGTCTCTTTCTGACTTCTCGTCCTTTTCTCCACTATCGCAGTTTTCCCTTTGTCTGTCTCGAGTGATTCCTTCTGTACCTTCGCCTCCGGTTCTGCCTCCTGCAGCACCGTCTCTTGTTCCTACAACACCACCGTCTCGTTTTTCTGGTATTTACTACTCCAGGTGTTCTTGTCCTCCACCAGTCTCCTCTCTTGATATCTTGCTGATACCTACATCTGATCCTCCTGATGATGCTCCTCGTTACGCCCTTCGTGATCGGACATCCCTTCGACCTCCTGATCGTCTTGGATTTGCTCCGATAGCTCATGATCTCTCTGTCACCGAGCCTAGTTCTTATCGTGAGGTTGCTCCTCACTCTGAGTGGTAGCTTGCTATGGCAGAGGAGCTTGATGCTCTTCGACGTACGGAGACTTGGGATATTGTAACGACACGTGTGCATGCTCGCCCTATCACGTGCAAGTGGGTGTACAAGATTAAGACCCGGTTTGATGGTACTATCGAGCGCTACGAGGCTCGTCTTGTAGCTTGCGGCTTTCAGTAGGAGTACGGTCGTGACTACGATGAGAACTTTGCCCCTGTTGCCCATATGACCACTGTTCGCACTCTTGTTGCGGTTGCTTCTGTTTGCCGGTGGTTTATCTTTCAGTTGGATGTTAAGAATACCTTTCTTAATGGTGTTTTGCATAAGGAGGTCTACATGCAGCCACTACTTGGGTATTCTGTTCCCGATGGGTACGTCTGTCGTCTTCGTCATGCTCTTTATAACCTCAAACAGGCTCCTTGTGCCTGGTTTGCGTGTTTCACCTCAGTGTTTACTGCTGTCGGTTTCACTGTCGGTTTCCATGATCCTACTCTTTTTTCCCATGTCTCATCTCGGGGTAGGACACTGCTTCTTCTGTATGTTGATGATATGCTCATCACTGGTGATGATTCCTCCTTCATCACATATGTGAAGAAGCATCTCAGTGAGTAGTTTTTGATGACTGATCTTGGTTCTCTTACGTACTTTCTTGGGATTGATGTCTCTTTCACGCCTGAGGGCTACTACCTCTCCCAGCAGAAGTATGTCCAGGATCTTCTTGATCGGTCTGGTCTGACTGACACTCGCACTGCTGAGACTCTTATGGAGCTATCTCTACAGCTTTGTGCCACTAATTGTGATCTTCTTGAGGATCATACTCGATATCGCCATCTTGTTAGGAGTTTGGTGTACCTTGTAAGTACTCGTCCTGATATTGCTTATGTTGTTCATATCTTAAGTCAGTTCATGGCTCGTCCTACCCACCTTCACTACAGTCACCTTCTTCGTGTTCTTCGCTACTTGCGTGGGACTATCTCTCGTCGTTTGTTTTTCCCTAGCTCCACCTCATTACAGCTTCAGGCGTACTCTGATGCGACCTGGGCTAGTGATCATTCTGATCGTCGTTCTCTTTCTGCTTACTGTGTTTTTCTTGGCTTCTCCCTGATTGCTTGGAAGACAAAGAAGCAGATTGCAGTTTCCCGTTCGAGTGCAGAGGTTGAGTTGAGAGCTATGGCAGCAGTGACAACAGAGGTTACTTGGTTGAGGTGGTTACTTGCAGATTTTGGAGTGCCTGTGATGAGACTTATGACTATTTTGTCTGACAGTATAAGTGCTATCAATATTGCCCATGACCCAGTGAAGCATGAGCTTACCAAATATATTGGTGTGGATGTTTTATACACTCACTCACAAGTGCAGGACGGAGCACTTGCTTTACAGTTTGTGCTATCAGAGTTACAGATTGCTGACTTCTTCACGAAGCCACAGACTAGGGTACAACATTTGTATTTTCGCTCCAAACTCAGTGTGTGTGATCCACCATGAGTTTGAGGGGGTGTTAGAATAGGAGAGTCCTTTTGTATTTCCTTATCTGTATATGACATACCCCCTTGTAATCCCTACATATATTCTCCGTTGGGCCTCATGAATATACATGCAATATTCCTAACAGGGCTTACCTTTAAGATCAAGGTACTATTGTTTATACGTCGCCTGCTTCTTTTTTTGATGGTATGGTCAAATTGTTGTATGGCTAATTTCCAAATAAGCACTCGCTATTAGTATTATGTTATACTCCTCCGTTCTCGAATACTTGGCAGTTGGCACTATTGAAAACATGCAATCGAACTTTCCAATCTTTGAACAATATTATTTTCTAATTATCTAAATTAGTCACATGAAAGTGGTATTGCTATATTTGTCATAAAATTACTCTGATAATATTATACTTACTGTTTACAAATATATTTGAGGGTGTGAAAATTCAAAGTGACAAGTATTCGATAATAGAGGTAATATTATGAAAATCCCCCCAACGTGTTGAGTGAAAGAAATGGTTGAAGACTTGAAAAAAGTTTCAGCCATGAGTTTGTTAACTGTAAATGTGTAACCCATATACGTGTCTGAAAAAGAAACATGTCTATCACTTGATGAAATCTACTTACATTGTTTCCTCTGCTATTATTTTGCTGTTTACTGATTTTTATGTCACGTCTGCAGGGATGAGCATGGATTGTTTTATGCACTGGATCTTGGCGGGACCAACTTCCGTGTTATACGGGTTCAGCTTGGTGGAAGGGAGAAGCGTGTTATCATGCAACAATATGATGAAGTGTCCATTCCACCTCATCTGATGGTTGGGACTTCCACAGTAAGTGCTTTCAATTGCTCTTTTAACTCTCCTGCATCAGCAACATTTTCTTGCATGATTTACTCACATTTCACAAAGCACGAGTACAGGAATTGTTTGATTTCATTGCGGCTGAGTTGGAAAAATTTGTCGAGACTGAAGGAGAAGATTTCCACTTGCCAGAGGGCAGGCAGAGAGAACTGGGTTTCACCTTTTCTTTCCCAGTGCACCAAACATCAATATCGTCAGGCACTCTCATTAAGTGGACAAAGGGATTTTCCATCAATGGCACGGTAAAAATCAAATGACCTTAGTTCATACTTCGCTGACTGAATGATATACTAATATTTCTATTACCTTGATGATCAAGGCTTGATCAGAAGCCGAATTTCTTATCAACAAATGATTCTCCCTATGCAATTTCCCTAATCATCTTAAATGTGGCAATTAAACTTCCCACATTTCATTTTCCTGAGAACACTTATTAAGTGGACTAAGGACTAGCTTTCAGTGATGTCATGATGCGTTCTTTTGATTTTTGGGAACTCCCTGCACTGACAAGTGGCGAACCGCTGTCCTGCATTGTTCTTTCATGATATTAGAACTGCTTTTAGTGGAAAAAATAGAATGTTAGTATGAACTGTTTTTGTTTTGATTTTGAACATGTGTGGATTTTAGGTCATATATATTTTTTGGCTTAACCTAATTTAGGCATGTTTAGAAAGCTTACTCTTGAAGTGATAAAAAGGATCCATGTTTCTTAGGATTTGTGGTATTTGTGTGAATTGTCAAGTATAGCTAGCCACAAATCCCGCACAATTGCGTGTCTAGATGCTACAATCCATAACTTTATATTTAAAAGTAGAGTTGTATTTATTATGCAACTCTAAAATCTATAGCTGAAAGTAGAGTTGTATTTGTTTTATAAGTGTAATAGCCCTAATAAGTTATAACTATATTTGATTATCTGCACTTTTATAATATGGACCGACCAAAATAAGTGGTGTATATCTTTCTCTTTTTCCTCCTGTTAACATGGGAATTTCTAGCCTCTCAGAACAAACATAGTGGCAACTTTTAAGTTTTAACGTCGTCTTAATCATATAATAATAAATGACTATAGTGTTGATTTGGATTATTGATTGCTGTGAGCTCAGGTCGGGGAAGATGTTGTGGCTGAATTGAGCAGGGCTGTGGAGAGGCAGGGTCTTGATATGAAAGTTACAGCTTTGGTAAGTTCATTTGTTTGTTAAATTATTTAGCATCTTAAGCATTCCCATGCCTGGCTTTTATATCAAAGGCATTGGCATTTCCTATGCACTCTGATGCTGGTTTTGGATTTTTGAGATGCCATATGTTGATGGGGTGCAAAGGTTGGACGCATATTGTATCGGCAAATACTTTTGGTGTTATTTAAACTGACAACTAATTTTTCTTTCGTAGGTTAATGACACTGTAGGTACATTGGCTGGTGGGAGATATGTTGATAATGATGTCGTTGCTGCTGTAATATTGGGCACTGGCACAAATGCAGCATACGTGGAGCATGCAAATGCAATTCCAAAATGGAATGGGCTACTACCTAGATCAGGAAATATGGTTAGTGTCTGACTTCCTTTGTATGCTTGGAATATTAAAGGTCTTTGGGCCTTGTCTAATTGTATACATCCATTGCAACTGTTGTGTTTGTTAGAATAGGACTCTATTTGTATTTCTTTATCTGTATATGGCAACCCCTTTGTACTCCCTATATATATTGCCCATTGGGCTCTCTTAAATATACACACAATATTCATAACATGGTACCAGAGCGGCAGCTGCCGACGGTGCATCGCGTCGTGGTCGTTCGCTCGGCTTCCTCCCCAAGGATCCCGAGGCAGAGAAGATCCCCCGCGGGATGAGGTGGCGTCGGCGATGGCGAGGCGTTGTCGACGAGGAGGAGGCCCGCCAAGCGACGGCGGCGCGCATCCGGGAGGCAGCGACCGTGGAAGCCATCGACGGCATCGAGGGCGGCTCGAGTGGCGATCCGAGAGGAGAGGAGGACCCAGAGGCTGCCCCGGGTGGCGCGGTGGCGGCCGGATCCGGGCTTCCTCGACGTTCTCGACCAGGCTCGGGGGCAGGGTCGCACGGAGCAGAGAAGGCGTCGGCGCGGGGTCGCACGGGAGCAGAGGAGGCGTCGGCGCACGAGCTCCATGCGCTCATGTGACTCGAGCTCTGGCTCGACTTCCCCCTCCTCCCGACGACGACAAAGGCGGCAGCTGCGACCAAGGGAGGCGGCTGCTGCTCGAAGAAGGCGGCAGATCCGGAAGGAAATCGGGGACCGCACAGCTCGGGAGCGGCGTAGAGAAGGATATGAGATATGGACAAAAAGAGTAGGATCATGGGAACTGATAGTGAAACCTGATAGCGCGAATAATACCTTGTGTATTCAACTGAGAAAACAGGAGTACAATATATAGACTCTACCGGTAATCTATCTCTACCTGATTTATAGCAACAAGATCAGGGAGAAGATCCGGGAGTCCAGGCGCATAAGGAAAAGCTAGGCATGGAAAGAGTCAACAGCCAGCCACCTAAATATAGAGGGGATCACCTAAACAGGGAAAGAGATAAACTCTAACACCCCCCCGCAGTCGGAACGTCCATAGGACAGACGTTAAGACTGGAGCGAAACTCAGAAAAAACAGAAGTGGGTAGACCCTTGGTGAAGATGTCAGCAAACTGGGATGTTGTCGGGACATGGAGAACGCGGACATCTCCAATAGCAACATGCTCGCGGACGAAGTGGAGATCAATCTCGACATGCTTTGTGCGCTGATGCTGAACAGGGTTGGTTGATAAGTAGACAGCACTGACATTATCACAATAGACCAAGGTACTCTTCGTCAGGGGACTGTGGAGTTCCTGCAACAACTGTCGAAGCCAGCAAGCCTCGGCCACGCCATTGGCGACCGCCCGGTACTCAGCCTCGGCACTGGAGCGGGATACTGTGTTCTGCCGCTTGGAGGACCAGGAAATTAGGGTGTCACCAAGGAACACAGCATAGCCTGAAGTAGACTTGCGTGTGTCCGGACAACCAGCCCAATCAGCATCAGTGTAGACAAGGAGAGCAGAGGTCGTCGATGGTCGCAGAAGAAGGCCATGATCATTTGTACCATGCAGATAGCGAAGGATGCGTTTAACTGCGGAGAGATGAACGTCACGAGGATCATGCATGTGGAGACACACCTGCTGAACAGCATAAGATATATCGGGCCGAGTGAAGGTGAGGTACTGCAAGGCACCAACCAAACTGCGGTACTGAGTGGGGTCGCTGACAGGCGCACCGGCTGTGGAGGAGACCTTGGCATGAGTGTCGACAGGTGTGCTGCAGGGCTTGCAGTTAGTCATGCCAGCACGCTCAAGCACATCCAGAGTGTACTGCCGTTGTGAAAGAAAGAGGCCTTCAGGATGCTGCTGGACAGCCACACCCAGGAAGTGATGGAGGACACCGAGATCCTTCATGGAGAATTCCTTCTGAAGTGCAGTGATGATGTGCCGAAGGAGAATCTGGCTGGATGCAGTGAGGACAATGTCATCCACATATAGCAGTAAGTATGCCTTGTCGGTCCCGTGTCGATAGATGAACAAGGAGGTGTCAGATTTGGCCTCAGTGAATCCCATAGTGAGCAGATGCGCAGCAAAGCGACTGTACCAGGCACGAGGCGCTTGCTTCAGACCATACAGGGATTTATTGAGACGACAAACAGCATGTGGCTGAGTAGGATCAACAAAACCTGTGGGCTGTGAGCAGTATACCGTCTCAGTGAGAGTGCCATGTAGGAAGGCATTCTTGACGTCGAGCTGGTGAACCGGCCAGTGTTGAGAAAGCGCCAGAGACAGAACAGTACGAACAGTCGCAGGTTTGACCACCGGACTGAAAGTCTCGTCATAGTCCACACCGGGGCGCTGGGTGAAGCCACGGAGGACCCAACGGGCCTTATATCTGTCGAGGGAACCATCGGCATGAAATTTGTGGCGGAAAATCCATTTGCCGGTGATGATGTTGGCGCCGGGAGGACGCGGGATGAGATCCCACGTGTTGTTGGAGAGCAAGGCGCCGTACTCTTCGTCCATCGCGCGTCGCCAGTGTGGATCGGCGAGGGCGCTGCGATAGGTCTTCGGAATAGGTGATAGGGCCGTGGCACTCAGTGCGACTGGCAGACGAAAGCCAGACTTGCCGCGGGTGGCCATCCCGTGCTGGTTAACCACCGGGGTGATGGGGACAGCACCAGATGGAAGCCGAGCAGGTGCAGGAGCTACGGGAGCCGCCGGGATAGGCGCAGTAGTGACAGGGCGCGGTCGGCGGCGGTACACTAGCACGGGCGCAGTGGGTGCCAGCGCCTTCGGGGGTGATGGTGATGCTGCGCGGGGCGCAGGTGACGGGGCCGCGCGTGGCGCGGGAGCCGTCGGGGCCGCGCGTGGCGCGACGGGTGAGGGTGATTCCGTAGTAGCCGTAGCGGGGGCGAGAGCCGTCGGGGCTGCGCGTGGCGCGACGGGTGAGGGTGATTCCGTGGCCGCGCGTGGCGCGGGTGCGATGGGCAGGTGCGGCAGTAGATGCTGCAGGCCCGGGGCGTCAGCAGCCGTAGCGGGGGCGACGTTGGTCGCAGCAACCGCAGCGGGCGAGGCGTCAGGCGAAGCGAGGCGAGTACCTGCAGGAGAGGAGGGACCGATCGGAAAGACCGAAACCTCAAACTCAGACAAAAAATCAAGGTCAGTGGGCGATGTGCGAGAGCCAGCGAGGGGAAAGCATGTCTCATCAAAGACCACATGGCGAGAAGTGATGATTCTGTTGGACGAGAGGTCCAAGCAGCGGTAACCCTTGTGGTTGCTGGAGTAGCCAAGAAAGACGCAAAGACTCGACCGGGGAGCAAGCTTGTGGGGTGCGGTGGCGGACATGTTGGGGTAACATGCGCAGCCGAACACACGGAGGTGGTCGTAGGAGGGATGGGAACCAAAAAGGGCAAAGTGGGGGGTGCCGAGTGCTAGTGTTTTGGTGGGTAGCCGATTGATGAGATATGTGGCGGTGTGAAGACCTTCGACCCAATATGACGGGGCAAGGGAGGCCTGAAAGAGCATGGAACGTATGATGTTATTAACAGAACGAATCATACGTTCGGCTTTACCGTTTTGAGAGGATGTGTAGGGGCACGACATCCGGAGCAGGACGCCATTGGTGAGAAAGAAGGCGCGAGTTGAGGAGTTATCAAACTCACGGCCGTTGTCACACTGAATGCTCCGAATGGTGCAACCGAACTGCGTCTTCACATAAGAGAAAAAATTGGCCAAGGTGGGAAACGTTTCGGATTTTAGCCGAAGCGGGAACGTCCAAAGGAAGTGCGAGCAGTCATCAAGAATAACAAGATAATATTTGAATCCGGACACACTGGTAACAGGGGAGGTCCACAAATCACAATGAATCAAATCAAAATTCTTAATGGCTCGCGAGGAGGAGGTTCCAAAAGGTAATCTAATGTGACGCCCAACTTGGCATGCATGGCAAAGAGAATCACTAGTGTTTTTATTCCAACGGATGGCGGAGGTACTAGCGAGGGTGGACAGTGATTCATGCCCAGGATGTCCAAGACGACGATGCCAAGTGGCGAAGGGGGCCGCTGCAACAAGGGCGCAGGACTCCGTGGAATGCACTGGTGCGGTGAGAGTGTAGAGGGGCCCGGAACTATTGCACCTGGCGATCACGCTCCGGGAATTGAGATCCTTCACAGAGAGGCCAAACGGGTCAAATTCGACAGAACAATTATTATCAATAGTGAACTGACGAACAGAAATGAGGTTTTTAATGATATCAGGGGCAACCAGGATATTATTGAGATAAAAGGGACCCGGGAGGATAGAGTGGCCAGATGCTGTGATAGGTAAAGTGGAACCATTGCCAACTACAATGGAGGAGGAGTGTGGTGGGCGAAACAAGGAGATGTTACCAGTGTCGGAAGTCATATGACTGGAGGCGCCGGAGTCCATGACCCAATCAGTTGCACCGGGAGGATTGAGGGTCAGGGTGCTGAAGTTGCTGGCGAGGGACGGCTGATCCCAAGAACCGGACCACGGTGTCCACGGTGAGGCAGGGTCAGCCGAGGCAGCAGGCACCGTGGGGGGGTGCGGACCAGCCTGCCCGAACGGCGTCGTCAGGAATGGCCCAGGGGCGAACTGGGCGAAGTGGGCAGGCGGAGGCGCGTAGGGGGCGGGAGCAGCCAGCAGAGCTTGCTGGGACGTCGCCTGGGGCGTGGGCGCTCGCGGAGCCTGGCCACCGCGCTGCTGCGGACCGGGCCACATGGTGATTTGCCCGGTCCACGGGTTGTAGATGGAGGGCCATGGTGCCGGGGCCTTGCCGCCGCCGCCGTTGCCACGCCGGCGCCGGCGATTTCCACCGGCAGAGCCGCCGTTGGTGCTCCGGGAGGATCCGCCATGGGGCGCCGCCGAGCCACCAGTGAAGGGGGAGCCGGGGGGGGGGCAGAGCTGCCGGAAGGCCGCGCCGGAGCAGCGACGAGGGCGGTCGCTGGAGGTGATGTTGGTGCGCTGGAGTCGATCTCCTCGAGGATGAGATCGTTGCGGACCTCATTGAAAGAGGGGAACGGCTTGGAGCGCTTGAAGTGGGCCTTCATGTGGGCGAATTTTTCGTTGAGACCGCGCAGCACATTGAGGACAAGGGTGCGGTCGCGGATGACCTCGCCGAGGTCGCCGAGAGCGTCGGCCATCCCCTTCATCTTGCGGCAGTAGTCGTCAATCGAGAGGTCGCCCTGCAAGAAGACACGAAATTCGGCGTCGAGGCGAAGGGCGCGAGCTTCGCGGTTGCCGAGGAATTGCGCCTCGATGATGGTCCAGGCGCGGCGTGCAGTGCCGTTGCGGGTGCGGGCGGACTCCATCAGCTCGGGGGAGAGCGTGCCCAGCACCCATAACAGGACGACGGCGTCCATGCGCCGCCAGGACGGCACGGCGGGGAAGGCGTCGTCGGAGAGGACGTGGTCGGCGAGGGCGTATCGCTCGAGGGTGAGGAGGACGAGATCGCGCCAGCGGTTGTAGTGGGAGGAGAGGGGATCCAGAACCACGGGCACGAGGGCACGGATGTTCTGGACGCCCGCCGCCTGCGCGTGGAGATTGGCGATGGTGGCGGCCTCGTAGTCGTGGTCGTCGTCGGCGCCGGCGTCGTCGTGGGAGCCGCCGTCGTCGTGAGCGCCGTCGTTGCGAGGTGGGAAGGCGGCGTCGCGAGCAGCGGTCGCTGCGCGCTCGAGATCGGCAGCGGCCCGGCGTTCCTGCGCGAGGACGTCCTGGGCGGCGCGGACGCGATCTTGAGCGGCGGCGGCGGCCTTGCCAGCCTCAGCGGCGGCGTCGACCAGGGCCTTCTCCCGTGCGGCCGCATCCTCGGCGCGCTGCTGGGTAATAGCGGCAGTGGGAGCGGGCGGAGGATCACCAGGTGCCATGGCGGCGGCGCAGGCAAGTTAGGTTTAGCGGAAGCGAGCTGTCACAGGATCGTGACGATCTCTTGATACCATGATAGCGCGAATAATACCTTGTGTATTCAACTGAGAAAACAGGAGTACAATATATAGACTCTACCGGTAATCTATCTCTACCTGATTTATAGCAACAAGATCAGGGAGAAGATCCGGGAGTCCAGGCGCATAAGGAAAAGCTAGGCATGGAAAGAGTCAACAGCCAGCCACCTAAATATAGAGGGGATCACCTAAACAGGGAAAGAGATAAACTCTAACAAAACCGACAGCAGTAATCACTGAGGTAAAACGCGCAAACCAGGCACGAGGAGTGCCTGTGATGGGACCTACGATTCTTTTGTCTGACAGTATAGATGCTATCAGTATTGCTTGGGACCCAGTGAAACATGAGCTCACCAAGCACATTGGTGTGGATGATTCATCTCACAGGTGCAGGAGGGGGTACTTGCTTTACAGTTTGTGCTATCAGAGTTACAGGTTGCTGACTTCTTCACGAAGCCACAGACTAGGACACAACATTTGTATTTTCTCGCCAAACTCAGTGTGTGTGATCCACCATGAGTTTAAGGGGGGGGGGGTGTTAGAATAGGACTCTATTTGTATTTCCTTATTTGTATATGGCAACCCCCTTTGTACTATCTATATATCTTACCCATTAGGCCCTTTTGAATATACACACAATATTCATAACAGTGTTGTACGATATCCACAACAGTTTATAATATAACATTTGGATCTTTGATATGCTGCAGGTAATCAACATGGAATGGGGAAACTTCAAGTCAGATAAGCTTCCTTGTTCAGAATATGATAGTGCCTTGGATTTTGAAAGTTTGAACCCTGGCGAGCAGGTATTGTTTATTTAACCTGTTTATTCCCAACTATATGTGTGTGTGTGTGTTTGAAAAGAAATTTTGTCTTGTTTTCCTCAGATATACGAAAAGATGATTTCTGGCATGTATCTTGGAGAGATTGTACGAAGAATCTTGCTGAAGTTGGCTCATGATGCATCCTTGTTTGGGGATGTTGTTCCACCAAAATTGGAGCAACTTTTTATACTGAGGTGCACTTTCATGTTCCATCTATTGATCATCTAGCTTTTCTTGTATTTGTAAGAATTTGGAAACAATCAAAACATGGAAAGCTCATGTAACCGATAGAAGCTTTCTTAAGGAAGTTCCCAGTGTGCTATTGCAGGACACCGGATATGTCAGCGATGCATCATGACACCTCACATGATCTCAAACACCTCGGGGCTAAGCTGAAGGATATTCTGGGGGTATGATGATGTTGAAAAGGCGATACTGTTAAATCTAATCTTCAAGAACCACTTATTCCTTACATTATGTTTGAACTTGTATTTAGGTCGCTGACACTTCCCTGGAAGCAAGATACATTACTCTTCACATCTGCGACCTTGTCGCGGAGAGAGGTGCACGCTTGGCTGCTGCTGGTATATATGGCATTCTAAAGAAGCTGGGCAGGGACAGAGTGCCAAGTGACGGTAGTCAAAAGCAAAGGACTGTCATTGCTATGGATGGTGGTCTCTACGAGCATTACAAGAAGTTCAGCGCCTGCCTAGAATCGACGCTTGCAGACCTGCTCGGGGAGGCCGCCTCATTGGTTGTAGTCAAGTTGGCCAACGATGGCTCTGGCATTGGTGCTGCACTTCTTGCAGCCTCACACTCCCAGTATGCTGATGTCGAATATTCCTAGGAGCTCAGGGACCGAGCTCCTAGTGCAGCTTCTTGTTTTCCTCTTCTTTTTTTTTTTTTTTAAAAAATTCTTTCTATTGAGAGTTCCCCTCGTGGGATTCGCATATCTCTTTTTGCTATTCTGCAGACACATAAATGAGTGCCTGTGCAGCGGGATATAGCTAGTATGGCGCCAATGAGTCAAGTTTGGAGTGTATCACATTGAAAAAGCATTTGCAACTGCAGGAAAGTGGAAACGGAGGTTTGTATGATGCCGGTTCCTTTCCTGCCAATTCTTTTGCCCCTTTCCCTGTAAGTTTCTTTTGTGATGCGATGTCGCAAACCAAGCTGAGTCGATCTGCTTGTAATCTTCTGTCATTCAGAATAAAGAGGGGGATATGATCAC

>PeHXK7

CGAAAAAGCAAATAAATACGAGTCGGCGCAGGAGCTAGTACGAAGCCGACGCGGCAGCGGCACTTTCCATACCAACCACCTCCGATCCGATCTCGTAATCCAACCTAATTTCCATCGCTGCCCCTCCGAAGACTTGTATAAATCAACAACCGCACGAACCCGAGACCCTCCTCCCAGAAACCAATCGTACGAGCGCTTCTTCGTTCTCGATCCCTGCGCAGTTGCCAGGAAGCTACGACAGAAACACACATCCATACATACAGCTCAGAGCTCACGAGCTAGCTCGATCGATCGATCTAACACCAACCTTTCTTGCTTGCTCCTGATTGCCGATCGCTTCCCTGTGTGGATCTTACTTGGAGCGAGATCTCTGCATCTGTCCCGGTTCTTGGACCCCAGATTAAGGTTCTTGCTCGGGTTAATTTGGTTGGTCGGGAACCATGGCGGCGGCGGCGGCGGCGGTGGCTGAGCAGGTGGTGGAGGAGCTCCGGGAGGCGTGCGCGACGCCGGCGCCGCGGCTGAGCGACGTGGCGGCGGCGATGGAGGAGGAGATGACGGCGGGGCTGGCGGAGGAGGGCGGCAGCAAAATTAAGATGATCATCTCCTACGTCGACAACCTCCCCAACGGGTAATTAAGTAATTATAAATTTACCTACTCCTCCTCAGCGCTTAGCAGTTAGCCCCTTTTCATTGTTCTGCTGGTAAATTTCTTGGAAGAAAACATTGCAAGAACAGAACAGAAAAAAAAGAGAGTCATGCTTTGGTTTTTGGGTGCCATGAACAGCAATAATAAATCTTTAGGGAATTGCTAGAAATCTGTCGACTGAAATTGAGGCCGTCCGATGCACTCTATGATTGGTGAGAGAGAGGGAGAGAGGGTGGGAAGTGAGATTAGATTAGGGATATAGAGAGGAGTGATATCTAGGGCATTGGATCTCTATCCATTGGCTCAAAAAGTGACTGATTTCCAAGAAAAAAATCAATCAAATGTTGTATAGACGCACCCAAATCCTTAATGCTTGTGATGTTTTGCCTAGTCGAGTACTACTAGCTTGCTAATTCCTTTGCTTAAGTTGGTAATGACCAAGTTTGATACCTCATTTCCGGAGGTGTTTTACTTGTACTATTTGGCTTCATTCAGCTAGCTACCAGCTGAATTTCTCGTGCTGCCAGATGTACTAGTACGTTGCATACTTGCATGTGACATCGTTGCGTCTCGAATTGGATGGTTCCGCGGCTGCTAACTGTTTGTATTTTTTTTTTCTCTCCCGCTTATTTGATTGGCCGAGCTCCGGTCCTTTCCTTGAAAAACAGAAAGTGTTGGAATCGCAAAGTTTTTTCAAATTGACACGAACTAGCTAGAATTTATTACCACCGCTGCACCATTTTCGTAATCTGTTTGCTTACTTCCTTGCGCTACTGAATTTGATCAGGAGTGAAGAGGGCTTGTTCTACGCGCTGGACCTCGGGGGAACCAACTTCCGCGTCCTGCGCGTGCAGCTCGCCGGCAAGGAGAAGCGCGTCGTCCGGCGAGAGGCCAGGGAGGTGTCCATCCCTCCCCACCTCATGTCAGGCAGCGCCTCGGAGCTGTTTGGCTTCATCGCCTCCGCGCTGGCCAAGTTCGTCGCCGACGAAGGCCACAGCGGCGCGTTGGACGGCGGCAGGCAGAGGGAGCTGGGGTTCACCTTCTCCTTCCCCGTGAGGCAGTCGTCCATCGCGTCCGGCACGCTGATCAAGTGGACCAAGGCGTTTTCGATCGATGATGCGGTGAGTATTATTGTCCTTGATTCGCGTGGCCGTACATCCGACAAATTAATCCATGCGTTAATTGTGGACACTTCAATGCTAATGTACAATTTATCTTTTTGTTAGAACTGTTATCGTTCTTTTTCAGAAAGTTTTTTCTGAAATTACAACTTCTCTGGAATATGTCAGGTGGGCGAAGATGTGGTAGCTGAACTGCAGACGGCCATGGAGAAGCAAGGTCTTGACATGCGCGTGTCCGCATTGGTTAGTCCTGTTCTGATCTGATCATATTTGTGTCAGCACTGTTCTCAAACAAACAAAATATGTTTTTTATGATGAGACCCCATTCTGACATTACTTGTTCATGGTTCAGATCAATGACACCGTCGGGACACTGGCTGCGGGCAGCTACTACGACGAAGATGTGGTTGTCGGCGTGATATTAGGTACTGGCTCGAACGCCGCTTATGTCGAAAAGGCAAATGCCATACCAAAGTTGGAAGGCGAGCTACCAAAATCAGGAAATATGGTAAGGTTCAAAGACTTTTCACTTCAAGTAGCTAGTGTATTCTTACTGCACAAAATCATTTGTCTTCCTTTCGCAATCTTCTCACTGTACTTTATGCATTTCAGGTTATCAATACAGAATGGGGCAACTTCAGTTCATCGTGCCTTCCGATAACGGAATATGATGAAGCATTGGATGAGGAGAGCTTAAACCCGGGGGAGCAGGCAAGCTTCTCCAGAAGATATGCAGAGTCCTCAATTCATGATTAAGCTGTAACTTACACTGTATAACTATGCCTTGTTTTTTTCAGATCTTCGAGAAGTTGATTTCAGGGATGTACCTAGGCGAAATCGTGAGGAGGGTGCTTCTTAAAATCTCCTCGCAGTCTTCGATTTTCGGCAATATCAAACGCACCATGCTCAACACTCGCTTCATCCTGAGGACCCCTGATATATCTGCGATGCACCACGACGAAACACCTGATCTGAGGATTGTCGCCGAAAAACTGGCAGAGAACCTGAAGATCAAAGGCACGTCCTTAGAGACGAGGAAGATGGTCGTCGAAATCTGCGATATTGTGACCAGTAGGTCTGCCCGGCTGGCTGCAGCTGGGATTGTTGGGATCCTCAGGAAGATTGGCAGAGGCACCCCCGGCGACGACCGGAAGACGGTCATCGCCATCGACGGCGGCCTCTTTGAGCATTACACCGAATTCCGGCAGTGCCTGGAGAGCACGCTGGGCGGGTTGCTCGGCGAGGAGGCGTCCAAGTTGGTGTCTGTCAAGCTCGCAAACGACGGGTCGGGTCTGGGAGCTGCCCTGATTGCAGCTGCTCACTCTCAGTATCTGAATTGATCAGTGTCCGTAACGAGAGTTTCAGCTTTCCTCAGTGACTTGTGCTCTTGTTATTATGGTGTATTGAACTTCTGTACATTGCAGAGTCCCATCAAACAAATTTTCAAATATAAATGGCCTTGTGTGTGTTGTTGCAATCTCCACGTCCACTGTGTAAAGTTCTAAACTTAATTACAGCGGTATAAAATAGAATCCC

>PeHXK8

AAACTGTTCATCTCCTTTGTATAGACCACCGAAAGAATAAAAAAAGCATCGTGAGTCAGGACCGATGGCCTGAGTCCGGTTTCTTCACACAGTCAATCGCGAAAAGATGAGCCCTTGAAGCCAGATTTCCCGTGCCTCCTCATCTTTTGAGTTGTCCAAATTAAAAGATCCTGACTTACTCTTGTTGGTTTTTAAATTTAAATAGTTTCTTTTTTTTCCGATCGCGCTGTCCAATATCGTACCATTTCTTTCCCCTGAGCTCTTAATCTTCTTGGGTATTTAAATAATTTCCCCCTTCAGTTCTCCAGTCCAAAGATCCTGCCTTTCTCGCCGTTCTCCCCTTCCTATTTCAGTTCTTGAATCCAAAATTGCCCTTGTTTCTTGATTTGCTTGTTTGGAACTGGGATCATGGCCGCAGCTGCGGTCGCAATGGCGGAGCAGGTGGTGGCGGACTTCCGGGAGAAGTGCACAACGCCGGCATCGCTGCTGCGCGATTTGGCGTCGGCGATGGCCGACGAGATGGGCGCGGGCCTGGAGAAGGAGGGTGGGAGCAGGGTCAAAATGCTCCTCTCCTACGTCGATAAGCTCCCCACTGGGTTAGTCCATACGCAGTTCTCGCGTCTTGTTGCTTTGTCTGATCAGACGAAGCTCTGGTTTCTTAATTCTTGTTTTTTGATTGTTTAAATAACAGTAGAAGAAAAACAAAATGTTTCTAGGGGCACCTATTGTGCTTTTCGTTCATGCTTTCTGAAAAAAAATCATTCTCTTTAAACAGGCCATTTCTCGTGCTCATTGTCATCTTATTGAGGAAAAAATAACAATACGCTGTTTACGTTGATTTTATCGATGTAGATGCTGACATCAGAGACTATGGTCATAACTCATATGATATTATAATTAAGAATAATATGATAAGGGCGCCTGTTAATGGAATGACTAGAATAAGATGACCATTCGACTGAACTCATGGATGTGGATGTATGGCCATTACAAAATAGAAATGTTAGCTCCTTATTATGTTGATACTGCTAAAATACGATGCATTCGTGTGGTATTTTATCGGGTTTCAGGTTAGCTGATAGATATATTCAACTATGAAGATTGTAGATTTCAATCGGCAATTTTATGTTTAGCCTGTGGTCACAACTAGAATTATTTGGCTGCTTACTTCCATGCTACTACTAAATTTTAGGAGGGAGGAAGGTTTGTTCTATGGATTGGACCTAGGAGGAACGAACTTCCGCGTTTTGAAGGTGCAGCTGGGCGGCAATAAGAAGCATGTCGTGAACCGTGAGTCCAGAGAAGTCACCATTCCACCCCATTTGATGTTAGGGAGCTCCTCTGTAAGTATACATGTTTAGAATTTAACTAGTATGTTAGATTTAGATTTGATGTTATAGATTATCGACTTTTCAGGAACTGTTTGGTTTCATTGCATCTGAATTAGCAAAGTTTGTTGCTGATGAAGAGAAGTGTGCTAACTCATCAAACCGGAAGCAACGAGAACTAGGATTCACATTTTCTTTCCCAGTGAGGCAGCGTTCTGTTGCATCGGGTACCCTTGTCAAGTGGACAAAAGCATTTTCTATTGATGATGCTGTAAGACTTCCTTGTGCTTAGAAATTGGACATATCTCTGTACATTATGCAACATAAAAATGATAATGCCAATATTACAGCTCTTTGAACTCCCATATTGTCATTTCAGTCTTGCCATAATGGAAAAAATAAATTTACTAAATTGTTTGAATAACTATGTTTTGTCAGATCAACTATACACATCAAAAATCGAATAGCTCGACTGTTTACAGTTGAATCTTCCATCTAATTAACATTTTCTCTGAAATCAGGTAGGTGAAGATGTAGTGGCTGAACTGCAAACGGCTATGGAGAAGCAAGGTCTGGACATGCATGTGGCAGCATTGGTGAGTGTTGACAGGTTTATATATTGCCAGTGCTCCTACATAAAGATAATCTATCTGTAATCTTAGAAGAAGAATCTCACTCCGAGAGATCTTTTAATGAAATAGATTAATGATGCTGTTGGGACGTTGGCTGGAGCCAGATACTACGATGAAGATGTTGTCGCAGGTGTGATATTTGGTACTGGCACAAATGCCGCATATGTTGAGAAGGTAAATGCTATACCAAAATGGGAAGGAGAGCTGCCTAGTTCAGGGGATATGGTAAGGTTTTGAATGCCCTACTTTTCACTTAAAACCTAGTAGTACTCAGAGCATATATGAATTGGATTCTGCTGAACTATGTTATTGTATTTCTAAATAATTTCTTTTAGTGTTTTTCCTGTATTTCAGATCATCAATATGGAATGGGGTAACTTCTATTCATCCCATTTTCCAGTCACTGAATACGATCAAGCATTAGATAAGGAAAGCTTAAATCCCGGAGAGCAGGCAAGTTTCGCCACAAAAGACACCATAGTCTGCCTTTCACACTGTTGGCTCACTTGTGTACTTTTGGCTTCTTTCAGATCTACGAGAAGTTAACATCAGGAATGTATTTAGGTGAAATTGCTAGGAGGGTGCTGCTTGAAATGTCCTTGCAATCTGCAATTTTCGGCGATATTGATCACACTAAGCTCAAAACTCATTTCCTCCTGCGGTATGTTGCTTGCTTAGTTTTTCACAGTTGACGCACTAATGTGACGTATAATTCAGGATTTGAAAGCTCTATTGTATTCTGAAAATAACATTTATGCTCCTATTGATGCTCAACACACTGTTTAGTTAAGTCCTCTGATAAATCTCGGTTTATGTGGTATACAGGACTCCACACATTTCTGCAATGCACCATGACGAAACCCCTGATCTGAAGGATGTGGCTGAAAAACTGGAAGAAAACCTAGAGGTATGCAAGCTTTTTTACCATTGATTGGCAGTCCTGTCCCCTTTATCCATGCGCATCCTTGTGTATGCACTGTAGTGATATGATATGCATGTTTGCAGATTACAGGCACATCCTTAGAGACAAGAAAAATGGTTGTCGAAATCTGTGACATTGTGTCAAGAAGGGCAGCCCGGCTGGCCGCTGCAGGGGTTGCAGGAATCCTCAAGAAGCTTGGGAGAGATGGCCCCATCGGCAAGCAGCGTTCAGTCATTGCCATTGATGGCGGATTGTTCGAACACTACACCAAATTCCGCGAATGCTTGGAAAGCACACTGGATGAGTTGCTGGGAGAGGAGGCATCGAAGTCGGTAGCCGTCAAGCACATGGGTGATGGTTCAGGGATAGGGGCTGCCCTGATTGCAGCTTCTCAATCTCAGTACAAGTATGTTGAGCAACAGTAGGCAAAAAATACTTTGCTGTACGTGCAGGAAAGATAGCTACCGCCTACCAGGTAGCTAAGCTTCTTTCCCTTTTTCAGTTTTTCTGGGGTGAAAGAAAACCCTGCATGTACAGTTATCAATGCTGGAACTGGAAGCAGTCCTGTCACCAATATGAGACTTGGTCAGTTCACATTGCATAGTTCAAATAAAAGGGGAATTGCAGAGGTCTCAGAAACCTCCAATGCTGCCTACAGTTACCTTTTTT

>PeHXK9

ATGGTGGAGGAGATGGCGCGCGGGCTGCGCGCCGACCCCCACGCCTCGCTCAAGATGCTCATAAGCTACGTCGACAACCTCCCCACTGGGTAATGCTTCCTCTGCTTCTGCGATTTTTTCGTCTTTTGGGGCGTGTATGTGTGTGACCCGAGTGGTACGCTTGACGCTTGTTCTTGTGCGTGGGCGCGTGTGTCTTGGGGGTGAATTGCTTGGAAATTTGCGAATTTTGGGGTGGCTCGTTGAGGTGCGGATTCGGTAGAAATGTGGCATTGTTCCAGTGTTGGGGTGATTTTTATTTGATCTTGCTACGGTTCTTATTGATTCTGGAAGAAGGATGGGACCTTTTGTTTTCCCTGTGTGGTTTTTTTTTAACCAGGCCCTGTGTGGATATTTGGTTTTGTGGGCACCTTCGAATTTGAGTACTATCTTTGACTAGACACCTCAATTCCAACTTAGATTATATAGGATTTCCGCATAAAGAAACACGGTGTGGATCAGGACATCAGGAGTAAAATATGTTTTTTTACATGCTTTTCAGTATAGCGTTTGGTGTGCAATTTTTGGTCTGAATGCAGGTTTCTCACTGTTAGTGGTAGGCCATGAGGGTGGACAGAATTATACTATAAACAGTTACGGTTTGAATGTTTGCCGTAGCTGATAGATCAACCCATATCTTCTCTAATTTGTGTCCGCTGTAGAGACACGTGTATCCACATGTCCTCTCTAGTTCTCTTAATAAATGGATCATTTGTATTTGTTGAGGAATTGTCACTGACGAGTCTAGTCCGAGCGATTATACAAACTTAGAATCATAAGAATAATACAAGAACACATGAACAGAATAGCAGATTGAGCACAGAGATATAAGGGGACTTCTGGTTTCTCTTTATTGTTTTTCTGTAAACGGTAGTACAATGGTTGGGGTCCCACATCCTTTATATAAGCTACTGGATGGATGAGAAGGCCATCGTGGGCCAGAACCGCCGGCTTGGGCTCGATTTCTTAACACTTCCCCTTGGGCACTCATCCGTTATATTTATATCTCGTCAAAACTTCATAAAAACCTAGTGGAAAAAATTTGGAGAAAGTGTACGCGAACACGTTCTGTTGTGCTGCATGAATTGCCTTATTAAATACCTTAACCAAGAAACTTCAGAAAAACTCATTAAGGAAAAAAGAGTACAATCCACCCAATTTTATTTAATCTTCAGGATTTATTCTTTCGATACTCGATCTCCGCGATCAAGTTTCAATAATGAGTTCGTGAAAATTCTCCCCCTAAATTTTGCATTTCTCTAAGTCTCATCATTCTAATTCCACGTTTGCATCTTTCAAAACTAGACGCTGAAAGTGACTTAGTAAATAAATCTGTAAAATTTTCACATGACTTAGTATGCAGTACTTTGATTTCGTTCATCTTTTGCAATTCATGAGCATAAAAGAATTTGGGGTTGATATGCTTTGTGAGTTTACTCTTCACATAACCCATCTGCACTTGTGCAACATAAGCTGCATTATCTTCATAGATAATGGTGGTGGTTTGCACGATATTCAATCCATATGACTGCTGAATGTAGTGAATCATCTGCCGAAGCCAAGCACATTCTTGTGAGGTCTCGTATAGTGCTATGATTTCTGAGTGGTTCGTCGAAGTCGATATAAGACTTTGTTTAGACGATTTCTATGAAATTGCAGTTCCACCACATAGGAATACATAGTATATTTGTGACTTAGTTGTATGTGGGTCTGATAGGTACCCGACATCTGCATAGCCAATAATAGTTAGGTCTTAGTTCTTTCTGTAGAACAGACCAAGATCTTTGCTTCCTTGGAGGTATCGGTACATGTCCTTCATTCCCTGCCAATGCCTTTGGTAGGTTCTGCACTATACCTTGCTAATAAGTTCATGGCAAATGCAATGTCCGAATTAGTACAGTTTGCTAAATACATGAGTGCTTCTATTGCACTTAAGTATGGGAATTCTGGTCCCAGAACTTCCTCCCCCTCTTCTCTAGGTCTGTATGGATCTTTGTCTTGCTGCAAGGACCTTCCGACCATTGGTACTTTCGAAGGGTATGTCTTTTCAAAACCAAACCTTTCCAACACCTTCTGAGTGTAGGTTGACTGATGCATTAAAATCCCTTCAGGGGCATGCTCAAGCTGCAAGCCTAAGCAATATTTAGTTTTACCCAAACCTTTCATTTTGAACTCCGACTGCAGGTAAGAGCTTGCTTTTTTTAATACCTTCCAAGGTTCTAATGATATTCAGATCATCTTCATACACTGAGATTATACAAAATCCATCTTGGGATCGTCGTATAAATACACATAAATTATTCTCATTGTTCGTGTAGCCTTTTTTGCATAGAAAGTCGCTTCAAGTGATTATACCACATTCTGCCTGATTTCTCAAGTCCGTACAGTGACTTTATGAGTTTCACACTGTACAGATTTCTAATTGCTCTGTCTTGTTTCTATTAAGTTGTATGCACCAACCAATTCACCCAAAAGCTTAAGCTGATGAGGAAAGGTAGGCAATTCACTTATACTTCAACACTCCCCCTCACGTGTAGGCTCCCTCAGGCCTAAGACGTGGAATAGAAATGGGCTGCAATTATTTTTCTTAATTGCGCCAGCCGGGTCTTGAACTCAAGACCTCTTGGCTCTAATACCATATCCCGAAGTTGACTTGCGGGTGTCAGGACACCCGGCCCAATCAGCGTCTGTATAGGCGGTGAGCGAAGTTGGTGAAGTCCGATGTAGTTTTAACTTTTAAGCCGTGGTCGATGGTGCCCTTGAGGTAGCGAAGGATGCGTTTCAGAAGATTGGCATGAGGCTCGCGCGGGTCGTGCATGTGAAGGCACGCTTGCTGAACGGCGTACGCGATGTCCGGACGAGTGAACGTCAGGTACTAGAGGGCACCGGCGATGCTCCTGTAATCAGTGGCATTAGCCACTGGGTTTCCGGCCGCAGGGAGCTTGGCGCTGGTGTCGACTGGAGTACTACAGGGGTTGCAGTTAGTCATGCCGGCACGAGCAAGGATGTCCTCGATGTACTGGCGCTGCGAGAGGAAGAAGTCGGAGGCGGTGCGTTGTACGTGGACGCCGAGGAAGAAATGCGGGTCGCCGAGGTCCTTCAATGAAAATTCGCGGTGAAGCGAACTAATCACCTGTCGTAGGAGAGTCGGTGACGACGCAGTGAGCACAATGTCGTCGACGTATAGCAGGAGGTAGACGGTGTCATTGCCATGCCGGTAGATGAACAACGAAGGATCCGCCTTGGACGCCGTGAAGCCCAGCGAGGCAAGGTAGGTCGCAAAGCGACTGAACCAAGCCCGTGGAGCTTGCTTGAGGCCGTAGAGGGACCGGTTGAGGCGACAGACGTGCGTGCTGCGTGTCGTGTCGACAAAACCGGATGGTTGTTGACAGTAGACGGTCTCCGTGAGCGTGCCGTTTAGGAAGGCGTTGTTGACGTCGAGTTGATGCACCGGCCAACCGTTAGCCAGTGCGATGGTGAGGACGGTGCGTACGGTAGCTGGTTTGATAACCGGGCTGAACGTCTCACCGAAGTCAACATCGGGGTGTTGGGAGAAGCCCCGGAGTACCCATCGCGCCTTGTAGCGATCCAAGGAGCCATCAGGCTTCAACTTGTGTCGAAAAATCCACTTTCCTGAAACAACATTAGTACCAGGAGGTTTTGGAACAAGTACCCATGTGTTGTTAGTTTGCAAGGCATCAAACTCGCGTTGCATGGCGAGACGCCAATTCTCGTCGTTAAGCGCGGTGCGGTAGGTGCGTGGAAGCGGTGAGGGGACGTCCACGTGGAGGTTTAGCCGGTCCATGGGAAGTCGAAAACCACGCTTACCATGTGTGGTCATCGTGTGATCATTGACCACCGGGGTGATGGCCTGAGCCGCAGGTGGTGGCGGTGCTGTCGCGGCGTCTGACAAAGTCGCAGGCGCCAGCACAGAGGGCTGCTCGGCCTGCTGGGTAGCCGCAGGCGCCGACGTAGGGGGCTGCGCGGCCTGCTGAGGAGCTGTAGGCGCCGGCACAAGAGGCTGCATGGCCTGCTGAGGAGCCGCAGGCGCCGGCACAGGAGGCTGTGCGGCCTGCTGAGGAGCCGCAGGCGCCAGCGCAGTGGAACGCGCGGCCTGCAGGTGAGGCTCGGTTGACGGGGGCACCAATGCATGCTGGAGTGGCGCAGGCACAAGAGATGATGCGGGTGCCCTAGGACGTGCAATGGGAGGTAAAGCGGCAGTCGGTGCATGTGCAGCGTGTGACAGGAAATCAAAGTCCTCAGGGACTGGGGAATTAGGCTGCTCGGAAAAAGGGAATGAAAATTCGTCAAAAGTGACGTGACGGGATATGATGATGCGGTTTGTGGTGAGATCAAGGCAGCGATACCCTTGTGGTTTGCCGGATATCCCAGAAAAACGCAAGCCGCAGAACGTGGTGCGAGCTTATGAGCGGCAGTGGCGGAGAGGTTAGGATAACACCTACAGCCGAAAACTCGGAGATGTGAGTAGTCGGGAGGAGTGCCAAAAAGAGTTTGGTGTGGGATACCGAAGTTGAGTGATTTTGTGGGGTGAAGATTAAGAAGATAAGTAGCGGCGTGAAGTGCCTCGACCCAGTAGGAAGGTGGCATGCTGGCTTGGAACAGAAGGGAACGTAGGACGTCGTTGGTAGTACGGATGATGCGTTTCGCCTTACCGTTTTGTTGAGATGTGTGTGGACATGACATGCGGAGTGCGATGCCATGGGAGAGAAGGTGCAGGCGCATGGCATGATTGTCAAATTCGCCACCATTGTCACATTGGATGCTACGCACAGTGGTAGAAAATTGAGTGCCTATATGAGCAAAGAAATTGCGGAGTGTGTCGTACACATCAGACTTTAGGCGAAGTGGGAACGTCCACAGGTAGTGAGTGAAATCATCTAGGATGACTAGATAATACTTGTAGCTGGAGATGCTAGGGACGGGTGAGGTCCAGAGATCACAGTGTATCAACTCAAAGGCTTTATTAGTGCGAGTAGTAGATGCATAAAACGGGAGACGTACATGGCGGCCTAATTGACACGCATGACACAAAGAGTCTACCGAGGCTTTATTACATGAAATATGAGACGCACTAGCTAGATGCGATAAAGCTTGAAGACCAAGATGACCGAGTCGTCGATGCCACAAGGTGGAGGAAGGTGTGGTGATTGCAAGAGCATGTGAGGTGGGCGGCGGCCAACGGAGAGGATAGAGGTCACCACGGCTATTGCACCTGATGATCACGTTCTTAGTTTGAAGATCCTTCACAGAAAGACCAAAAGGGTCAAACTCAACAGAGACGTGATTATCTAAAGTGAAACGACGAACGGAAATGAGGTTTTTGATTAAGTTAGGCGTAACAAGAATATTGTTTAACTGAAAACAATGGGATGGAGTGTGGAGAGATGCTGTGCCGGTGTGAGTGATTGGAATACGGGAACCATTACCGACAATAGCATATTGAGATGCAGCGGAAGAATTGCGAGAGGTGGAGGAAAGATTACCATGATCCGAGGTCATGTGAGCGGACGCCCCAGAGTCCATATACCAGTCGGAAGATGGCGGCGCCAGCGTCATGGTGTTGAATGCATGCATGAGCTGGTTTTGGTCCCATGAAGCGGATGGCGCGTAGGAGGGGAATGCAGGCGTGCCCGTCCACTGGGCACCCGGTCCTGGGGAAGGTTCATATGTCGGCGCACCAGACCACTGCATGCCTTGGCCCGACATGTTGGGGACGGTGGCGGTGAAAGCGCCAGGAGGACGTGGGCCAAGCAAGCCCGGGTTGTACCGGGGCCCTTGTCCAGGCCACATTTGGATGGACCCGGCCCATGGGTTTTGGAGCGACGGCCAGGGAGCGGAGCCCTGGCTGCTGTTGTTGGAGCCGCCGCCGCCGCCGCCTTTGAAGGAGCCGCCGCCGCCTTTACCGCGACCGTTGTTGCGGCGGCGGTAGTTGTTGGAGTTGTTGCCGCCGCGGTTGGTGGAGCCGCTGTTGGAGGAGGATCCGCCGGTGGAGTTTTTGTTGGACTGACCGGCCGAAGATGCGACGAGAGCCGACGGGTTGGAGGAGCCGCTTGATGCATCTGTCCTGGACGTGAGTTCCTCAAGGAGGAGAGCGGAACGGGTCTCCAAGAACGTCGGCATAGGACGCTGCATGGAGATCAATGTGCGCATGTGCGCGAAGCGAGAGCTCAGTCCGCACAGAGTGTTGAGGACGAGGCTCCTGTCCAGGATTGGCGTGCCCACGGCAGTCAGTTCATCGGCAAGAGACTTCTGCTTGCGGCAGTAGTCCGCCACCGACATGGACCCCTGCATGAGGCTGTGGAACTCCTGCTCGAGGTAGATGGCGCGCGTGTCGGCGTTGGCGCGGTAGAGGCCCTCGATGCGCGACCAAATAGTGTAGGCAGTGGCCGAGGTGTCCATCACCATGGCGAGGAGGTTCGGGGTGACGGCGGCGTACAGCCACGAGCGGACGAGGGCGTCGAGACAAAGCCACTGCGCATCGGGGGAAGAGGGAGACGCGCGAATGTGGTTGTCGAGGGAGAATTTTTCCAGGGCGACCTCGAACAGGCCGCGCCATTGGCCGTAGTTGTCGGCCTGCAGATCGAGGGTGATGGGGACGAGGAGCTTGACGCTCGAGACGCCGACGGCTTGCGTCCAGAGCGCGACCTTGGCGTCGAGTTCGGCCTGGGCAAGAGCTGCTTCGAGCTCAGCCTGTGCCGCGGTGGCCTGGGCGGTGGTGTCGTCGACGGGACCCGTCATGGCTGGAGAAGATGAGGTTAGGGAAGGAGATGCACATGGGGAGATGTGCGGCTTACATGCCTAAACACCTCTCTTTTATTCAGAGAAAATAATTATGTTTCTATTGCCTTCTTCCAATCTTCCTAATTTGATATTTTCTTACATTCAGCGAGTGATGTAGGCTCTGGATCAAGATCGATGATGTGACAATTTTCTTTGCAAAATAAATGTCGACATTGGTTGTTGTTCTATTCATAAATTCTCCTGTGGTCACATAATTTATAGCTATTTCTTTATGGGGCGATTCTTCTAATATTTCTACTTGCTCACCGCTGCTTCTAATTGCGGGGGCAAATCCTTTTAGTTTTTTAGCTCCGATGTGTGCGCACACATTCATGCTGATATCTCCCTCCGTGGGAATGTTCAAATCCGGTATGCCTTTTACTGAAGGTTTCATACTGGTGCCAGGTCTCAACTGGTTCCCCTTCTGGTTCTAGGGAACTTCAATGATCGGTTGTGTCAAGTCCTTCTTATCCTTTTTCACCAGGCATCCCCGGTTACTTCGGATTCGAGGGGCCGGAGTTTTCGTCCTTTCTGAGGCCTTAGGACATAATTAGGGATACCTTCATCGGTATTTCAACCCTCTCCGGTGCATTGCGTACAGACATATGCGACTTGGTCACGCTCTTCAAATCACTAAAGTGGTCAGACAGTTGATTCACTAATGCTTGCAAATCAATTATCTTCAGGACTTCAGTTATCTTCAGGACTTCTCTGTTTGCTTCACAAGTGCGCGGGTCATGTGCATAGGTTTCAATTATCTTCTGGACTTCAATTATTTTCCGACATTTTTCATCAAGGGGTTAATTCCCCCCCTAATGACGGGAAACAATCCTCATCAAATATGCTATCAGCGAAGCGGACTACATGCAAATTTCTAGTCATGGGGTCCAAATACCTGATTATGGATATTGTCTCATAACCAATGTATATTCCAGACTTTTGGAGAGGGCCCATTGCAGTTCGCTGCGATGGTGGTATAGGTACATATACCTTGCAACCAAATCTGCGAAGATGAAAAATTTCCGGTATTGACCATTGCGCTAGTTGCATAGGTGAATGGATTTTATATGCGGTGGTCTAAAATTGATCGACCATGCCTCAACGCATGTATAACTACCTAGTTACGGAGTAGCGTTTGGCAACCCCTAAGTAAGTCACTACACATCTCGAGTACGTGCGACGATCTCAAGTTTAAGGATATAGCATACGCATTGTAAATGAGACCAACTGCAGTGTCTCATGGTGGGTCGATCCAACCCCATGTTCGCTAACATGTGTCCATATTATTGGTTTGATATCTCTATATCTATGATTCGTAAAACATGGTCATCAACCGATACATATGTTAGTCTATTCATATGTGTCCTCATATGTTTCCGATTAGGGATCACTTTAGAATAATCATCGCAAAGATAAATATAGAGTTTCACAATCAAGTCACATACTTGATAATAAATATTAATGGTATTATTTTTGGAACAAGATAACATATTATTCAATAAATTATGAACATAACGAAATATAATTATCTCTATGATTGCCTCTAGGGCATATTTCCAACATTTATGAAATCTACTTATGTTGTTTTCTCTGCTATTATTTTGATGTTTACTGATTTGGAGGTCACTTCTGCAGGGATGAGCATGGATTGTTTTATGCACTGGATCTTGGTGGGACCAACTTCCGTGTTATACGGGTTCAGCTTGGAGGAAGGGAGAAGCGTGTTGTCATGCAACAATACGAAGAGGTGTCCATTCCACCTCATCTGATGGTTGGGGCTTCCACGGTAAGTGCTTTCAATTGCTCTTTTCAAGTCTCCTGCATTAGCAAAATTTTCTTGCATGCTTTACTCACATTCACAAAGCATGAGTACAGGAACTGTTTGATTTCATTGCGGCTGAGTTGGAAAAATTTGTCGAGACTGAAGGAGAAGATTTCCACTTGCCAGTGGGCAGGCAGAGAGAACTGGGTTTCACCTTTTCTTTCCCAGTGCACCAGACATCAATATCATCAGGCACTCTCATTAAGTGGACAAAGGGATTTTCCATCAATGGCACGGTAAAAATCAAATGACCTTAGTTCATCCTTCGCTGACTGAATGATATACTAATATTTCTATTACCTTGATGATCAAGGCTTGATCAGAAGCCCAATTTCTTATCAACAAATGATTCTCCCTATGTTATTTCCCTGATCATCTTAATGTGGCAATTAAACTTACCACATTACATTTTCCTGACAACACTTATTAAGCGGACGAAGGACTAGCTTTCAGTGATATCATTCTGCGCTCTTTTCACTTTTGTGAACTCCCTGCACTGACAAGTGGCCAGCTGCTGTCCTGCATTGTTCTTTCATGATATTAGAACTGTTTTTTACTGGAAAAATAGAATATTAGTATGAAATGTTTTTGATTCGATTTTGAACATGTGTAGATTATAGGCCATTTTTTTTTGGGGGGGGGGGCTTGACCTAATTTAGAGAATGTTTAGAAAGCTTACTCTTGAAGTGATAAAAAGGATCCATGTTTCTTAGGATTTAAGTTATTTCTGTCAATTGTCAAGTATAACTAGCCATAAATCCCGCACAATTGCGTGGCTAGATGCTAGAATGCATGACTTTATATTTTAAAGTAAGTTGTATTTGTTATACCACTATAAAATATTTAGCTGAAAGTACAGTTCTATTTGTTATATAATTGTAATAGCCCTAATAACTCTATTTGATTAATTTGAACCCACCAAAATAAGTGGTGTAGATATTTATCTTTTTCCTCCTATTAATATGGGAATTTCTAGCCTCTCAGAGCAAACGTAGTGGCGACTTTTAACATCGTTTTAATCATATGATAACAATAAATGACCGTAGTGTTGATTTGGATTATTGATTGCTGTGAGCTCAGGTCGGGGAAGATGTTGTGGCTGAATTGAGCAGGGCTATGGAGAGGCAGGGTCTTGATATGAAAGTTACAGCTCTGGTAAGTAATTCATTTGTTCATTAAATTATTTAGCATCTTAAGCATTCACATGCCTGTCTTTTATATCAAAGTCATTGGCATTTCCTCTGCACTCTGATGCTGGTTTTGGATTTTTGAGATGCTATATGTTGATGGAACGCAAAGGTTGGACACATATTGTATCGGAAAATACTTTCAACGTTGTGTAGACTAACAACTAACTTTTTTTTTTGTGTGTGTAGGTTAATGACACTGTGGGCACATTGGCTGGTGGGAGATATGTTGATAATGATGTTGTTGCTGCTGTAATATTGGGCACTGGCACAAATGCAGCATACGTGGAGCACGCAAATGCAATTCCAAAATGGAATGGGCTACTACCTAGATCAGGAAATATGGTTAGTGTCTGACTTCCTTTGCATGCTTGGAATATTAAAGGTCTTTGGGCCTTGTCTAATTGTATATATCCACTGCAACTGTTGTGTTGTATGCTATTCACATCAGTTAATAATATAACATTTGGATCTTTGGTATGCTGCAGGTAATCAACATGGAATGGGGAAACTTCAAGTCAGATAAGCTTCCTCATTCAGAATATGATAGTGCCTTGGATTTTGAAAGTTTGAACCCTGGCGAGCAGGTATTGTTATTTTAACCTGTTAATTCCCAATGATATGTGGGTGTGTGAAAAGAGATTTTGTCTTCTTTTCCTCAGATATACGAAAAGATGATTTCTGGCATGTATCTTGGAGAGATTGTGCGAAGAATTTTGCTGAAGCTGGCTCATGATGCTTCATTGTTTGGGGATGTTATTCCACCAAAATTGGAGCAACTATTTATACTGAGGTGCACTTTCATGTTCCATCTATTGATCATCTAGCTTTTCTTGTATTAGTAGGAGTTTGGAAACAATCAAAATATGGAAAACTCATGTAACCAATAGAAGCTTTCTTAAGGAAGTTCCAATTGTGGTATTGCAGGACACCGGATATGTCAGCCATGCATCATGACACCTCACATGATCTCAAAAATCTGGGGGCTAAGCTGAAGGATATCCTGGGGGTATGGTGATGTTCAAAAGACGATACTGTTAAATCTAATCTTCAAGAACCACTGATTCCTTACGTTATGCTTGAATTTATATTTAGGTCGCTGATACTTCCCTGGAAGCAAGATACATTACTCTTCACATCTGCGACCTTGTCGCAGAGAGAGGTGCACGCTTGGCTGCTGCTGGTATATATGGCATTCTAAAGAAGCTGGGCAGGGATAGAGTGAAAAGTGACGGTAGTCAAAAGCAAAGGACTGTCATTGCTATGGACGGTGGTCTCTACGAGCATTACAAGAAGTTCAGCACCTGCCTAGAATCGACTCTTGCAGACCTGCTCGGGGAGGAGGCTGCCTCTTCGGTTGTTGTCAAGTTGGCTAATGATGGCTCCGGCATTGGTGCTGCACTTCTTGCAGCCTCACACTCCCAGTATGCTGATGTCGATTATTCCTAGGAGCTGGTGGGATCGAGCTCCTAGTGCAGCTTCTTGTTTTCCTCCCTTTTTTTCATTTTCTTTCTGTTGGGAGTTCCCCTCGTGCGATTCGCGTATCTCCTTTTGCTATTCTGCAGACACATAAATGAGTGTGCTTGTGCAGTGGGATATAGCGAGTATGACGCCAATGAGTTTGGAGTATATCACATGGAACCAGCGTTTGCAACTGCAGGAAAGTGGAAACGGGGGTTTGAATGCTGCCGTTCCTTTCCTGTCAATTCTTTTGCCCCTTGCCCTGTAAGTTTCTTTTGTGATGCGATGTCGCAACCAAGCTGAGCAGATCTGCATGTAATCTTCTGCCATTCAGAATAAAGAGAGGGATATAATCACATAATTGAAATATTTACCATTTCTTCTGTTTGATTGATCAAATATGTATGTAATCTTATACACGTTTACATTGTCAAAACTCAAAATGGCTGACAAGGTAAGTAGTGTACAATCAAGGTTATGGAGCAAAATTGGGACGTGATCGTAATTTCCCTGCCTATAGCTGTTGTCACTTGTCCTCACTAAGGGCGCATCCTGGTTCAGTTTGCACCGTGAAAGAAATTTCTGTCTGTATACATGATCCTCGGAGAGGAAAATGAACCTTCTTATCTTCAGCAATGGTTCACTTCAACAACTGAGCTCGTGGACTCCTGCTCCTGCAACAGCAAGGCACCGATGGGGAGTTCCGGCAGCTCGACGGCGACGGCAACGGGAGGCTGTCGGTGAGGGAGCTCCAGCCGGCCGTCGCGGATATCGGCGCCGCCATCGGGCTGCCCGCGCGGGGGTCGTCGGCGCAGGTGGAACACATCTGCTCAGAGAGCAAGAGTGTATTGCATATACTGTTGTTTTCATCACGAAGCCATACAAAAGAGGAAGCAGATTTTATCTAG

>PeHXK10

ATGTTCGCGGGGCTCGCGTCCGAGGGCGCCAGCAAGGTCCGGATGCTGCTCACCTGCGTCGACGCGCTCCCCGATGGGTACGTGCGGGCGCTCTCCTCCTTCGGTAGCTAGCTTCTTGGTTTCTACTTGTTCTTCGTGTGGTTTTGGTGCGCGTCCTGCTGTATCAGAGAGTTCTTTCTAGGTTCTTGCTTCGTCGTCAACTTGCCATCTTAGTTTGCTTAGTGTGAGCGCGCAAAACTGTTGTTCTTTGCTAGGTTCTCGCTTTCTTGGCGTCCAATGGCACCAATTGATGCGCACTTCTTGTTCTTTTCTCTGCATGTGTTAAAAGATCGCGATTCGTGAGCACAAATCTTGTTAGGAACTAATGGATGCGATCGGGGTATTTGATTTCTTCGTTTACAAATGCCATTTTTCTGGTCCGGTCGTCCGAATGCTACGTTGATTCATCTTGCGTTACTCTTCTGGTCATTGCTCTGTCCTCTTGTCTGGGTCACTTACGGAATCGTTCATAGATTTGGATTGTTGCTATTCTTGCGTGAGCATTGGACACTAGACAGATTTGATCGTTTACTTTTGTAAACTGTTAGAAAATCAGAATATTTATCAATGCCATTTGTTGCTGGTCATTTCTCATTGATATAGAACAATGCCATTTAATTGGAATACAAAGTTAACGTTTTGTTGCAATGGTATATTGGTATGAAGTATGAACAATACCGTTGTTGTGTTCCTAATCAGCATTTCTTGAAATCTTTGAACTTTTATGATGCCGTAGTACAGCATTGGGCCAGTGTCGATCATGTTGGTCTTATGCCACCTTACTTAGGAACTGTTTGGCACTCGCATAATCGTAGGGTGTTGTTACAGGTAGCATGTTTTTTAATAAATCCAACAGACTTTTATCTGTTGTCTGCATTGGGTTCTAGGCCATGTAGTATTCCATAATGGCAATTAGGCGTGGAGCATTTTTCAGACGAAGATTGTTGTACTTGAGGGATTCATGCTTCTTGGTTGGAATGACGTTTGATGTTTAGTATTAGTTGGTGTATTGGAGGTAAAGATAGCTGGAATGGTGTGTTCGTTTAAAATATTTCGTACTTATGGGTTTTAAATCTTTGATGGTATCATTCTTTGCACGGTCCGGCTTCTTATTGGTACTGCCTTGCTTCCTTGTTTTGACTTACGGGTGGAAGAAATTAACAAGCTTCTTCTTATCAACATCTGCCTCTCTACGATACAGTTTATGAACTTTAAAGTAGAATATCCAGACTTCATGTGTAGTAAATTCATACTTGGAATGAGTACGTACTGAGATTGAGACGTTTTAAATGAAGACACTCTTGGTTTAAAAAAAAAAAAATTTGAAGACACTCATGTACATGAAACTAGAACCGAGACCCTTCTCCTTTTTGCTTTCATTGTTGATCTTGCATTGATACCATATTCCTTCCAAACCTGTCCAAATCTGTACTATTTGTCCTTCTTTCAAGTACAGCAGAAGGCTATTTTTTATGGCTTTCCAGAATTCCCGACACACTTATTTGTCCACTGTGGGAATTGTATTGAGAGGACCTGCATCAATGAGAATAATCCTTATACCTATTTAAACTATTTCATGTGCATGTTGCATTCTTTTTTGTTTTATAAAATGATGTAATATTCATGCGTATATTTTATTCAACTAAAAATTTCTGACTGACAACTCAACTTGAGGGCTGTAAGTCATTATTTTTACAAGCAATGATCTTTTCGTGCATAATTTGTTCCTCATTTCTTCACATAGTGCGACTGGTGGACAAGCTTTCCTAAAATACGCTATTGTATAAGTCTTACAGTAGTTCATATTGTGCATGTGACCAGGAGTGAGGAAGGCATCTATTATGCCATTGATCTTGGTGGGACGAGCTTTAGAGTCTTGAAACTAGAATTTGGTGCAGGGTCTATGATCATTAATAAGAAAGTTGAACATCATCCTATCCCTGAAGAATTGACTAAGGGTACAAGCCAGGTATGCTTCATCATTTTATTTCCACTTCCCTACAAGTTAGTGTTTCTGTGTGTGAGTTTTACTTTCTTTTCAGGATCTGTTCAATTTCATTGCCTCAGCACTAAAGAATTTTATTCAAAGGGAGGATGGGAAAGATGTGGGGAGGGAACTTGGTTTTACATTTTCTTTCCCTGTCAGACAACTTTCCATATCCTCAGGGTCATTAATTAGGTGGACTAAAGAATTTTCAATTGAAGAGGCTGTAAGTTACAAAATTACACCTTCAATTATTCTGCTTTTCATATGCCTTCAATTTGAGGCAAATCATTGTATTGTAAGAGGTGATGTGCCAGATGTGAAACTTGGTATCATTATGAGCATGTCTGTGCCATATTTCTTATGCAGTGGCAACATACATAGAAAGCTGGCAGTATTCCAGATTTTCAACTATCACAAGTCACAACAATGACAATAGCAGGCCTTGTAATTTGTTGTCTAGAGAATTTTGTGTCTTTGTTCGTTTGATCCATTTACAGTAGTTTATTTCAACAAACTGCTTTGGCAGAATCATTCAAATAACCTGTGGTTTGGCAGGTTTATTCATATGTGATGCAACATCTTATCGATATATAATTGTTTTTCCCTATAGTTACCTTAATACATTTATAAAGCTTAGTTCCATTGTTTATATCTCAATAAATGGTGCATGACACATTGCGTCAATATGTTCCTTGGTTCCTTGTAATTAATGTTGATTTCTTATGTTATTTTCCATTCTGGCTTCTTTGTACTTTTGGAATATCAGGTCGGGAAAGATGTTGCTCAGTGCTTAAATGAAGCCCTTGTTAGGAATGGACTGAATTTGCAGGTCAATGCACTGGTATGTCATTAGCCTATTTATGTTGGGTGAAGTAGGTTCTGTATTCAAAAACTCATTTCCATTATGAAACAAATATTTCAGTATTTGACTGATTACTTGATTTGAATTGTTACTATGATCCTTTGATTCCATCAATCATTTCTTGTTTATACATTGAGTGTATTAATTAGCCTTCTTAACCAATAATCCACTATATGTGAACACCATTATTTGAAATAGCACGCTACAGCGGACAGGGGGTTGTGATCATTATGTCGTGGCTGCTGTGTAGCATCTCTGTTAATGTTGCAGCGCCGTAGCAGGCTGCTACAGGCTATAGCTTGTAGCATGTAGGGGCACTTCAATACAAAGCCACTTATTGCAAATTCTACCCAACTTTGACTTTGAATTCATACTCCCAGGCTCAACAAGTAGTCTTCATACAAGGTATATTAAAAGAGAAGGCAAACAAGTTAAAAGGTTGGATTCTCCGCATAGGAAAAAAAGCTGAATGTCTAAGATATTTGCATCTTTGAATGAAAGTCATATTGGCCTGTATATTTTGGATGCTTTACATTAAGACTGATCAACTAAATTCATAATTTTGCCAATATAGCCGAGCATGTCCCATTTACCACATCACTGCTATAAGCTATCCACTAAAAAAAGTTGACACTATATTTGGCCATCCCTTATTGCCTAGTTCATCTATCATGTACTGTTGGCCCTGAACTTTTATGACCGTGACTTGCTTTCAAAGTTCGTGTGTCCACATATATATTATGTAATAATGGAAAGAGATGAAACTAAAACATTCGTTCAGTTTTGAATTATTGCGGCATAGCATGATGCTGGCATAGCATAAGTGCACCACCAACATGGCATTAACGATTTATGGAATTTACATGGTTCCTCATGTATTGAGGAAGTGTCGGTATTTGATATTTGTTGAACAAGTCGCATGCTACTTAGTTGCAGTATTGGTGCAAGCTAGATGCTCTGCAATCCGTCCATCCTGTCAAGGTTTATAAATGCCTTCCATCTGTAGCATGCGATAAGTTTCATAAAAATGGCGAACTTTGCCCAAGGGGGATTACACGTAAACTTAGGTGTCCTTAAGTTTCTAAGGATGCTACAAATCCACAGTGTATTGCTCAGAAAACAAAATGTGCACCTTTATTTCCGATATTCTACATTCTGAATCCTGCTGGGCTTAGATAAAAGTATGGTGTCAAATACTTCCTGACTATTAGTGTATAATTTACAGAAGTCTTGTGTCAGTTAATCCTTTAGAATCTTAGTGTTATGTTTCCCTAAATGTGTGCTAACGGTCAGTGTAGTATCTGTTGATTTCCTGTTTTTTGACTGAGATATCAAATCAATTGCAAGTATCTCTAGCTAGAATTGTAGACAGTCACCTGGTAATGCATAATTAAATATATAATTTTCACCTTAGACTTGTATGTTCTCTTCCAAATATAACTGACTAGGGAACTAGGAAGTACTATGCTCCATATCCATTTTGGGAATTTACAAGGGAGAAATAAAGGATGCATATATGTAGCTGAACATATCCATGTAAGTTCAGTTTTGAATTTTTGTGGGTACCCGTAAACAACTTGGTCTATTTTGGCTGGAATGTGGTTCATCAGTTCATGAGGAGGGAGGAGTGGGGATTACAAACTAAGAAGAAATGAACCAGCTTGATTTGGTGTATCGGTTATTTATTTAGAGTATTGTATTGGTGTCTATTATTGCTACATTACTCCACTGCATCATCCGCCATTTTTATGTACTTATGCTCAGCAGAATTGTAGAATTTCATGATTGAATGTTATATGAAAGAACCATGTGCAAATAGGGCTTAGTTTCAGCTTTTGTTAAGCTCGCACATGTTCTTCTGTTCTGATGAATTAGTTGAGAATGATGTGTGCTCCTCGATCAAGTCCAGCAAATTTTCTTTTCAAGTCTGCTGCATGTAATTTATTCACTAGTAGATTAGAACATGTGCATCTTTTGGTTGATTATTTCATGTGCTATTCGAAATAGGTGAACAATACTGTGGGGACATTAGCTCTGGGGCATTATTATGATGAGGATACAGTGGCTGCAGTGATTATTGGAGCTGGCACCAATGCTTGCTATATTGAACGCAATGAGGCAATTACAAAAGGTTGTGGTGTTCTTACCAACTCTGGACTAATGGTATGTTACTTCTAAATTTTACTGTATATGATCAAAATTTCAATGTGGAAATAGAAAGTCTGATGATTGTATCCTATGTTATAATGCTCCATTCATCAGGTTATAAATGTGGAATGGGGGAGTTTCCGGCCTCCGCAAATACCATTAACTCCTTATGACATATGTTTCAATGATGAGAAACAAGATTACTATGACCAGGTAATGCCACAGTGAAAACTTTCCCCTATGGAATATCAAGCTGGAATTAATGTCATTTAATTGATGTAGGGTTTTGAGAAAATGATCTCCGGTGTGTATCTTGGGGAAATTGCAAGATTGGTGTTCCAAAAAATGGCTCAAGAGTCAGATGTATTTGGTATTGCTGCTGATGGTTTATCCACCCCTTTCATCTTAAGGTACTTTCTTACTTGAAATTTGTTTCTTTTGAATTACTTTATTCCTTAGCAAATGAATACCTGTGATAATGCTAAAAATGCAACATGGTCACTGTTCAGATGAATTAAGTGGAATTGGAGCAAGAGATCATTACAGTTAGGAACATTTCTTTGTTTCTTGCGATGCATCCCTGTTAGCTGCATATGCTTTTTTCTTTACTGATCTGCAAATAAACAACATTAGTTGATTTGTAGACATTGGATGATTATGTATGAAATAATGTTTTCATATTCAAATTTCAAGACTCAATGATTTCATAAGATTCTTACTGAGGTCGTTATCTTTAATTTGACATGTCCAATAACTGGTCTAAGCTTCGAAACATTGCATGACAGCTGTGTTTTTGGTGGAACTTCGTGGTCAAGTTTTGTACATTTCAGATGTATGCTTTAGGAAACTCTGTTGTCCAATTGCCTCAGCAGAAAAATACCAGTCATCATCTTGCATGCAGTGATCAAAATTTTAATATGTCATGTCTTTTCATCCAGTACACCATGTCTAGCTGCTATTCGTGAGGATGATTCCCCAGATTTGAGAGAAGTCGGAAGGATACTGGAAGAACATCTGAAGGTTAGTTCTCACAGCCCACCTAACAATCTATACTTTTTTGTTCAGTAGCCATTTTCTTCAGACCAGCATCTCATCTTTCCATTTTCTCATCTTTCCATTATTTCCCATACAGATACCAGATGTTCATCTGAAGACTCGGAGGCTTGTCCAGAGAGTCTGTGACATTGTAACCCGAAGAGCGGCCCGTCTAGCGGCAGCTGGAATTGTTGCAATACTGCAAAAAATCGGTCGTGATGGAACCCTTTGTGGTACCAACAAAGTTCGAAGAATAACAGGCGTGCCGAAGAGATCGGTCATTGCAATCGAGGGTGGCCTGTACCAAGGCTATTCAGTCTTCAGAGAGTATCTGAATGAAGCCGTAAGCGAGATCCTAGGGGAGGAGATTGCGGCCACTGTTAGTCTTAGAGTGATGGAGGAGGGGTCCGGGATTGGGGCCGCCCTCCTTGCAGCTGCATATTCGTCAAATATGCAAAAGTAA
