# Supplementary material for: Genome-Wide Identification and Characterization of Hexokinase Genes in Moso Bamboo (Phyllostachys edulis)
Source: Front Plant Sci. 2020 May 19;11:600. doi: 10.3389/fpls.2020.00600 (PMC7248402; doi:10.3389/fpls.2020.00600)
Supplement: FILE S2 — CDS of PeHXK genes. [file Data_Sheet_2.docx]

CDS of PeHXK genes

>PeHXK1

ATGGAGAAGCAAGGTCTGGACATGCATGTGGCTGCATTGATTAATGATGCTGTTGGGACGTTGGCTGGAGCCAGATACTATGATGAAGATGTTGTCGCAGGCGTGATATTTAGTACTGGCACAAATGCCGCATATGTTGAGAAGGCAAATGCTATACCGAAATGGGAAGGAGAGTTGCCCAATTCAGGGGATATGGTCATCAATATGGAATGGGGTAACTTCTGCTCATCCCATCTTCCAGTCACTGAATACGATCAAGCATTAGATAAGGAAATCCCGGAGAGCAGGCGAATCTACGAGAAGTTAATATCGGGAATTTATTTAGGTGAAATAGTCAGGAGGGTGCTGCTTAAAATGTCCCCGCAATCTGCAATTTTCGGCGATATTGATCACACTAAGCTCAAAACTCATTTCCTTCTGCGATTACAGGCACAGACAAGAAAATTGGTTGTGGAAATCTGTGACATTGTAGCAAGAAGGGCAGCGCGGCTGGCCGCTGCAGGAATCCTCACGAAGCTTGGGAGAGATTGCTCCGTCGACAAGCAGCGGTCAGTCATCGCCATCGATGGCGGACTGTTCGAACACTACACCAAATTCCGCGAATGCTTGGAAAGCACACTGGATGAGCTGCTGGGAGAGGAGGCGTCGAAGTCAGTAGCCGTCGAGCACGCAGACGACGGTTCAGGAGTTGGGGCAGCTCTGATTGCTGCTGCTCAATCTTAA

>PeHXK2

ATGAGGAAGGCAGCGGCGGCGGTTATCGCGACAGTGGAGGCGGTCGGCGTGGCGCTAGTGGTACGGCGGCAGCTGCGGGAGGCGAAGAGGTGGGCGCGCACCGCGGCGGTGCTGCGGGAGCTGGAGGAGCGGTGCGCGGCACGGCCAGCGCGGCTGCGGCAGGTGGCGGATGTGATGGCCGTCGAGATGCATGCGGGGCTCACGTCAGAGGGCAGGAGTAAACTCAAGATGATCATCGGCTACGTCGACTCCCTCCCGTCCGGTGCCTACATTTGGAGAAATGGGCAAGATCTTGTTGATAAATTTGACCGTTTTAAGGAACTATTTGATTTTATTGCTGCTGCTTTGGCAAAATTTGTTGCCTCGGAGGGTGAAGACTTTTATCTTCCTGGGGGGAGGCAGAGAGAACTCGGTTTTACGTTCTCTTTCCCAGTAAAGCAATCTTCAATTGCATCAGGCACTCTTATCAAGTGGACAAAGGGTTTTGCAATTGATGAAACGGTCGGTGAGGATGTGGTGGCTGAATTAGGCAGGGCTCTAGAACGTCAAGGGATCGATATGAAAGTCACAGCATTGGTAAATGATACTATAGAGGCATTGGCTGGTGGGAGATACGATGATAATGATGTCGTTGCTGCTGTTATACTGGGTACAGGTACTAATGCGGCATATGTGGATCGTGCTAATGCAATTCCTCAATGGCATGGCCTCCTACCCAAGTCAGGATATATGGTAATAAATATGGAATGGGGGAACTTCAGGTCATCCCATCTTCCTTTGACTGAATTTGGTCCAGCATTAGATGCTGAAAGTCTGAACCCTGGTGAACAGGTATCATACAAAAGAGCCTCAATATTAGTAGGGATATCCTCATCACGAGTCCTGTTAAAGATGAATCAAGAAGCTTCTGTTTTTGGTGATGTTGTACCACCAAAACTCAAGATTCCGTTTATTCTTAG

>PeHXK3a

ATGGGGAGGGTGGGGTTCGGGGTGGCGGTGGGGTGCGCGGCGGTCACGTGCGCGATCGCCGCGGCGCTGGTGGCGCGCCGGGCGTCGGCGCGGGCGCGGTGGCGGCGGGCGGTCGCGCTGCTGCGCGAGTTCGAGGAGGGCTGCGCCACGCCGCCCGCGCGCCTGCGGCAGGTCGTCGACGCCATGGTCGTCGAGATGCACGCCGGCCTCGCGTCCGACGGCGGGAGCAAGCTCAAGATGCTGCTCACCTTTGTCGACGCGCTCCCCAACGGAAATGAAGAAGGTATTTATTATGCCATTGATCTTGGAGGAACAAACTTGAGAGTCTTGAGAGTAGAAGTTGGTGCGGGGTCTGTAATCGTCAATCGGAAGGTTGAACATCAACCCATCCCTGAGGAATTGACCAATGGTACAACTGAGGATTTATTCAACTTTGTTGCGTTGGCACAAAAGAATTTTGTTGAAAGAGAAGATGGAAATGATGAAAAAAAGGCACTTGGTTTTACATTTTCTTTCCCTGTTAGACAAAATTCAGTGTCTTCAGGGTCATTAATTAGGTGGACTAAAAGATTTTCAATTGAAGACACGGTTGGGAAAGATGTTGCTCAGTGCTTAAATGAAGCGCTTGCTAGGTGTGGATTAAATATGCGAGTCACTGCACTGGTGAATGATACTGTGGGGACATTAGCTCTAGGGCATTATTATGATGAGGATACAGTGGCTGCTGTGATCATTGGCGCTGGCACCAATGCTTGCTATATTGAACGCACTGATGCAATTATTAAGTGTCAGGGTCTTCTTACGAACTCTGGAGGCATGGTAGTAAACATGGAATGGGGGAATTTCTGGTCATCACATTTGCCAAGAACTCCTTATGACATCTCTTTGGACGATGAGACACAAAATCGCAATGATCAGGGTTTTGAGAAAATGATCTCAGGGATGTATCTTGGGGAAATTGCAAGACTGGTTCTCCATAGAATGGCTCAAGAATCAGATGTTTTTGGTGATGCCGCTGATGGTCTATCAACTCCTTTCATTTTGAGCACACCATTTCTGGCTGCAATTCGCGAGGACGATTCACCAGATCTGAGCGAAGTCAGGATGATACTGCGAGAACATCTGAAGATCCCCGACGCCCCTCTGAAAACTCGAAGGCTTGTCGTGAAAGTTTGCGACATCGTCACCCGCAGAGCTGCCCGTCTAGCCGCAGCTGGCATAGCGGGGATACTGAAAAAGCTCGGGCGGGACGGGAGCGGCGCGGCCTCGAGCGGGAGAACGAGAGGGCAGCCGAAGAGGACGGTGGTGGCGATCGAGGGCGGGCTGTACCAGGGGTACCCAGTGTTCAGGGAGTACCTGGACGAAGCCCTGGTGGAGATCCTGGGGGAGGAGGTGGCGCGGAACGTGACGCTGAGGGTGACGGAGGATGGGTCGGGGATCGGAGCTGCTCTCATCGCCGCCGTACATTCGTCGAATAGACAGCAACAAGGAGGTTCCATATAG

>PeHXK3b

ATGGTCGTTGAGATGCACGCCGGCCTCGCGTCCGACGGCGGGAGCAAGCTCAAGATGCTGCTCACCTTCGTCGACGCGCTCCCCAACGGGAACGAAGAAGGTATATATTATGCCATTGATCTTGGAGGAACAAGCATTAGAGTCTTGAGAGTAGAAGTTGGTGCGGGGTCGGTAATCATCAATCGGAAGGTTGAACATCAACCCATACCTGAGGAATTGACCAAGGGTACAACTGAGAGTTTATTCAACTTTGTTGCCTTGGCACTGAAGAATTTTGTTGAAAAAGAAGATGGAAAAGATGAAAAAAGGGCACTTGGTTTTACATTTTCTTTCCCTGTTAGACAAAATTCAGTGTCTTCAGGGTCATTAATTAGGTGGACTAAAGGATTTTCAATTGAAGACACAGTCGGGAAAGATGTTGCTCAGTGCTTAAATGAAGCACTTGCTAGGTGTGGATTAAATGTGCGAGTCACTGCACTGGTGAATGATACTGTGGGGACATTAGCTCTAGGGCATTATTATGATGAGGATACAGTGGCTGCTGTGATCATAGGAGCTGGCACGAATGCTTGCTATATTGAACGCACTGATGCAATTATTAAGTGTCAGGGTCTTCTTACAAACTCTGGAGGCATGGTAGTAAACATGGAATGGGGGAATTTCTGGTCATCACATTTACCAAGAACTCCTTATGACATCTCTTTGGATGACAAAACACAAAATCGCAATGATCAGGGCTTTGAGAAAATGATCTCAGGGATGTATCTTGGGGAAATTGCGAGACTGGTGCTCCATAGAATGGCTCACGAATCAGATGTTTTTGGTGATGCTGCTGATATTCTATCAACCCCTTTCATTTTGAGCACACCACGTCTGGCTGCAATTTGCGAGGACGATTCACCAGATCTAAGCGAAGTCAGAAGGATACTGCAAGAACATCTAAAGATCCCCGACGCCCCTCTGAAAACTCGAAGGCTTGTCGTGAAAGTTTGCGACATCGTCACCCGCAGAGCTGCTCGTCTAGCTGCAGCTGGCATCGTCGGGATACTGAAAAAGCTCGGGCGGGACGGCAGTGGTGCGGCCTCGAGCGGGAGGACGAGAGGGCAGCCGAAGAGGACGGTGGTGGCGATCGAGGGCGGGCTGTACCAGGGGTACCCAGTGTTCAGGGAGTACCTGGACGAAGCCCTGGAGGAGATCCTGGGGGAGGAGGTGGCGCGGAACGTGACGCTGAGGGTGACGGAGGATGGGTCGGGGGTCGGAGCTGCGCTCCTCGCCGCCGTACATTCGTTGAATAGACAGCAACAAGGAGGTTCCATATAG

>PeHXK4

ATGTCCGCCGCCGTATGCTCGCCGATCCCGGCCGCCACCGTCGCGCAGCACCGGCGGAGGTGCGGCGCCGCTGTCCGGTGCTCCGCGGTGGCCGCGCCCATCCTGAACGACCTGAGGCTGCAGTGCGCGACGCCGCTCCCTGTGCTGCGGTGCGTGGCGGACGCCATGGCTGCCGACATGCGCGCCGGGCTCGCCGCGGACGGCGCCGGCGAGCTCAAGATGATCCCCAGCTACGTCTACTCGCTCCCCACGGGGGATGAAACAGGGCTGTTCTATGCTCTGGACCTTGGAGGCACAAACTTTCGGGTGCTGAGGGTACAATTGGGAGGAAAAGATAAGCGTGTTGTCGACACCGAGTTCGAGCAGGTCTCAATCCCAAAAGAAATCATGCATGGTACAACAGAGGAGTTGTTTGATTTTATCGCGTCTGGCCTGTCGAAATTTGTAGCAAAGGAGAGTGATAAGGTTTGTCTTCCGCAAGGATGGAAGAGGGAGATAGGCTTTACATTCTCCTTTCCGGTGAAGCAGACTTCTATTGATTCTGGCATTTTGATCAAGTGGACAAAAGGTTTTGCTGTCTGTGGGACTGCTGGGAAAGATGTGGTTGCTTGTTTAAATGCTGCAATGGAGAGACGGGGGCTTGACATGCGTGTATCTGCCTTGGTAAATGATACTGTCGGAACCTTAGCTGGAGCACGTTATTGGGATGATGACGTGATGGTTGCGGTGATTTTGGGTACTGGCACAAATGCATGCTACATTGAGCGAACTGATGCTATCCCAAAGCTGCAAAACGTTATGCCTGGAGCAGGAAACACGATTATCAACACCGAGTGGGGAGCTTTCTCAGAAGGTCTTCCATTGACTGAATTTGACAGAGACATGGACGATGAGAGCATCAATCCTGGTGAGCAGATATTCGAGAAGACAATTTCTGGGATGTACCTGGGTGAAATTGTTCGGAGAGTGCTGGTCAAGATGGCTAAAGTATCTGATCTGTTTGGTAATTCTTTCCCTGACAGGCTTGCCATGCCATTTGTTCTAAGGACACCACATCTGTGTGCTATGCAGCAAGACAGCTCCGATGATCTTGGGGAGGTCAAGTTAATCTTGAATGACATCATTTGTGTGAAGCAATCTTCTATGGAGGCGAGGAGGATCATTGTAGAAGCCTGTGACTGTATTGTAAAGAGAGGCGGCAGGCTGGCTGGGGCTGGCATTGCAGGAATTCTTCAGAAGATGGAGAATGATTCCAAAGGACTGATCTTTGGACAAAGAACGGTGGTTGCGATGGATGGCGGCCTTTATGAGAACTACCCGCAGTACGGGGAGTACATGAAGGAGGCTGTGGTAGAGCTGCTCGGCCCTGAGGACTCAAAGCACATCGTCGTTGAGCACACCAAAGACGGCTCAGGGATTGGTGCGGCACTGTTGGCCGCTGCCAACTCGAAATATGCAGCTCAGTTGTCGACGTGA

>PeHXK5a

ATGGGGAAGGCGGCGGCGGTGGGGACGGCGGTGGTGGTGTGCGCGGCGGTGGGGGTGGCGGTGGTGCTGGCGCGGCGGCGGCGGCAGCGGAAGGCGGAGCTGGTGGATGCCGCGGAGGCGGATAGGAAGAGGAGGGTGGCGGCGGTGATAGAGGAGGTGGAGAGCAGGTTGGCGACGCCGACGGCGCTGCTGCGGAGCATCTCGGACGCCATGGTGTCCGAGATGGAGCGCGGGCTTTGCGGGGACATCCACGCCACGCTCAAGATGCTCATCACCTACGTCGACAACCTCCCCACCGGAGACGAACATGGGTTGTTCTATGCACTGGATCTTGGAGGGACCAACTTCCGTGTTCTGCGAGTCCAACTTGGAGGAAGGGAGAAACGTGTCGTCAAGCAACAGTATGAAGAGGTCTCCATTCCACCACATCTGATGGTTGGGACTTCCATGGAACTATTCGATTTCATTGCTGCTGCATTGGCAAAATTTGTGGATACTGAAGGTGACGATTTCCACCTCCCAGAGGGGAGACAGAGAGAGCTGGGCTTCACTTTTTCCTTCCCAGTGAACCAAACATCAATATCTTCAGGAACACTCATCAAGTGGACAAAGGGCTTTTCCATCAACGGCACGGTTGGCGAGGATGTTGTCTCTGAGTTAAGCAACGCCATGGAGAGGCAGGGGCTGGATATGAAAGTTACAGCATTGGTTAATGACACAGTCGGCACATTGGCTGGTGGGAGATATATGGACAATGATGTAGTTGCTGCCGTAATATTGGGCACTGGTACAAATGCAGCATATGTTGAGTATGCTAATGCTATTCCTAAATGGAATGGTCTACTGCCTAGATCCGGAAATATGGTAATCAACACGGAATGGGGGAGCTTTAAATCAGACAAGCTTCCTCTTTCAGAATTCGATAAAGCCATGGATTTTGAAAGTTTGAATCCTGGAGAGCAGATATATGAAAAGATGATTTCTGGAATGTATCTTGGAGAGATTGTGCGAAGAATCTTACTGAAACTGGCTCACGATGCTTCTTTGTTTGGGGATGTTGTTCCTACTAAGCTGGAGCAGCCATTTGTACTGAGGACGCCAGATATGTCAGCCATGCATCATGACTCGTCACATGACCTTAAAATTCTGGGAGCTAAGCTAAAGGATGTCGTGGGGGTCCCAAATACTTCCCTGGAAGTAAGATACATTACTCGTCACATCTGCGACATTGTTGCAGAGCGTGGTGCACGCTTGGCTGCTGCTGGTATATATGGCATCTTGAAGAAGCTAGGCCGGGACAAAGTGCCAAGTGATGGCAGTAAAATGCCGAGGACTGTCATTGCCTTGGATGGTGGGCTCTATGAACATTACAAGAAGTTCGGCAGTTGCTTAGAAGCAACTCTCACAGACCTCCTCGGTGAGGATGCCTCGTCTTCGGTAGTTGCCAAGCTGGCCAACGATGGCTCTGGCATTGGAGCTGCTCTCCTTGCAGCCTCGCACTCTCAGTATGCCGAGGTCGACTAG

>PeHXK5b

ATGGGGAAGGCGGCGGCGGTGGGGACGGCGGTGGTGGTGTGCGCGGCTGTGGGGGTGGGGGTGGTGCTGGCGCGGCGGCGGTGGCGGAGGTTGGCGGAGCTGGTGGAGGCCGCGGAGGCGGATAGGAAGAGGAGGGTGGCAGCGGTGATAGAGGAGGTGGAGCAGAGGCTGGCGACGCCGACGGCGCTGCTGCGGAGCATCTCGGACGCCATGGTGTCCGAGATGGAGCGCGGGCTGCGCGGTGACATCCACGCCACGCTCAAGATGCTCATCACCTACGTCGACAACCTCCCCACCGGAGATGAACATGGATTGTTTTATGCACTAGATCTTGGAGGGACCAACTTCCGTGTTCTGCGAGTCCAACTTGGAGGAAGAGAGAAGCGTGTTGTCAAGCAACAGTACGAGGAAGTCTCCATTCCACCACATCTGATGGTAGGGACTTCCATGGAACTATTTGATTTCATTGCTGCTGCATTGGCAAAATTTGTGGATACTGAAGGTGATGATTTCCACCTTCCAGAGGGGAGACAGAGAGAGCTGGGTTTCACCTTTTCCTTCCCAGTGAACCAAACATCAATATCATCGGGAACACTCATCAAGTGGACAAAGGGCTTTTCCATCAACGGCGCGGTGGGCGAGGATGTTGTCTCCGAGTTGAGCAAGGCCGTGGAGAGGCAGGGGCTGGATATGAAAGTTACAGCATTGGTTAATGACACAGTCGGCACATTGGCTGGTGGGAGATATATGGATAACGATGTAGTTGCTGCCGTAATATTGGGCACTGGTACAAATGCAGCATATGTTGAGCATGCCAATGCTATTCCTAAATGGAATGGTCTACTGCCTAGATCTGGAAATATGGTAATCAACACGGAATGGGGGAGCTTTAAATCAGACAAACTTCCTCTTTCAGAATTTGATAAAGCCATGGATTTTGAAAGTTTGAATCCTGGAGAGCAGATATATGAAAAGATGATTTCTGGAATGTATCTGGGAGAGATTGTGCGAAGAATCTTACTGAAACTGGCTCATAATGCTTCTTTGTTTGGGGATGTCGTTCCTCCTAAGCTGGAGCAGCCATTTGTACTGAGGACGCCAGATATGTCAGCCATGCATCATGACTCGTCACATGACCTTAAAATTCTGGGAGCTAAGCTGAAGGATATCGTGGGGGTCCCAGATACTTCCTTGGAAGTAAGATACATTACTCGTCACATCTGCGACATTGTTGCAGAGCGCGGTGCACGCTTGGCTGCTGCTGGTATATATGGCATCCTGAAGAAGCTAGGCCGGGACAAAGTGCCAAGTGATGGCAGTAAAATGCCGAGGACAGTCATTGCCTTGGATGGTGGACTCTATGAACATTACAAGAAGTTCAGCAGTTGCTTAGAAGCAACTCTTACGGACCTCCTGGGGGAGGATCCCTCGTGTTCGGTGGTTGCCAAGCTGGCCAACGATGGCTCTGGCATTGGAGCTGCTCTCCTTGCCGCCTCGCACTCACAGTATGCCGAGGTCCACTAG

>PeHXK6

ATGGTGGAGGAGATGGCGCGCGGACTGCGAGCCGACCCCCACGCCCCGCTCAAGATGCTCATAAGCTACGTCGACAACCTCCCCACTGGGGATGAGCATGGATTGTTTTATGCACTGGATCTTGGCGGGACCAACTTCCGTGTTATACGGGTTCAGCTTGGTGGAAGGGAGAAGCGTGTTATCATGCAACAATATGATGAAGTGTCCATTCCACCTCATCTGATGGTTGGGACTTCCACAGAATTGTTTGATTTCATTGCGGCTGAGTTGGAAAAATTTGTCGAGACTGAAGGAGAAGATTTCCACTTGCCAGAGGGCAGGCAGAGAGAACTGGGTTTCACCTTTTCTTTCCCAGTGCACCAAACATCAATATCGTCAGGCACTCTCATTAAGTGGACAAAGGGATTTTCCATCAATGGCACGGTCGGGGAAGATGTTGTGGCTGAATTGAGCAGGGCTGTGGAGAGGCAGGGTCTTGATATGAAAGTTACAGCTTTGGTTAATGACACTGTAGGTACATTGGCTGGTGGGAGATATGTTGATAATGATGTCGTTGCTGCTGTAATATTGGGCACTGGCACAAATGCAGCATACGTGGAGCATGCAAATGCAATTCCAAAATGGAATGGGCTACTACCTAGATCAGGAAATATGGTAATCAACATGGAATGGGGAAACTTCAAGTCAGATAAGCTTCCTTGTTCAGAATATGATAGTGCCTTGGATTTTGAAAGTTTGAACCCTGGCGAGCAGATATACGAAAAGATGATTTCTGGCATGTATCTTGGAGAGATTGTACGAAGAATCTTGCTGAAGTTGGCTCATGATGCATCCTTGTTTGGGGATGTTGTTCCACCAAAATTGGAGCAACTTTTTATACTGAGGACACCGGATATGTCAGCGATGCATCATGACACCTCACATGATCTCAAACACCTCGGGGCTAAGCTGAAGGATATTCTGGGGGTCGCTGACACTTCCCTGGAAGCAAGATACATTACTCTTCACATCTGCGACCTTGTCGCGGAGAGAGGTGCACGCTTGGCTGCTGCTGGTATATATGGCATTCTAAAGAAGCTGGGCAGGGACAGAGTGCCAAGTGACGGTAGTCAAAAGCAAAGGACTGTCATTGCTATGGATGGTGGTCTCTACGAGCATTACAAGAAGTTCAGCGCCTGCCTAGAATCGACGCTTGCAGACCTGCTCGGGGAGGCCGCCTCATTGGTTGTAGTCAAGTTGGCCAACGATGGCTCTGGCATTGGTGCTGCACTTCTTGCAGCCTCACACTCCCAGTATGCTGATGTCGAATATTCCTAG

>PeHXK7

ATGGCGGCGGCGGCGGCGGCGGTGGCTGAGCAGGTGGTGGAGGAGCTCCGGGAGGCGTGCGCGACGCCGGCGCCGCGGCTGAGCGACGTGGCGGCGGCGATGGAGGAGGAGATGACGGCGGGGCTGGCGGAGGAGGGCGGCAGCAAAATTAAGATGATCATCTCCTACGTCGACAACCTCCCCAACGGGAGTGAAGAGGGCTTGTTCTACGCGCTGGACCTCGGGGGAACCAACTTCCGCGTCCTGCGCGTGCAGCTCGCCGGCAAGGAGAAGCGCGTCGTCCGGCGAGAGGCCAGGGAGGTGTCCATCCCTCCCCACCTCATGTCAGGCAGCGCCTCGGAGCTGTTTGGCTTCATCGCCTCCGCGCTGGCCAAGTTCGTCGCCGACGAAGGCCACAGCGGCGCGTTGGACGGCGGCAGGCAGAGGGAGCTGGGGTTCACCTTCTCCTTCCCCGTGAGGCAGTCGTCCATCGCGTCCGGCACGCTGATCAAGTGGACCAAGGCGTTTTCGATCGATGATGCGGTGGGCGAAGATGTGGTAGCTGAACTGCAGACGGCCATGGAGAAGCAAGGTCTTGACATGCGCGTGTCCGCATTGATCAATGACACCGTCGGGACACTGGCTGCGGGCAGCTACTACGACGAAGATGTGGTTGTCGGCGTGATATTAGGTACTGGCTCGAACGCCGCTTATGTCGAAAAGGCAAATGCCATACCAAAGTTGGAAGGCGAGCTACCAAAATCAGGAAATATGGTTATCAATACAGAATGGGGCAACTTCAGTTCATCGTGCCTTCCGATAACGGAATATGATGAAGCATTGGATGAGGAGAGCTTAAACCCGGGGGAGCAGATCTTCGAGAAGTTGATTTCAGGGATGTACCTAGGCGAAATCGTGAGGAGGGTGCTTCTTAAAATCTCCTCGCAGTCTTCGATTTTCGGCAATATCAAACGCACCATGCTCAACACTCGCTTCATCCTGAGGACCCCTGATATATCTGCGATGCACCACGACGAAACACCTGATCTGAGGATTGTCGCCGAAAAACTGGCAGAGAACCTGAAGATCAAAGGCACGTCCTTAGAGACGAGGAAGATGGTCGTCGAAATCTGCGATATTGTGACCAGTAGGTCTGCCCGGCTGGCTGCAGCTGGGATTGTTGGGATCCTCAGGAAGATTGGCAGAGGCACCCCCGGCGACGACCGGAAGACGGTCATCGCCATCGACGGCGGCCTCTTTGAGCATTACACCGAATTCCGGCAGTGCCTGGAGAGCACGCTGGGCGGGTTGCTCGGCGAGGAGGCGTCCAAGTTGGTGTCTGTCAAGCTCGCAAACGACGGGTCGGGTCTGGGAGCTGCCCTGATTGCAGCTGCTCACTCTCAGTATCTGAATTGA

>PeHXK8

ATGGCCGCAGCTGCGGTCGCAATGGCGGAGCAGGTGGTGGCGGACTTCCGGGAGAAGTGCACAACGCCGGCATCGCTGCTGCGCGATTTGGCGTCGGCGATGGCCGACGAGATGGGCGCGGGCCTGGAGAAGGAGGGTGGGAGCAGGGTCAAAATGCTCCTCTCCTACGTCGATAAGCTCCCCACTGGGAGGGAGGAAGGTTTGTTCTATGGATTGGACCTAGGAGGAACGAACTTCCGCGTTTTGAAGGTGCAGCTGGGCGGCAATAAGAAGCATGTCGTGAACCGTGAGTCCAGAGAAGTCACCATTCCACCCCATTTGATGTTAGGGAGCTCCTCTGAACTGTTTGGTTTCATTGCATCTGAATTAGCAAAGTTTGTTGCTGATGAAGAGAAGTGTGCTAACTCATCAAACCGGAAGCAACGAGAACTAGGATTCACATTTTCTTTCCCAGTGAGGCAGCGTTCTGTTGCATCGGGTACCCTTGTCAAGTGGACAAAAGCATTTTCTATTGATGATGCTGTAGGTGAAGATGTAGTGGCTGAACTGCAAACGGCTATGGAGAAGCAAGGTCTGGACATGCATGTGGCAGCATTGATTAATGATGCTGTTGGGACGTTGGCTGGAGCCAGATACTACGATGAAGATGTTGTCGCAGGTGTGATATTTGGTACTGGCACAAATGCCGCATATGTTGAGAAGGTAAATGCTATACCAAAATGGGAAGGAGAGCTGCCTAGTTCAGGGGATATGATCATCAATATGGAATGGGGTAACTTCTATTCATCCCATTTTCCAGTCACTGAATACGATCAAGCATTAGATAAGGAAAGCTTAAATCCCGGAGAGCAGATCTACGAGAAGTTAACATCAGGAATGTATTTAGGTGAAATTGCTAGGAGGGTGCTGCTTGAAATGTCCTTGCAATCTGCAATTTTCGGCGATATTGATCACACTAAGCTCAAAACTCATTTCCTCCTGCGGACTCCACACATTTCTGCAATGCACCATGACGAAACCCCTGATCTGAAGGATGTGGCTGAAAAACTGGAAGAAAACCTAGAGATTACAGGCACATCCTTAGAGACAAGAAAAATGGTTGTCGAAATCTGTGACATTGTGTCAAGAAGGGCAGCCCGGCTGGCCGCTGCAGGGGTTGCAGGAATCCTCAAGAAGCTTGGGAGAGATGGCCCCATCGGCAAGCAGCGTTCAGTCATTGCCATTGATGGCGGATTGTTCGAACACTACACCAAATTCCGCGAATGCTTGGAAAGCACACTGGATGAGTTGCTGGGAGAGGAGGCATCGAAGTCGGTAGCCGTCAAGCACATGGGTGATGGTTCAGGGATAGGGGCTGCCCTGATTGCAGCTTCTCAATCTCAGTACAAGTATGTTGAGCAACAGTAG

>PeHXK9

ATGGTGGAGGAGATGGCGCGCGGGCTGCGCGCCGACCCCCACGCCTCGCTCAAGATGCTCATAAGCTACGTCGACAACCTCCCCACTGGGGATGAGCATGGATTGTTTTATGCACTGGATCTTGGTGGGACCAACTTCCGTGTTATACGGGTTCAGCTTGGAGGAAGGGAGAAGCGTGTTGTCATGCAACAATACGAAGAGGTGTCCATTCCACCTCATCTGATGGTTGGGGCTTCCACGGAACTGTTTGATTTCATTGCGGCTGAGTTGGAAAAATTTGTCGAGACTGAAGGAGAAGATTTCCACTTGCCAGTGGGCAGGCAGAGAGAACTGGGTTTCACCTTTTCTTTCCCAGTGCACCAGACATCAATATCATCAGGCACTCTCATTAAGTGGACAAAGGGATTTTCCATCAATGGCACGGTCGGGGAAGATGTTGTGGCTGAATTGAGCAGGGCTATGGAGAGGCAGGGTCTTGATATGAAAGTTACAGCTCTGGTTAATGACACTGTGGGCACATTGGCTGGTGGGAGATATGTTGATAATGATGTTGTTGCTGCTGTAATATTGGGCACTGGCACAAATGCAGCATACGTGGAGCACGCAAATGCAATTCCAAAATGGAATGGGCTACTACCTAGATCAGGAAATATGGTAATCAACATGGAATGGGGAAACTTCAAGTCAGATAAGCTTCCTCATTCAGAATATGATAGTGCCTTGGATTTTGAAAGTTTGAACCCTGGCGAGCAGATATACGAAAAGATGATTTCTGGCATGTATCTTGGAGAGATTGTGCGAAGAATTTTGCTGAAGCTGGCTCATGATGCTTCATTGTTTGGGGATGTTATTCCACCAAAATTGGAGCAACTATTTATACTGAGGACACCGGATATGTCAGCCATGCATCATGACACCTCACATGATCTCAAAAATCTGGGGGCTAAGCTGAAGGATATCCTGGGGGTCGCTGATACTTCCCTGGAAGCAAGATACATTACTCTTCACATCTGCGACCTTGTCGCAGAGAGAGGTGCACGCTTGGCTGCTGCTGGTATATATGGCATTCTAAAGAAGCTGGGCAGGGATAGAGTGAAAAGTGACGGTAGTCAAAAGCAAAGGACTGTCATTGCTATGGACGGTGGTCTCTACGAGCATTACAAGAAGTTCAGCACCTGCCTAGAATCGACTCTTGCAGACCTGCTCGGGGAGGAGGCTGCCTCTTCGGTTGTTGTCAAGTTGGCTAATGATGGCTCCGGCATTGGTGCTGCACTTCTTGCAGCCTCACACTCCCAACACATAAATGAGTGTGCTTGTGCAGTGGGATATAGCGAGTATGACGCCAATGAGTTTGGAGTATATCACATGGAACCAGCGTTTGCAACTGCAGGAAAGTGGAAACGGGGGTTTGAATGCTGCCGTTCCTTTCCTGTCAATTCTTTTGCCCCTTGCCCTCAAGGCACCGATGGGGAGTTCCGGCAGCTCGACGGCGACGGCAACGGGAGGCTGTCGGTGAGGGAGCTCCAGCCGGCCGTCGCGGATATCGGCGCCGCCATCGGGCTGCCCGCGCGGGGGTCGTCGGCGCAGGTGGAACACATCTGCTCAGAGAGCAAGAGTGTATTGCATATACTGTTGTTTTCATCACGAAGCCATACAAAAGAGGAAGCAGATTTTATCTAG

>PeHXK10

ATGTTCGCGGGGCTCGCGTCCGAGGGCGCCAGCAAGGTCCGGATGCTGCTCACCTGCGTCGACGCGCTCCCCGATGGGAGTGAGGAAGGCATCTATTATGCCATTGATCTTGGTGGGACGAGCTTTAGAGTCTTGAAACTAGAATTTGGTGCAGGGTCTATGATCATTAATAAGAAAGTTGAACATCATCCTATCCCTGAAGAATTGACTAAGGGTACAAGCCAGGATCTGTTCAATTTCATTGCCTCAGCACTAAAGAATTTTATTCAAAGGGAGGATGGGAAAGATGTGGGGAGGGAACTTGGTTTTACATTTTCTTTCCCTGTCAGACAACTTTCCATATCCTCAGGGTCATTAATTAGGTGGACTAAAGAATTTTCAATTGAAGAGGCTGTCGGGAAAGATGTTGCTCAGTGCTTAAATGAAGCCCTTGTTAGGAATGGACTGAATTTGCAGGTCAATGCACTGGTGAACAATACTGTGGGGACATTAGCTCTGGGGCATTATTATGATGAGGATACAGTGGCTGCAGTGATTATTGGAGCTGGCACCAATGCTTGCTATATTGAACGCAATGAGGCAATTACAAAAGGTTGTGGTGTTCTTACCAACTCTGGACTAATGGTTATAAATGTGGAATGGGGGAGTTTCCGGCCTCCGCAAATACCATTAACTCCTTATGACATATGTTTCAATGATGAGAAACAAGATTACTATGACCAGGGTTTTGAGAAAATGATCTCCGGTGTGTATCTTGGGGAAATTGCAAGATTGGTGTTCCAAAAAATGGCTCAAGAGTCAGATGTATTTGGTATTGCTGCTGATGGTTTATCCACCCCTTTCATCTTAAGTACACCATGTCTAGCTGCTATTCGTGAGGATGATTCCCCAGATTTGAGAGAAGTCGGAAGGATACTGGAAGAACATCTGAAGATACCAGATGTTCATCTGAAGACTCGGAGGCTTGTCCAGAGAGTCTGTGACATTGTAACCCGAAGAGCGGCCCGTCTAGCGGCAGCTGGAATTGTTGCAATACTGCAAAAAATCGGTCGTGATGGAACCCTTTGTGGTACCAACAAAGTTCGAAGAATAACAGGCGTGCCGAAGAGATCGGTCATTGCAATCGAGGGTGGCCTGTACCAAGGCTATTCAGTCTTCAGAGAGTATCTGAATGAAGCCGTAAGCGAGATCCTAGGGGAGGAGATTGCGGCCACTGTTAGTCTTAGAGTGATGGAGGAGGGGTCCGGGATTGGGGCCGCCCTCCTTGCAGCTGCATATTCGTCAAATATGCAAAAGTAA
