# Supplementary material for: Genome-Wide Identification and Characterization of Hexokinase Genes in Moso Bamboo (Phyllostachys edulis)
Source: Front Plant Sci. 2020 May 19;11:600. doi: 10.3389/fpls.2020.00600 (PMC7248402; doi:10.3389/fpls.2020.00600)
Supplement: FILE S3 — Protein sequences of PeHXKs. [file Data_Sheet_3.docx]

Protein sequences of PeHXKs

>PeHXK1 MEKQGLDMHVAALINDAVGTLAGARYYDEDVVAGVIFSTGTNAAYVEKANAIPKWEGELPNSGDMVINMEWGNFCSSHLPVTEYDQALDKEIPESRRIYEKLISGIYLGEIVRRVLLKMSPQSAIFGDIDHTKLKTHFLLRLQAQTRKLVVEICDIVARRAARLAAAGILTKLGRDCSVDKQRSVIAIDGGLFEHYTKFRECLESTLDELLGEEASKSVAVEHADDGSGVGAALIAAAQS

>PeHXK2 MRKAAAAVIATVEAVGVALVVRRQLREAKRWARTAAVLRELEERCAARPARLRQVADVMAVEMHAGLTSEGRSKLKMIIGYVDSLPSGAYIWRNGQDLVDKFDRFKELFDFIAAALAKFVASEGEDFYLPGGRQRELGFTFSFPVKQSSIASGTLIKWTKGFAIDETVGEDVVAELGRALERQGIDMKVTALVNDTIEALAGGRYDDNDVVAAVILGTGTNAAYVDRANAIPQWHGLLPKSGYMVINMEWGNFRSSHLPLTEFGPALDAESLNPGEQVSYKRASILVGISSSRVLLKMNQEASVFGDVVPPKLKIPFIL

>PeHXK3a MGRVGFGVAVGCAAVTCAIAAALVARRASARARWRRAVALLREFEEGCATPPARLRQVVDAMVVEMHAGLASDGGSKLKMLLTFVDALPNGNEEGIYYAIDLGGTNLRVLRVEVGAGSVIVNRKVEHQPIPEELTNGTTEDLFNFVALAQKNFVEREDGNDEKKALGFTFSFPVRQNSVSSGSLIRWTKRFSIEDTVGKDVAQCLNEALARCGLNMRVTALVNDTVGTLALGHYYDEDTVAAVIIGAGTNACYIERTDAIIKCQGLLTNSGGMVVNMEWGNFWSSHLPRTPYDISLDDETQNRNDQGFEKMISGMYLGEIARLVLHRMAQESDVFGDAADGLSTPFILSTPFLAAIREDDSPDLSEVRMILREHLKIPDAPLKTRRLVVKVCDIVTRRAARLAAAGIAGILKKLGRDGSGAASSGRTRGQPKRTVVAIEGGLYQGYPVFREYLDEALVEILGEEVARNVTLRVTEDGSGIGAALIAAVHSSNRQQQGGSI

>PeHXK3b MVVEMHAGLASDGGSKLKMLLTFVDALPNGNEEGIYYAIDLGGTSIRVLRVEVGAGSVIINRKVEHQPIPEELTKGTTESLFNFVALALKNFVEKEDGKDEKRALGFTFSFPVRQNSVSSGSLIRWTKGFSIEDTVGKDVAQCLNEALARCGLNVRVTALVNDTVGTLALGHYYDEDTVAAVIIGAGTNACYIERTDAIIKCQGLLTNSGGMVVNMEWGNFWSSHLPRTPYDISLDDKTQNRNDQGFEKMISGMYLGEIARLVLHRMAHESDVFGDAADILSTPFILSTPRLAAICEDDSPDLSEVRRILQEHLKIPDAPLKTRRLVVKVCDIVTRRAARLAAAGIVGILKKLGRDGSGAASSGRTRGQPKRTVVAIEGGLYQGYPVFREYLDEALEEILGEEVARNVTLRVTEDGSGVGAALLAAVHSLNRQQQGGSI

>PeHXK4 MSAAVCSPIPAATVAQHRRRCGAAVRCSAVAAPILNDLRLQCATPLPVLRCVADAMAADMRAGLAADGAGELKMIPSYVYSLPTGDETGLFYALDLGGTNFRVLRVQLGGKDKRVVDTEFEQVSIPKEIMHGTTEELFDFIASGLSKFVAKESDKVCLPQGWKREIGFTFSFPVKQTSIDSGILIKWTKGFAVCGTAGKDVVACLNAAMERRGLDMRVSALVNDTVGTLAGARYWDDDVMVAVILGTGTNACYIERTDAIPKLQNVMPGAGNTIINTEWGAFSEGLPLTEFDRDMDDESINPGEQIFEKTISGMYLGEIVRRVLVKMAKVSDLFGNSFPDRLAMPFVLRTPHLCAMQQDSSDDLGEVKLILNDIICVKQSSMEARRIIVEACDCIVKRGGRLAGAGIAGILQKMENDSKGLIFGQRTVVAMDGGLYENYPQYGEYMKEAVVELLGPEDSKHIVVEHTKDGSGIGAALLAAANSKYAAQLST

>PeHXK5a

MGKAAAVGTAVVVCAAVGVAVVLARRRRQRKAELVDAAEADRKRRVAAVIEEVESRLATPTALLRSISDAMVSEMERGLCGDIHATLKMLITYVDNLPTGDEHGLFYALDLGGTNFRVLRVQLGGREKRVVKQQYEEVSIPPHLMVGTSMELFDFIAAALAKFVDTEGDDFHLPEGRQRELGFTFSFPVNQTSISSGTLIKWTKGFSINGTVGEDVVSELSNAMERQGLDMKVTALVNDTVGTLAGGRYMDNDVVAAVILGTGTNAAYVEYANAIPKWNGLLPRSGNMVINTEWGSFKSDKLPLSEFDKAMDFESLNPGEQIYEKMISGMYLGEIVRRILLKLAHDASLFGDVVPTKLEQPFVLRTPDMSAMHHDSSHDLKILGAKLKDVVGVPNTSLEVRYITRHICDIVAERGARLAAAGIYGILKKLGRDKVPSDGSKMPRTVIALDGGLYEHYKKFGSCLEATLTDLLGEDASSSVVAKLANDGSGIGAALLAASHSQYAEVD

>PeHXK5b MGKAAAVGTAVVVCAAVGVGVVLARRRWRRLAELVEAAEADRKRRVAAVIEEVEQRLATPTALLRSISDAMVSEMERGLRGDIHATLKMLITYVDNLPTGDEHGLFYALDLGGTNFRVLRVQLGGREKRVVKQQYEEVSIPPHLMVGTSMELFDFIAAALAKFVDTEGDDFHLPEGRQRELGFTFSFPVNQTSISSGTLIKWTKGFSINGAVGEDVVSELSKAVERQGLDMKVTALVNDTVGTLAGGRYMDNDVVAAVILGTGTNAAYVEHANAIPKWNGLLPRSGNMVINTEWGSFKSDKLPLSEFDKAMDFESLNPGEQIYEKMISGMYLGEIVRRILLKLAHNASLFGDVVPPKLEQPFVLRTPDMSAMHHDSSHDLKILGAKLKDIVGVPDTSLEVRYITRHICDIVAERGARLAAAGIYGILKKLGRDKVPSDGSKMPRTVIALDGGLYEHYKKFSSCLEATLTDLLGEDPSCSVVAKLANDGSGIGAALLAASHSQYAEVH

>PeHXK6 MVEEMARGLRADPHAPLKMLISYVDNLPTGDEHGLFYALDLGGTNFRVIRVQLGGREKRVIMQQYDEVSIPPHLMVGTSTELFDFIAAELEKFVETEGEDFHLPEGRQRELGFTFSFPVHQTSISSGTLIKWTKGFSINGTVGEDVVAELSRAVERQGLDMKVTALVNDTVGTLAGGRYVDNDVVAAVILGTGTNAAYVEHANAIPKWNGLLPRSGNMVINMEWGNFKSDKLPCSEYDSALDFESLNPGEQIYEKMISGMYLGEIVRRILLKLAHDASLFGDVVPPKLEQLFILRTPDMSAMHHDTSHDLKHLGAKLKDILGVADTSLEARYITLHICDLVAERGARLAAAGIYGILKKLGRDRVPSDGSQKQRTVIAMDGGLYEHYKKFSACLESTLADLLGEAASLVVVKLANDGSGIGAALLAASHSQYADVEYS

>PeHXK7 MAAAAAAVAEQVVEELREACATPAPRLSDVAAAMEEEMTAGLAEEGGSKIKMIISYVDNLPNGSEEGLFYALDLGGTNFRVLRVQLAGKEKRVVRREAREVSIPPHLMSGSASELFGFIASALAKFVADEGHSGALDGGRQRELGFTFSFPVRQSSIASGTLIKWTKAFSIDDAVGEDVVAELQTAMEKQGLDMRVSALINDTVGTLAAGSYYDEDVVVGVILGTGSNAAYVEKANAIPKLEGELPKSGNMVINTEWGNFSSSCLPITEYDEALDEESLNPGEQIFEKLISGMYLGEIVRRVLLKISSQSSIFGNIKRTMLNTRFILRTPDISAMHHDETPDLRIVAEKLAENLKIKGTSLETRKMVVEICDIVTSRSARLAAAGIVGILRKIGRGTPGDDRKTVIAIDGGLFEHYTEFRQCLESTLGGLLGEEASKLVSVKLANDGSGLGAALIAAAHSQYLN

>PeHXK8 MAAAAVAMAEQVVADFREKCTTPASLLRDLASAMADEMGAGLEKEGGSRVKMLLSYVDKLPTGREEGLFYGLDLGGTNFRVLKVQLGGNKKHVVNRESREVTIPPHLMLGSSSELFGFIASELAKFVADEEKCANSSNRKQRELGFTFSFPVRQRSVASGTLVKWTKAFSIDDAVGEDVVAELQTAMEKQGLDMHVAALINDAVGTLAGARYYDEDVVAGVIFGTGTNAAYVEKVNAIPKWEGELPSSGDMIINMEWGNFYSSHFPVTEYDQALDKESLNPGEQIYEKLTSGMYLGEIARRVLLEMSLQSAIFGDIDHTKLKTHFLLRTPHISAMHHDETPDLKDVAEKLEENLEITGTSLETRKMVVEICDIVSRRAARLAAAGVAGILKKLGRDGPIGKQRSVIAIDGGLFEHYTKFRECLESTLDELLGEEASKSVAVKHMGDGSGIGAALIAASQSQYKYVEQQ

>PeHXK9 MVEEMARGLRADPHASLKMLISYVDNLPTGDEHGLFYALDLGGTNFRVIRVQLGGREKRVVMQQYEEVSIPPHLMVGASTELFDFIAAELEKFVETEGEDFHLPVGRQRELGFTFSFPVHQTSISSGTLIKWTKGFSINGTVGEDVVAELSRAMERQGLDMKVTALVNDTVGTLAGGRYVDNDVVAAVILGTGTNAAYVEHANAIPKWNGLLPRSGNMVINMEWGNFKSDKLPHSEYDSALDFESLNPGEQIYEKMISGMYLGEIVRRILLKLAHDASLFGDVIPPKLEQLFILRTPDMSAMHHDTSHDLKNLGAKLKDILGVADTSLEARYITLHICDLVAERGARLAAAGIYGILKKLGRDRVKSDGSQKQRTVIAMDGGLYEHYKKFSTCLESTLADLLGEEAASSVVVKLANDGSGIGAALLAASHSQHINECACAVGYSEYDANEFGVYHMEPAFATAGKWKRGFECCRSFPVNSFAPCPQGTDGEFRQLDGDGNGRLSVRELQPAVADIGAAIGLPARGSSAQVEHICSESKSVLHILLFSSRSHTKEEADFI

>PeHXK10 MFAGLASEGASKVRMLLTCVDALPDGSEEGIYYAIDLGGTSFRVLKLEFGAGSMIINKKVEHHPIPEELTKGTSQDLFNFIASALKNFIQREDGKDVGRELGFTFSFPVRQLSISSGSLIRWTKEFSIEEAVGKDVAQCLNEALVRNGLNLQVNALVNNTVGTLALGHYYDEDTVAAVIIGAGTNACYIERNEAITKGCGVLTNSGLMVINVEWGSFRPPQIPLTPYDICFNDEKQDYYDQGFEKMISGVYLGEIARLVFQKMAQESDVFGIAADGLSTPFILSTPCLAAIREDDSPDLREVGRILEEHLKIPDVHLKTRRLVQRVCDIVTRRAARLAAAGIVAILQKIGRDGTLCGTNKVRRITGVPKRSVIAIEGGLYQGYSVFREYLNEAVSEILGEEIAATVSLRVMEEGSGIGAALLAAAYSSNMQK
